# Supplementary material for: The anti-GD2 monoclonal antibody naxitamab plus GM-CSF for relapsed or refractory high-risk neuroblastoma: a phase 2 clinical trial
Source: Nat Commun. 2025 Feb 14;16:1636. doi: 10.1038/s41467-025-56619-x (PMC11828896; doi:10.1038/s41467-025-56619-x)
Supplement: Supplementary file 1 — Supplementary Information [file 41467_2025_56619_MOESM1_ESM.pdf]

SUPPLEMENTARY INFORMATION

The anti-GD2 monoclonal antibody naxitamab plus GM-CSF for relapsed or refractory high-risk neuroblastoma: a phase 2 clinical trial

Jaume Mora, Godfrey C. F. Chan, Daniel A. Morgenstern, Loredana Amoroso, Karsten Nysom, Jörg Faber, Arthur Wingerter, Melissa K. Bear, Alba Rubio-San-Simon, Blanca Martínez de Las Heras, Karen Tornøe, Maria Düring, & Brian H. Kushner

Supplementary Fig. 1. Recommended supportive therapies available at bedside during naxitamab treatment in Trial 201

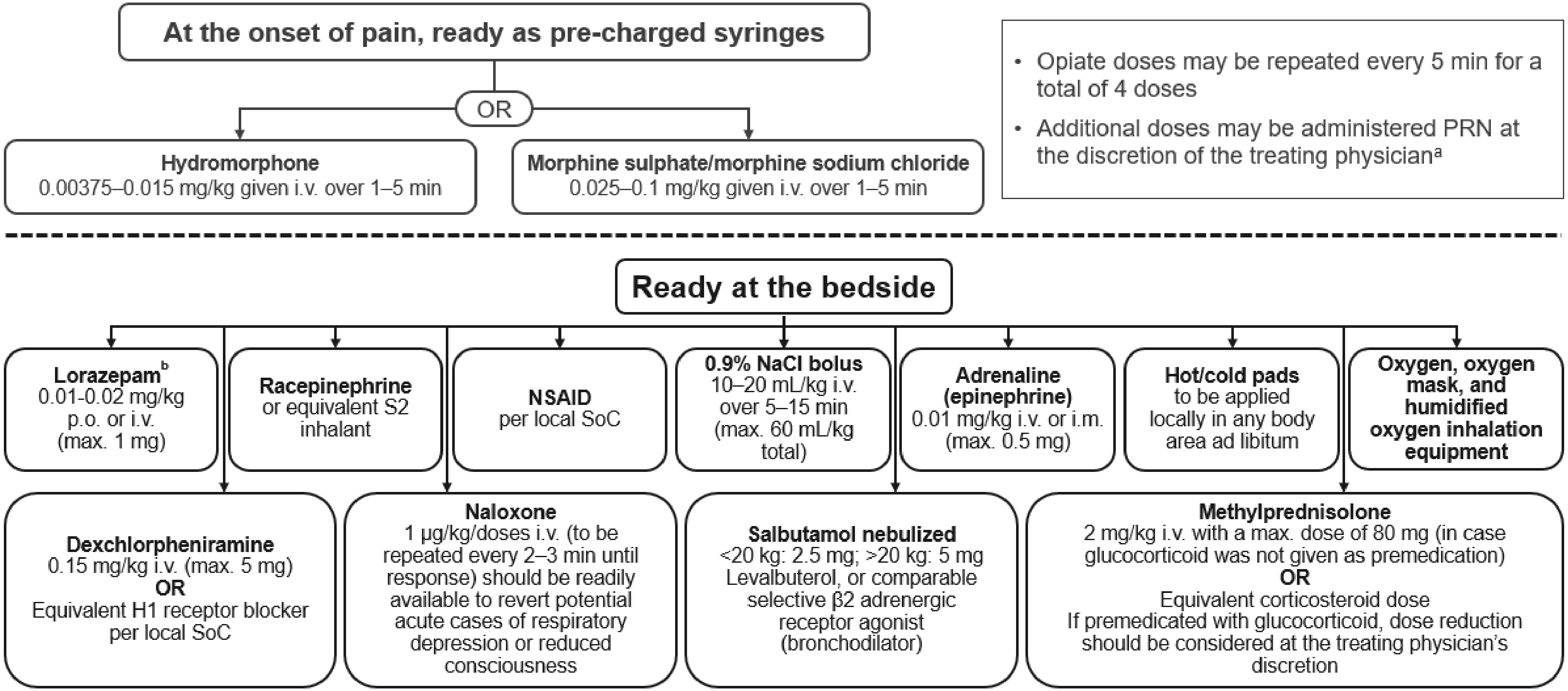

Supportive therapy selection was at the discretion of the principal investigator. Treatments and doses listed were suggested, not required. [a]If pain control was inadequate with opioids, ketamine could be used administered to local SoC. [b]Or equivalent per local SoC. **Abbreviations:** GM-CSF, granulocyte-macrophage colony-stimulating factor; i.m., intramuscular; i.v., intravenous; max., maximum; NSAID, non-steroidal anti-inflammatory drug; p.o., oral; PRN, as needed; SoC, standard of care.

Supplementary Fig. 2. Investigational naxitamab + GM-CSF. a Each investigational cycle of naxitamab + GM-CSF. **b** Treatment and response schedule.

a

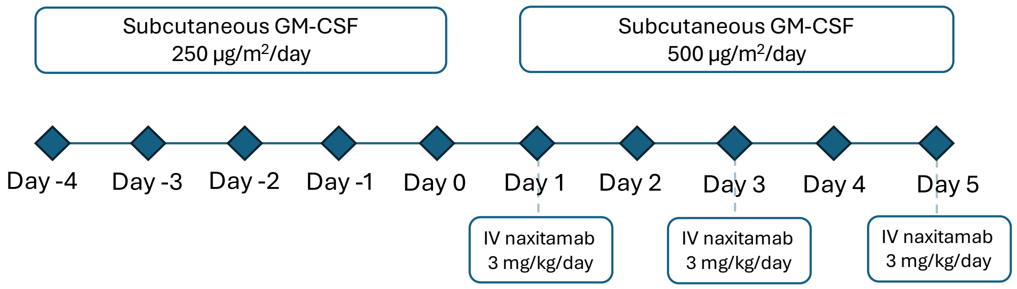

b

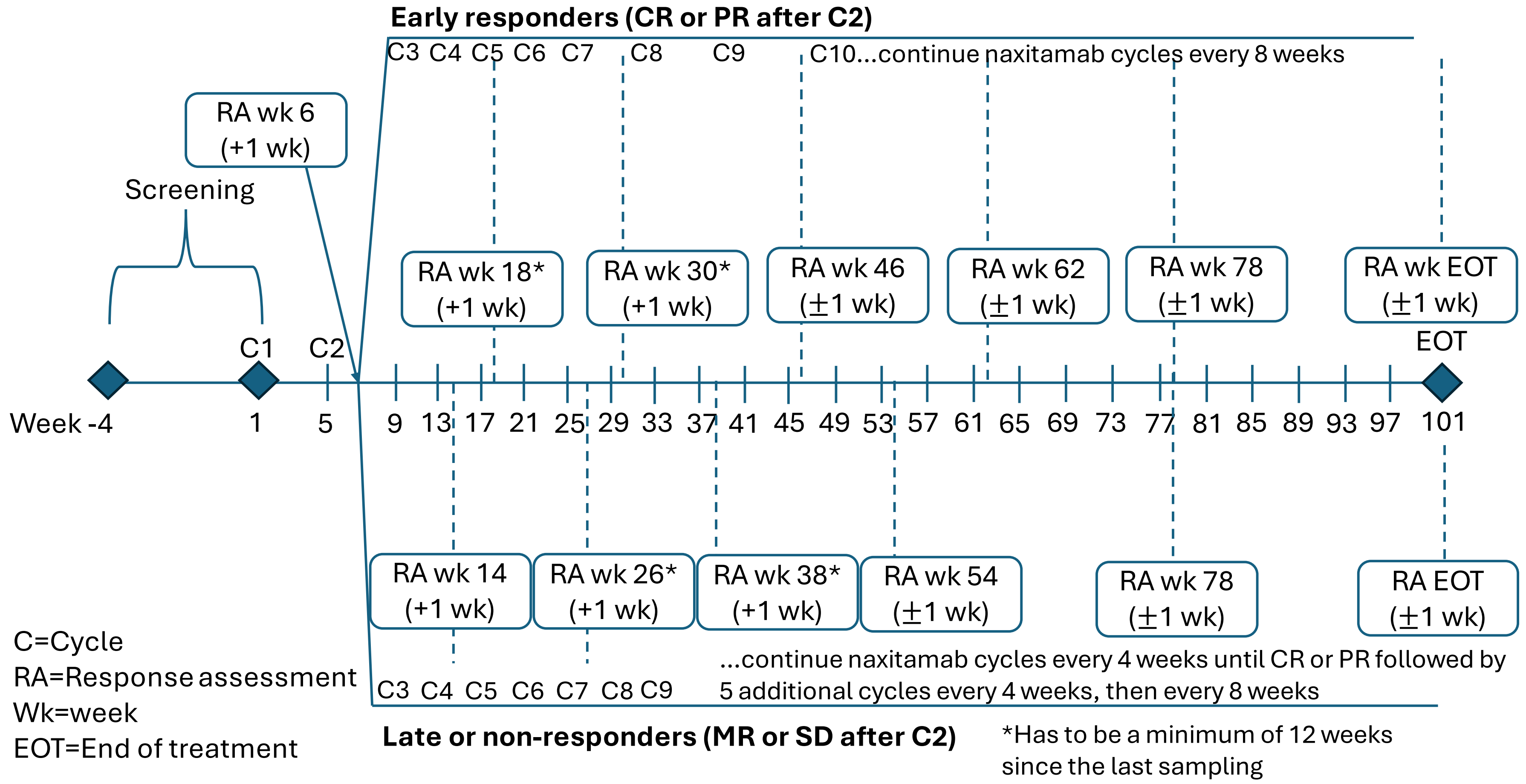

Abbreviations: CR, complete response; GM-CSF, granulocyte-macrophage colony-stimulating factor; MR, minor response; PR, partial response. SD, stable disease.

Supplementary Table. 1 Per-site patient enrollment during Trial 201

Location	Patients enrolled, n
**Canada**	
The Hospital for Sick Children, Toronto, ON	**4**
**Denmark**	
Rigshopitalet, Copenhagen	**3**
**Germany**	
JGU Universitatsmedizin, Mainz	**3**
**Hong Kong**	
Queen Mary Hospital	**2**
Hong Kong Children’s Hospital	**8**
**Italy**	
IRCCS Istituto Giannina Gaslini, Genoa	**4**
**Spain**	
Hospital Sant Joan de Déu, Barcelona	**34**
Hospital Infantil Universitario Niño Jesús, Madrid	**1**
Hospital Universitario y Politécnico La Fe, Valencia	**1**
**United States of America**	
Memorial Sloan Kettering, New York, NY	**13**
Riley Hospital for Children, Indianapolis, IN	**1**

Supplementary Table 2. Additional response rates by *MYCN* status and sex

Patient population	Endpoint	Patients	
		***n* (%)**	**95% CI, %**
***MYCN* not amplified (*n* = 38)**	CR	15 (40)	24–57
	PR	3 (8)	2–21
	MR	3 (8)	2–21
	SD	10 (26)	13–43
	PD	4 (11)	3–25
	NE	3 (8)	–
**ORR**	CR + PR	18 (47)	31–64
***MYCN* amplified (*n* = 7)**	CR	1 (14)	0–58
	PR	2 (29)	4–71
	PD	4 (57)	18–90
**ORR**	CR + PR	3 (43)	10–82
**Male (*n* = 31)**			
**ORR**	CR + PR	15 (48)	30–67
**Female (*n* = 21)**			
**ORR**	CR + PR	11 (52)	30–74

Abbreviations: CI, confidence interval; CR, complete response; CS, Curie score; mAb, monoclonal antibody; ORR, overall response rate; PR, partial response.

Supplementary Table 3. Summary of median pain scores: FLACC scale and Wong–Baker FACES scale

Scale and Cycle	Infusion	Pre-Infusion	During	15 min after infusion
**FLACC Scale (201 data, *n* = 33)**				
**1**	1	0	8	0
	2	0	8	0
	3	0	8	0
**2**	1	0	8	0
	2	0	8	0
	3	0	8	0
**Wong–Baker FACES Scale (201 data, *n* = 41)**				
**1**	1	0	8	0
	2	0	8	0
	3	0	8	0
**2**	1	0	6	0
	2	0	6	0
	3	0	6	0

Abbreviations: FLACC, Face, Legs, Activity, Cry, Consolability.

Supplementary Table 4. Time to resolution of Grade 3 treatment-related pain events (E=412)

Time to resolution	Pain events, %
**Less than or equal to 1 hour**	52%
**Less than or equal to 2 hours**	61%
**Less than or equal to 5 hours**	90%
**Less than or equal to 1 day**	99%

Abbreviations: E, total number of reports of the event. Events of pain includes all AEs reported with a preferred term which includes the word pain, excluding procedural pain and vessel puncture pain.

Supplementary Table 5. Related AEs that lead to treatment discontinuation in Trial 201

AE	Related SAEs, *n* (%)	Related non-serious AEs, *n* (%)	Grade
**Anaphylactic reaction**	2 (3)	—	4
**Respiratory depression**[a]	1 (1)	—	4
**PRES**	1 (1)	—	3
**Myocarditis**[b]	1 (1)	—	3
**Hypotension**[a]	—	1 (1)	2
**Urticaria**	1 (1)	—	2

[a]The events of respiratory depression and hypotension occurred in the same patient.

[b]The patient with myocarditis discontinued trial treatment. However, naxitamab was continued at a lower dose (2.4 mg/kg/day) without recurrence of myocarditis.

Abbreviations: AE, adverse event; PRES, posterior reversible encephalopathy syndrome; SAE, serious adverse event

Supplementary Table 6. Listing of IRBs and IECs for Trial 201

Clinical Trial Site	Name and Address of IRB
Memorial Sloan Kettering Cancer Center<br>1275 York Avenue<br>New York, NY 10065	Memorial Sloan Kettering Cancer Center Institutional Review Board/Privacy Board<br>1275 York Avenue<br>New York, NY 10065
Riley Hospital for Children<br>705 Riley Hospital Drive<br>Ped/Hem/Onc MSA<br>Indianapolis, IN 46202	Indiana University Institutional Review Board<br>986 Indiana Avenue, 5th Floor<br>Indianapolis, IN 46202
Rigshospitalet<br>Blegdamsvej 9<br>Copenhagen, Denmark 2100	De Videnskabsetiske Komitéer<br>Kongens Vænge 2<br>Hillerød, Denmark 3400
Hospital Sant Joan de Déu<br>Passeig de Sant Joan de Déu<br>Esplugues de Llobregat<br>Barcelona, Spain 08950	Fundació Sant Joan de Déu<br>C/ Santa Rosa, 39-57, 4a planta<br>Esplugues de Llobregat<br>Barcelona, Spain 08950
Hospital Infantil Universitario Niño Jesús Av. de Menéndez Pelayo, 65<br>Madrid, Spain 28009	Fundació Sant Joan de Déu<br>C/ Santa Rosa, 39-57, 4a planta<br>Esplugues de Llobregat<br>Barcelona, Spain 08950
Hospital Universitario y Politécnico La Fe<br>Avinguda de Fernando Abril Martorell 106<br>Valencia, Spain 46026	Fundació Sant Joan de Déu<br>C/ Santa Rosa, 39-57, 4a planta<br>Esplugues de Llobregat<br>Barcelona, Spain 08950
Hospital for Sick Children<br>555 University Ave<br>Toronto, Canada M5G 1X8	Hospital for Sick Children Research Ethics Board<br>555 University Ave<br>Toronto, Canada M5G 1X8
Queen Mary Hospital<br>Pok Fu Lam Rd,<br>Pok Fu Lam, Hong Kong	Institutional Review Board of the University of Hong Kong<br>Room 901, 9/F, Administration Block, Queen Mary Hospital, 102 Pokfulam Road<br>Pok Fu Lam, Hong Kong
Hong Kong Children's Hospital<br>1 Shing Cheong Road,<br>Kowloon Bay, Hong Kong	Hong Kong Children’s Hospital Research Ethics Committee<br>8/F, Tower A, Hong Kong Children’s Hospital, 1 Shing Cheong Road,<br>Kowloon Bay, Hong Kong
JGU Universitätsmedizin<br>Langenbeckstraße 1<br>Mainz, Germany 55131	Landesärztekammer Rheinland-Pfalz<br>Deutschhausplatz 3<br>Mainz, Germany 55116

Clinical Trial Site	Name and Address of IRB
IRCCS Istituto Giannina Gaslini<br>Via Gerolamo Gaslini, 5<br>Genova, Italy 16147	Ethics Committee of the Liguria Region<br>Largo Rosanna Benzi 10<br>Genova, Italy 16132

Abbreviations: IECs, independent ethics committees; IRBs, institutional review boards.

Supplementary Table 7. Inclusion and exclusion criteria for Trial 201 per study protocol at data cutoff

Inclusion criteria	Exclusion criteria
1. Documented diagnosis of NB as defined per INRG as:<br>a. Histopathology of tumor biopsy, or<br>b. BM aspirate or biopsy indicative of NB by histology, plus high blood or urine catecholamine metabolite levels or *MYCN* amplification, or<br>c. MIBG-avid lesion(s)<br><br>2. High-risk NB patients with either primary refractory disease or incomplete response to salvage treatment (in both cases including SD, MR, and PR) evaluable in bone and/or BM as defined per protocol. If disease is only present in bone, the patient must have evaluable disease outside the radiation areas for being eligible in the trial. If disease is only present in the BM, the involvement must be >5%<br><br>3. Life expectancy ≥6 months<br><br>4. Age ≥12 months<br><br>5. Acceptable hematological status at screening (hematological support is allowed if administered ≥1 week before first screening procedure) defined as:<br>a. Hemoglobin ≥8 g/dL (5 mmol/L)<br>b. White blood cell count ≥1,000/µL (1.0×10^9/L)<br>c. ANC ≥500/µL ($0.5\text{x}10^9$/L)<br>d. Platelet count ≥25000/µL (25×10^9/L)<br><br>6. Acceptable liver function defined as:	1. Any systemic anticancer therapy, including chemotherapy or immunotherapy, within 3 weeks of first dose of GM-CSF<br><br>2. Evaluable NB outside bone and BM defined as:<br>a. MIBG-avid tumor: Definite MIBG uptake in tumor tissues outside bone and BM<br>b. MIBG non-avid tumor: Definite uptake in tumor tissues outside bone and BM on FDG-PET<br><br>3. Actively progressing disease at trial entry according to Park criteria[1]<br><br>4. Existing major organ dysfunction CTCAE Grade >2, with the exception of hearing loss, hematological status, and kidney and liver function<br><br>5. Active life-threatening infection<br><br>6. Prior treatment with naxitamab<br><br>7. Karnofsky/Lansky score <50%<br><br>8. Pregnancy or a woman who is breastfeeding (females of child-bearing potential must have a negative pregnancy test at screening). A woman of child-bearing potential is excluded if she does not agree to use highly effective contraception for a period of 40 days after the last naxitamab infusion according to protocol. A sterilized or infertile woman is exempt from the requirement to use contraception after naxitamab

a. ALT and AST ≤5×ULN<br>b. Bilirubin ≤1.5×ULN<br><br>7. Acceptable kidney function defined as:<br>a. eGFR >60 mL/min/1.73 m^2 calculated by the 2009 revised Bedside Schwartz equation<br><br>8. Written informed consent from legal guardian(s) and/or patient in accordance with local regulations. Children must provide assent as required by local regulations	treatment: she must have undergone surgical sterilization (hysterectomy or bilateral ovariectomy)<br><br>9. Inability to comply with protocol requirements, including PK studies, as determined by the investigator<br><br>10. History of allergy or known hypersensitivity to GM-CSF, yeast-derived products, or any component of GM-CSF or naxitamab<br><br>11. History of anaphylactic reactions CTCAE Grade 4 related to prior GD2 antibody therapy<br><br>12. NB in CNS within 6 months of first dose of GM-CSF<br><br>13. Prior treatment with omburtamab (mu8H9) within 6 months of first dose of GM-CSF<br><br>14. Patients who have had allo-SCT or DLI. DLI or buffy coat infusion is defined as any kind of active allogenic lymphocyte suspension<br>a. Within 6 months of first dose of GM-CSF or<br>b. With a lymphocyte count <0.2×10^9/L<br><br>15. Patients who received HPC boost or top-up of allogenic stem cells (lymphocyte depleted) within 2 months of first dose GM-CSF<br><br>16. Any clinically meaningful abnormal finding in physical examination, vital signs, ECG, hematology, clinical chemistry, or urinalysis prior to inclusion into the trial, which in the opinion of the investigator may put

	the patient at risk because of his/her participation in the trial

Abbreviations: allo-SCT, allogeneic hematopoietic stem cell transplantation; ALT, alanine aminotransferase; ANC, absolute neutrophil count; AST, aspartate aminotransferase; BM, bone marrow; CNS, central nervous system; CTCAE, Common Terminology Criteria for Adverse Events; DLI, donor lymphocyte infusion; ECG, electrocardiogram; eGFR, estimated glomerular filtration rate; FDG-PET, fluorodeoxyglucose positron emission tomography; GM-CSF, granulocyte-macrophage colony-stimulating factor; HPC, hematopoietic progenitor cell; INRG, International Neuroblastoma Risk Group; MIBG, meta-iodobenzylguanidine; MR, minimal response; NB, neuroblastoma; PK, pharmacokinetic; PR, partial response; SD, stable disease; ULN, upper limit of normal.

Supplementary Table 8. Naxitamab plus GM-CSF treatment cycle in Trial 201, including timings of key premedications

	Infusion day											
	Day -4	Day -3	Day -2	Day -1	Day 0	Day 1	Day 2	Day 3	Day 4	Day 5	Day 6	Day 7
**Naxitamab IV**						3 mg/kg		3 mg/kg		3 mg/kg		
**GM-CSF SC**	250 µg/m²/day			500 µg/m²/day ≥1 hour preinfusion								
**Gabapentin po**	5–10 mg qd				5–10 mg bid				5–10 mg tid			
**Glucocorticosteroids iv**						2 mg/kg 30–120 min preinfusion[a]		PRN[a]		PRN[a]		
**Opioids po/IV**						45–60 min preinfusion; PRN during infusion		45–60 min preinfusion; PRN during infusion		45–60 min preinfusion; PRN during infusion		
**Antihistamine, H2 antagonist, acetaminophen, antiemetic iv**						30 min preinfusion		30 min preinfusion		30 min preinfusion		

Treatment cycles repeated Q4W (±1 week) until CR or PR, followed by 5 additional cycles Q4W (±1 week). Subsequent cycles repeated Q8W (±2 weeks) through 101 weeks from first infusion at investigator discretion. Three-year follow-up after end of trial.

[a] Glucocorticoids (methylprednisolone) must be given to all patients at the first infusion in the first cycle, but if Grade 3 bronchospasm or anaphylaxis are experienced, repeat glucocorticoid infusion, e.g., during infusions 2 and 3 within the same cycle, or infusion 1 of subsequent cycles; glucocorticoids can also be administered per treating physician's discretion when infusion-related AEs are not adequately controlled by other premedications.

Abbreviations: AE, adverse event; bid, twice daily; CR, complete response; GM-CSF, granulocyte-macrophage colony-stimulating factor; IV, intravenous; po, oral; PR, partial response; PRN, as needed; Q4W, once every 4 weeks; Q8W, once every 8 weeks; qd, once daily; SC, subcutaneous; tid, three times daily.

Supplementary Table 9. Definition of response in bone and BM components

**Tumor response at metastatic bone sites is assessed using anatomic and MIBG imaging (FDG-PET for MIBG non-avid tumors)**	**BM metastatic response is assessed using bilateral BM aspirates as well as BM biopsies (2+2 samples per INRC)**
• CR: MIBG uptake or FDG-PET uptake (for MIBG non-avid tumors) of non-primary lesions resolves completely<br><br>• PR: No new lesions AND<br>o ≥50% reduction in MIBG absolute bone score (relative MIBG bone score ≥0.1 to ≤0.5)[a]<br>OR<br>o ≥50% reduction in number of FDG-PET-avid bone lesions<br><br>• PD: Any of the following:<br>o Any new soft tissue lesion detected by CT/MRI that is also MIBG avid or FDG-PET avid<br>o Any new soft tissue lesion seen on anatomic imaging that is biopsied and confirmed to be NB or ganglioneuroblastoma<br>o Any new bone site that is MIBG avid<br>o A new bone site that is FDG-PET avid (for MIBG non-avid tumors) AND has CT/MRI findings consistent with tumor OR has been confirmed histologically to be NB or ganglioneuroblastoma<br>o Relative MIBG score ≥1.2[a]<br><br>• SD: Neither sufficient shrinkage for PR nor sufficient increase for PD of non-primary lesions	• CR: BM with no tumor infiltration on reassessment, independent of baseline tumor involvement<br><br>• PD: Any of the following:<br>o BM without tumor infiltration that becomes >5% tumor infiltration on reassessment<br>OR<br>o BM with tumor infiltration that increases by >2-fold and has >20% tumor infiltration on reassessment<br><br>• MD: Any of the following:<br>o BM with ≤5% tumor infiltration and remains >0 to ≤5% tumor infiltration on reassessment<br>OR<br>o BM with no tumor infiltration that has ≤5% tumor infiltration on reassessment<br>OR<br>o BM with >20% tumor infiltration that has >0 to ≤5% tumor infiltration on reassessment<br><br>• SD: BM with tumor infiltration that remains positive with >5% tumor infiltration on reassessment but

	does not meet CR, MD, or PD criteria

[a] Relative MIBG score (based on Curie score) is the absolute score for bone lesions at time of response assessment divided by the absolute score for bone lesions at baseline before therapeutic interventions.

Abbreviations: BM, bone marrow; CR, complete response; CT, computed tomography; FDG-PET, [^{18}F] fluorodeoxyglucose positron emission tomography; INRC, international neuroblastoma response criteria; MD, minimal disease; MIBG, meta-iodobenzylguanidine; MRI, magnetic resonance imaging; NB, neuroblastoma; PD, progressive disease; PR, partial response; SD, stable disease.

Supplementary Table 10. Determination of overall response

Response	Criterion
CR	All components meet criteria for CR
PR	PR in at least 1 component, and all other components are either CR, MD (bone marrow only), PR (soft tissue or bone), or NI[a]; no component with PD
MR	PR or CR in at least 1 component but at least 1 other component with SD; no component with PD
SD	SD in 1 component with no better than SD or NI[a] in any other component; no component with PD
PD	Any component with PD

[a]Site not involved at study entry and remains uninvolved.

Abbreviations: CR, complete response; MD, minimal disease; MR, minor response; NI, not involved; PD, progressive disease; PR, partial response; SD, stable disease.

Trial 201 Redacted Protocol and SAP

-mAbs Therapeutics A/S

CONFIDENTIAL

CLINICAL TRIAL PROTOCOL

A Pivotal Phase 2 Trial of Antibody Naxitamab (hu3F8) and Granulocyte-Macrophage Colony Stimulating Factor (GM-CSF) in High-Risk Neuroblastoma Patients with Primary Refractory Disease or Incomplete Response to Salvage Treatment in Bone and/or Bone Marrow

Phase 2, International Clinical Trial

Clinical Trial ID: 201

EudraCT No: 2017-001829-40

IND No: 132793

Version 12.0_14Jan2021

This trial protocol is the property of Y-mAbs Therapeutics A/S and is a confidential document. It is not to be copied or distributed to other parties without written approval from Y-mAbs Therapeutics A/S.

SIGNATURE PAGE

Sponsor Protocol Author		
Y-mAbs Therapeutics A/S<br>[redacted]<br>Director Clinical Operations	Please refer to e-signature page	
Print Name:	Signature:	Date:

Sponsor Medical Monitor/Expert		
Y-mAbs Therapeutics A/S<br>[redacted]<br>Medical Director	Please refer to e-signature page	
Print Name:	Signature:	Date:

Sponsor Biometrics		
Y-mAbs Therapeutics A/S<br>[redacted]<br>Senior Project Statistician	Please refer to e-signature page	
Print Name:	Signature:	Date:

TABLE OF CONTENTS

1 PROTOCOL SYNOPSIS 7

2 LIST OF ABBREVIATIONS AND DEFINITIONS OF TERMS 12

3 FLOW CHART 15

4 INTRODUCTION 17
4.1 Medical background 17
4.2 Drug profile 18
4.2.1 Rationale for naxitamab 18
4.2.2 Non-clinical data for naxitamab 18
4.2.3 Clinical status of naxitamab 18
4.2.4 Rationale for GM-CSF 19

5 RATIONALE AND OBJECTIVE 20
5.1 Rationale for performing the trial 20
5.2 Rationale for dose selection 20
5.2.1 Naxitamab 20
5.2.2 GM-CSF 20
5.3 Benefit – risk assessment 21
5.4 Trial objectives 21
5.4.1 Primary objective 21
5.4.2 Secondary objectives 21
5.4.3 Secondary objectives in long-term follow-up 21

6 DESCRIPTION OF DESIGN AND TRIAL POPULATION 22
6.1 Overall trial design and plan 22
6.1.1 Screening/pre-treatment evaluation 23
6.2 Data monitoring committee 23
6.3 Follow-up 23
6.4 Recruitment period 24
6.5 End of trial 24
6.6 Number of patients 24
6.7 Patient selection and withdrawal 25
6.7.1 Inclusion criteria 26
6.7.2 Exclusion criteria 26
6.7.3 Withdrawal criteria 27
6.7.4 Management of selected naxitamab Adverse Reactions 28
6.7.5 Management of GM-CSF (sargramostim) Adverse Reactions 33

7 TREATMENTS 34
7.1 Investigational medicinal products (IMPs) 34
7.1.1 Administration of IMP for each patient 34
7.1.2 Drug accountability and compliance check 35
7.1.3 Packaging and labelling 35
7.1.4 Storage and handling 35
7.1.5 Patient compliance 36
7.1.6 Overdose, medication errors, misuse and abuse 36
7.2 Concomitant therapy 36
7.2.1 Therapy allowed during trial 36
7.2.2 Pre-medications prior to naxitamab infusions 37
7.2.3 Prohibited therapy and procedures during the trial 39

7.3 Treatment after discontinuation of IMP 40

8 ENDPOINTS 41
8.1 Primary endpoint 41
8.2 Secondary endpoints 41
8.3 Secondary endpoints including long-term follow-up 41

9 PROCEDURES AND ASSESSMENTS 42
9.1 Efficacy assessments 42
9.1.1 Primary endpoint assessments 42
9.1.2 Secondary endpoint assessments 44
9.2 Other 46
9.2.1 Demographics 46
9.2.2 Physical examination 46
9.2.3 Height and body weight 46
9.2.4 Performance test 46
9.2.5 Non-child-bearing potential/serum pregnancy test 46
9.2.6 Research blood 47
9.2.7 Medical and surgical history 48
9.2.8 Vital signs & ECG 48
9.2.9 Hematology and clinical chemistry 49
9.2.10 Pain assessment 49
9.2.11 Quality of Life 49
9.2.12 Concomitant medication 50
9.2.13 Unscheduled visits 50

10 ADVERSE EVENTS 51
10.1 Definitions 51
10.1.1 Definition of Adverse Event 51
10.1.2 Definition of Serious Adverse Event 51
10.1.3 Definition of non-serious AE 51
10.1.4 Pre-defined AEs of Special Interest 52
10.2 Adverse Event Recording 52
10.2.1 Pre-existing conditions 52
10.2.2 Procedures 52
10.2.3 Signs and symptoms of the cancer disease 52
10.2.4 Pregnancy 52
10.2.5 Severity 52
10.2.6 Relationship to IMP 53
10.2.7 Outcome 53
10.3 Side-effects related to naxitamab and GM-CSF 54
10.4 Reporting of AEs 54
10.5 Follow-Up on AEs 55

11 LABORATORIES 57

12 STATISTICAL METHODS AND DETERMINATION OF SAMPLE SIZE 58
12.1 General overview 58
12.2 Populations of interest 58
12.3 Efficacy analysis 58
12.4 Safety analysis 59
12.5 PK analysis and analysis of ADA formation 59

12.6 Pharmacodynamic analysis 60
12.7 Quality of Life 60
12.8 Interim analysis 60
12.9 Subgroup analyses 60
12.10 Sample size 60
12.11 Handling of missing data 60
12.12 Reporting 61

13 ETHICS 62
13.1 Independent Ethics Committee (IEC) or Institutional Review Board (IRB) 62
13.2 Ethical conduct of the trial 62
13.3 Patient information and informed consent 62
13.4 Confidentiality 63

14 MONITORING AND QUALITY ASSURANCE 64
14.1 Compliance with Good Clinical Practice 64
14.1.1 Protocol compliance 64
14.1.2 Training of personnel 64
14.2 Monitoring 64
14.3 Source Data Verification 65
14.3.1 Source data 65
14.3.2 Direct access to source data/documents 65
14.3.3 Access to information for monitoring 65
14.3.4 Monitoring of pharmacies 66
14.3.5 Access to information for auditing or inspections 66
14.4 Quality Assurance 66

15 DATA HANDLING AND RECORD KEEPING 67
15.1 Electronic Case Report Forms 67
15.2 eCRF 67
15.3 Trial documents at site and record retention 67
15.4 Data management 68

16 REPORTING AND COMMUNICATION OF RESULTS 69
16.1 Publication 69
16.2 Use of information 69

17 INSURANCE 70

18 CHANGES AND DEVIATIONS TO THE PROTOCOL 71

19 PREMATURE TERMINATION OF THE TRIAL 72
19.1 Criteria for putting patient enrolment on hold 72
19.2 Criteria for termination of the trial 72

20 REFERENCES 73

TABLE OF TABLES

Table 1 Schedule of events: Screening, treatment cycles, EOT and long-term FU 15
Table 2 Efficacy results among non-PD patients in trial 12-230 19
Table 3 Expectations to response assessment after EOT 24
Table 4 IMP characteristics 34
Table 5 Tumor response at metastatic bone sites 42
Table 6 BM metastatic response 43
Table 7 Determination of objective response 43
Table 8 Timing of serum collection for PK and ADA at cycle 1 45
Table 9 Timing of serum collection for PK and ADA at cycle 2 45

TABLE OF FIGURES

Figure 1 One cycle of naxitamab + GM-CSF 22
Figure 2 Treatment and response assessment schedule 23
Figure 3 Hypertension algorithm for BP parameter >99th percentile by age 32

TABLE OF APPENDICES

Appendix 1 Contact list of Clinical Trial Team Members 77
Appendix 2 Creatinine-based 2009 revised Bedside Schwartz Equation 78
Appendix 3 Wong-Baker FACES® Pain Rating Scale 79
Appendix 4 FLACC Behavioral Scale 80

1 PROTOCOL SYNOPSIS

**TITLE**	A Pivotal Phase 2 Trial of Antibody Naxitamab (hu3F8) and Granulocyte-Macrophage Colony Stimulating Factor (GM-CSF) in High-Risk Neuroblastoma Patients with Primary Refractory Disease or Incomplete Response to Salvage Treatment in Bone and/or Bone Marrow
**EudraCT Number**	2017-001829-40
**Investigational New Drug (IND)**	132793
**Principal Investigator**	Trial Coordinating Investigator:<br>Brian H. Kushner, M.D.<br>Memorial Sloan Kettering Cancer Center<br>1275 York Ave.<br>New York, NY 10065
**Investigational sites**	Several sites in the US, EU and Asia
**Sponsor**	Y-mAbs Therapeutics A/S<br>Agern Allé 11<br>2970 Hoersholm<br>Denmark
**Trial ID**	201
**Trial Design**	Single-arm, open-label Phase 2 trial.
**Patient population**	The patient population for the trial comprises high-risk neuroblastoma (NB) patients with primary refractory disease or incomplete response to salvage treatment in bone and/or bone marrow (BM).<br><br>*Primary refractory disease*<br>Defined as no prior relapse or progressive disease (PD) but incomplete metastatic response (Stable Disease (SD), Minor Response (MR), Partial Response (PR) defined by INRC) to treatment in bone and/or BM as documented either by histologic evidence of NB in bone or BM and/or abnormal ^{123}I-metaiodobenzylguanidine (MIBG) uptake in skeletal sites.<br>In addition, at trial enrolment, patient cannot be more than 18 months from initiation of chemotherapy and must have received at least 4 cycles of standard induction chemotherapy for high-risk NB as defined in section 6.7 in the protocol.<br><br>*Incomplete response to salvage treatment (secondary refractory disease)*<br>Defined as relapse or PD and incomplete response (SD, MR, PR, but not PD defined by INRC) to salvage chemotherapy or MIBG therapy immediately before enrollment as defined in section 6.7 in the protocol.<br>Incomplete response to salvage treatment must be restricted to lesions in bone and/or BM as documented either by histologic evidence of NB in bone or BM and/or abnormal ^{123}I-MIBG uptake in skeletal sites. In addition, a minimum of two months from last MIBG/PET-CT documented relapse or PD prior to enrolment is required.<br><br>The population will fall into two distinct cohorts where cohort 1 will be the target population for efficacy as well as safety:<br><br>Cohort 1 include patients:<br>• Screened negative for ADA and<br>• With no pre-planned radiotherapy for metastatic lesions and<br>• Fulfilling the protocol for baseline disease defined as:<br>○ Evaluable disease at baseline in bone and/or bone marrow<br><br>Cohort 2 include patients:<br>• Screened positive for ADA or<br>• With pre-planned radiotherapy for metastatic lesions or<br>• Not fulfilling the protocol for baseline disease defined as:

	○ No evaluable disease at baseline in bone and/or bone marrow
**Objectives**	**Primary objective:**<br>1. To evaluate the centrally assessed objective response rate (ORR) to naxitamab + GM-CSF<br><br>**Secondary objectives:**<br>1. To evaluate the safety of naxitamab + GM-CSF<br>2. To evaluate Duration of Response (DoR) to naxitamab + GM-CSF<br>3. To evaluate the Complete Response (CR) rate with naxitamab + GM-CSF<br>4. To evaluate the investigator assessed ORR to naxitamab + GM-CSF<br>5. To evaluate the pharmacokinetics (PK) of naxitamab<br>6. To investigate the formation of ADAs<br>7. To evaluate the safety of naxitamab + GM-CSF in patients with positive ADA at trial entry<br>8. To evaluate Quality of Life (QoL)<br><br>**Secondary objectives in long-term follow-up:**<br>1. To evaluate Progression-Free Survival (PFS) with naxitamab + GM-CSF<br>2. To evaluate Overall Survival (OS) with naxitamab + GM-CSF
**Endpoints**	**Primary Endpoint**:<br>1. ORR during the naxitamab treatment period, centrally assessed according to the International Neuroblastoma Response Criteria (INRC)<br><br>**Secondary Endpoints:**<br>1. Safety will be evaluated by the incidence of adverse events (AEs) and serious adverse events (SAEs) graded according to Common Terminology Criteria for Adverse Events (CTCAE), version 4.0.<br>2. DoR, defined as the time from first objective response (CR or partial response [PR]) to PD; data will be censored at the date of last disease evaluation before new anti-NB treatment<br>3. CR rate, during the naxitamab treatment period, centrally assessed according to the INRC<br>4. ORR, during the naxitamab treatment period, investigator assessed according to the INRC<br>5. Assessment of the PK of naxitamab<br>6. Assessment of ADA formation<br>7. Intravenous (IV) opioid use during cycle 1 defined as total dosage of IV morphine (or equivalent opioid) administered 2 hours before infusion until 4 hours after end of infusion of naxitamab<br>8. IV opioid use for each cycle during the trial defined as total dosage of IV morphine (or equivalent opioid) administered 2 hours before infusion until 4 hours after end of infusion of naxitamab<br>9. Number of hospitalization days related to naxitamab during cycle 1, defined as number of overnight stays. Hospitalizations required solely for protocol-specified assessments (e.g., PK sampling) or non-medical circumstances are excluded<br>10. Number and percentage of infusion done in an outpatient setting<br>11. In patients with positive ADA at trial inclusion, safety will be evaluated by the incidence of AEs and SAEs graded according to CTCAE, version 4.0<br>12. Happiness and activity levels measured over time assessed by caretaker<br><br>**Secondary endpoints including long-term follow-up:**<br>1. PFS, defined as the time from the 1st infusion of naxitamab until PD or death, whichever comes first; data will be censored at the date of last disease evaluation before new anti-NB treatment<br>2. OS, defined as the time from the 1st infusion of naxitamab until death; data censored at last date known to be alive

Patient selection criteria

Inclusion criteria:

1. Documented diagnosis of NB as defined per INRC as
   a. histopathology of tumor biopsy, or
   b. BM aspirate or biopsy indicative of NB by histology, plus high blood or urine catecholamine metabolite levels or Myelocytomatosis Viral-Related Oncogene, Neuroblastoma derived (*MYCN)* amplification, or
   c. MIBG-avid lesion(s)
2. High-risk NB patients with either primary refractory disease or incomplete response to salvage treatment (in both cases including SD, MR and PR) evaluable in bone and/or BM as defined in section 6.7. If disease is only present in bone the patient must have evaluable disease outside the radiation areas for being eligible in the trial, please see section 7.2.1. If disease is only present in the BM the involvement must be >5%.
3. Life expectancy ≥6 months
4. Age ≥12 months
5. Acceptable hematological status at screening, (hematological support is allowed if administered ≥1 week before first screening procedure), defined as:
   a. Hemoglobin ≥8 g/dL (5.0 mmol/L)
   b. White blood cell count ≥1000/µL (1.0 x10^9/L)
   c. Absolute neutrophil count (ANC) ≥500/µL (0.5 x10^9/L)
   d. Platelet count ≥25,000/µL (25 x10^9/L)
6. Acceptable liver function defined as:
   a. Alanine aminotransferase (ALT) and aspartate aminotransferase (AST) ≤5 times upper limit of normal (ULN)
   b. Bilirubin ≤1.5 x ULN
7. Acceptable kidney function defined as:
   a. Estimated Glomerular Filtration Rate (eGFR) >60 mL/min/1.73 m^2 calculated by the 2009 revised Bedside Schwartz Equation (Appendix 2)
8. Written informed consent from legal guardian(s) and/or patient in accordance with local regulations. Children must provide assent as required by local regulations.

Exclusion criteria:

1. Any systemic anti-cancer therapy, including chemotherapy or immunotherapy, within 3 weeks of 1st dose of GM-CSF
2. Evaluable NB outside bone and BM defined as follows:
   - MIBG-avid tumor: Definite MIBG uptake in tumor tissues outside bone and BM
   - MIBG nonavid tumor: Definite uptake in tumor tissues outside bone and BM on FDG-PET
3. Actively progressing disease at trial entry according to Park criteria (Park et al. 2017) (see section 6.7)
4. Existing major organ dysfunction CTCAE >Grade 2, with the exception of hearing loss, hematological status, kidney and liver function.
5. Active life-threatening infection
6. Prior treatment with naxitamab
7. Karnofsky/Lansky score <50%
8. Pregnancy or a woman who is breast-feeding (women of child-bearing potential must have a negative pregnancy test at screening). A woman of child-bearing potential is excluded if she does not agree to use highly effective contraception for a period of 40 days after the last naxitamab infusion according to section 9.2.5. A sterilized or infertile woman is exempt from the requirement to use contraception after naxitamab treatment: she must have undergone surgical sterilization (hysterectomy, or bilateral ovariectomy).
9. Inability to comply with protocol requirements, including PK studies, as determined by the investigator

	10. History of allergy or known hypersensitivity to GM-CSF, yeast-derived products, or any component of GM-CSF or naxitamab<br>11. History of anaphylactic reactions CTCAE grade 4 related to prior GD2 antibody therapy<br>12. NB in central nervous system (CNS) within 6 months of 1st dose of GM-CSF<br>13. Prior treatment with omburtamab (mu8H9) within 6 months of 1st dose of GM-CSF<br>14. Patients who have had allogeneic hematopoietic stem cell transplantation (allo-SCT) or donor-lymphocyte-infusion (DLI). DLI or buffy coat infusion is defined as any kind of active allogenic lymphocyte suspension<br>a. within 6 months of 1st dose of GM-CSF or<br>b. with a lymphocyte count < 0.2 x10^9/L<br>15. Patients who received Hematopoietic Progenitor Cell (HPC) boost or "top-up" of allogenic stem cells (lymphocyte-depleted) within 2 months of 1st dose GM-CSF.<br>16. Any clinically meaningful abnormal finding in physical examination, vital signs, ECG, hematology, clinical chemistry, or urinalysis prior to inclusion into the trial, which in the opinion of the investigator, may put the subject at risk because of his/her participation in the study.
**Methodology**	The safety and efficacy of naxitamab and GM-CSF is assessed using a single-arm (uncontrolled) trial design in patients with high-risk NB in bone and/or BM being primary refractory or having incomplete response to salvage treatment. All patients will at the investigator's discretion receive treatment for up to 101 weeks following the first naxitamab infusion. After end of treatment (EOT), each patient will have long-term follow-up (FU) for 3 years.
**Number of patients (planned)**	Trial 201 plans to enroll 37 patients evaluable for objective response and screened negative for ADA and with no pre-planned radiotherapy for metastatic lesions and fulfilling the protocol for baseline disease assessed by independent review (cohort 1). During discussion with regulatory authorities further knowledge on PFS has been requested, which is why it has been decided to enroll additional patients, targeting a total of at least 85 patients in cohort 1. The enrollment will be stopped when the 85 eligible cohort 1 patients have been enrolled. The number of patients in cohort 2 is expected to be a maximum of 10 patients.
**Investigational Medicinal Product (IMP)**	The humanized immunoglobulin isotype G (IgG1) monoclonal antibody (mAb) naxitamab targets GD2 (disialoganglioside). Route of administration is intravenous infusion.<br><br>Sargramostim (yeast-derived human recombinant GM-CSF) is available as a 250 µg single-use vial of lyophilized powder. Route of administration is subcutaneous injection.
**Trial period and duration**	Patients receive treatment for up to 101 weeks following the 1st naxitamab infusion. After EOT, patients have long-term FU for 3 years.<br><br>Each cycle starts with 5 days (days -4 to 0) of GM-CSF administered at 250 µg/m²/day (i.e., before the days of naxitamab infusions). GM-CSF is thereafter administered at 500 µg/m²/day on days 1 to 5. The naxitamab is administered at 3 mg/kg/day on days 1, 3, and 5 (i.e., 3 doses with a total of 9 mg/kg per cycle).<br><br>Treatment cycles are repeated every 4 weeks (±1 week) until CR or PR followed by 5 additional cycles every 4 weeks (±1 week). Subsequent cycles are repeated every 8 weeks (±2 weeks) through 101 weeks from first infusion at the discretion of the investigator.

**Statistical considerations**	For the primary efficacy endpoint of ORR, 37 patients are sufficient to show a statistically significantly greater response than 20%, with a 90% power, and 95% confidence interval, using the Clopper-Pearson exact methodology, and assuming an actual response rate ≥45%. The endpoint will be calculated using the full analysis set (FAS). Effectiveness will be concluded if the lower limit of the 95% confidence interval exceeds 20%. PFS and OS will be considered secondary endpoints for long-term follow-up. Assuming a 3-year PFS of 23% a sample size of 80 patients will give approximately 80% power to show that the lower bound of the two-sided 95% confidence interval of PFS at 3-years is above 12%. Time to event endpoints will be analyzed using Kaplan-Meier methods. Standard safety summaries will be provided. The incidence of AEs will be tabulated and reviewed for potential significance and clinical importance.

2 LIST OF ABBREVIATIONS AND DEFINITIONS OF TERMS

ACCC	American College of Critical Care Medicine
ACE	Angiotensin-Converting Enzyme
ADA	Anti-drug Antibody
ADCC	Antibody-Dependent Cellular Cytotoxicity
ADR	Adverse Drug Reaction
AE	Adverse Event
Allo-SCT	Allogeneic Hematopoietic Stem Cell Transplantation
ALT	Alanine Aminotransferase
ANC	Absolute Neutrophil Count
AST	Aspartate Aminotransferase
ATC	Around the clock
AUC	Area Under the Curve
BID	Two times per day
BM	Bone Marrow
BP	Blood Pressure
BSA	Body Surface Area
BUN	Blood Urea Nitrogen
C	Cycle
CBC	Complete Blood Count
CDC	Complement-Dependent Cytotoxicity
CHO	Chinese Hamster Ovary
CLAE	Clinical Laboratory Adverse Event
Cmax	Maximum serum concentration
Cmin	Minimum serum concentration
CNS	Central Nervous System
COG	Childrens Oncology Group
CR	Complete Response
CRA	Clinical Research Associate
CRF	Case Report Form
CRO	Contract Research Organization
CT	Computed Tomography
CTCAE	Common Terminology Criteria for Adverse Events
dL	Deciliter
DLI	Donor-lymphocyte-infusion
DLT	Dose-Limiting Toxicity
DMC	Data Monitoring Committee
DNA	Deoxyribonucleic Acid
DoR	Duration of Response
ECG	Electrocardiogram
eCRF	electronic Case Report Form
EFS	Event-Free Survival
eGFR	estimated Glomerular Filtration Rate
EMA	European Medicines Agency
EOT	End of Treatment
EU	European Union
FAS	Full Analysis Set
Fc	Fragment Crystallizable
FDG-PET	Fluorodeoxyglucose Positron Emission Tomography
FLACC	Face, Legs, Arms, Cry, Consolability
FSH	Follicle-Stimulating Hormone
FU	Follow-Up
g	Gram(s)
GCP	Good Clinical Practice
GD2	Disialoganglioside
GM-CSF	Sargramostim - Granulocyte-Macrophage Colony Stimulating Factor
GMP	Good Manufacturing Practice

h	Hour(s)
HACA	Human Anti-Chimeric Antibody
HAMA	Human Anti-Mouse Antibody
Hct	Hematocrit
HgB	Hemoglobin
HPC	Hematopoietic Progenitor Cell
Hu3F8	Naxitamab (drug compound used in this trial is naxitamab, however the drug compound has also been described as hu3F8, hence both names will occur in documents for naxitamab)
HUS	Hemolytic Uremic Syndrome
IB	Investigator's Brochure
ICF	Informed Consent Form
ICH	International Conference on Harmonization
IEC	Independent Ethics Committee
IgG	Immunoglobulin isotype G
IM	Intramuscular
IMP	Investigational Medicinal Product
IND	Investigational New Drug
INRC	International Neuroblastoma Response Criteria
INRG	International Neuroblastoma Risk Group
IRB	Institutional Review Board
IU	International unit
IV	Intravenous
kg	Kilogram(s)
L	Liter
LDH	Lactate Dehydrogenase
mAb	Monoclonal Antibody
mcg	Microgram
MCH	Mean Corpuscular Hemoglobin
MCHC	Mean Corpuscular Hemoglobin Concentration
MCV	Mean Corpuscular Volume
MD	Minimal Disease
MedDRA	Medical Dictionary for Regulatory Activities
mg	Milligram(s)
MIBG	Metaiodobenzylguanidine
MIBG-SPECT/CT	Metaiodobenzylguanidine Single-photon emission computed tomography/ computed tomography
min	Minute(s)
mL	Milliliter(s)
mmol	Millimol
MR	Minor Response
MRI	Magnetic Resonance Imaging
MSK	Memorial Sloan Kettering Cancer Center
MYCN	Myelocytomatosis Viral-Related Oncogene, Neuroblastoma derived
NB	Neuroblastoma
NCI	National Cancer Institute
NIH	National Institutes of Health, US
ORR	Objective Response Rate
OS	Overall Survival
PD	Progressive Disease
PFS	Progression-Free Survival
pH	Numeric scale used to specify the acidity or basicity of an aqueous solution
PI	Principal Investigator
PK	Pharmacokinetic
PO	Peroral
PPAS	Per protocol analysis set
PR	Partial Response
PRES	Posterior reversible encephalopathy syndrome
PPI	Proton-pump inhibitors

PRN	When necessary (pro re nata)
QoL	Quality of life
RBC	Red blood cells
SAE	Serious Adverse Event
SAF	Safety Analysis Set
SAP	Statistical Analysis Plan
SAS	Statistical Analysis Software
SD	Stable Disease
SoC	Standard of Care
SOP	Standard Operating Procedure
SpO2	Peripheral oxygen saturation
SUSAR	Suspected Unexpected Serious Adverse Reaction
t½	Terminal Half-Life
TEAE	Treatment-Emergent Adverse Event
ULN	Upper Limit of Normal
US	United States
WBC	White blood cells
WOCBP	Woman of childbearing potential
yr	Year

3 FLOW CHART

Table 1 Schedule of events: Screening, treatment cycles, EOT and long-term FU

Treatment/Measurements/Evaluations	Screening	First naxitamab treatment cycle									
	Day -25 to -4	Day -4 to 0	Day 1	Day 2	Day 3	Day 4	Day 5	Day 8, 12, 17, 24[13]	Next cycles[18]	EOT[19]	Long-term FU[21]
Informed consent	X										
Eligibility check[1]	X										
Demographics	X										
Physical examination[2]	X								X	X	
Height and weight[3]	X		X						X		
Performance test[4]	X								X	X	
Serum pregnancy test, if applicable	X								X		
Research blood[5]	X										
Bone marrow biopsy/aspirate[6]	X[24]								X	X[20]	X[21]
Imaging[7]	X[24]								X	X	X[21]
Complete medical and disease history	X										X[22]
Vital signs[8]	X		X		X		X		X	X	
ECG[9]	X								X	X	
Clinical chemistries[10]	X		X		X		X		X		
Hematology[11]	X	X	X		X		X		X		
Blood for PK[12]			X	X	X		X	X	X	X	
Blood for ADA[12]			X					X	X	X	
Subcutaneous GM-CSF (250 µg/m^2/day)[13]		X							X		
Subcutaneous GM-CSF (500 µg/m^2/day)[13]			X	X	X	X	X		X		
Pre-medication (analgesics and/or antihistamines)[14]		X	X	X	X	X	X		X		
Intravenous naxitamab (3 mg/kg/day)[15]			X		X		X		X		
Pain assessment[16]			X		X		X		X		
Adverse events[17]	X	X	X	X	X	X	X	X	X	X	X[21]
Concomitant medications	X	X	X	X	X	X	X	X	X	X	X[23]
QoL[25]		X[26]	X	X	X	X	X	X	X[25,26]		

[1] Extent of disease evaluation can be performed as early as Day -25, provided that patient has not received any treatment after the evaluation.

[2] A physical examination should be performed at screening, prior to first infusion of naxitamab in each cycle and otherwise as clinically indicated.

[3] Measured for calculation of IMP doses: GM-CSF doses according to last measured weight/height captured in the eCRF. Naxitamab doses according to weight at first day of each cycle. If it is necessary to order the naxitamab infusion at the pharmacy before the patient is available for weighing on the first day of infusion, the dose can be calculated from the last measured weight captured in the eCRF. However, the weight must be checked at the first day of infusion and if the patient has lost ≥5% in weight, the infusion must be reduced accordingly. The following naxitamab infusions within the cycle must be adjusted to the current weight if this has changed ≥5% in any direction.

[4] Lansky Scale <16 years, Karnofsky Scale ≥16 years. Should be assessed before cycles 1, 4, 7, 10, 13 and at EOT.

[5] Heparinized blood used to test genomic Deoxyribonucleic Acid (DNA) for FcgRIIa (CD32a) and FcgRIIIa (CD16a) gene polymorphism.

[6] Bilateral aspirates and biopsies (2+2), performed week 6 (post cycle 2 (C2)).
- For patients with CR or PR at week 6 (post C2): repeat sampling weeks 18 (+1 week), 30 (+1 week), 46 (±1 week), 62 (±1 week) and 78 (±1 week). The repeat sampling planned at week 18 and 30 has to be scheduled with a minimum of 12 weeks since last sampling.
- For patients with MR or SD at week 6 (post C2): repeat sampling at weeks 14 (+1 week), 26 (+1 week), 38 (+1 week), 54 (±1 week), and 78 (±1 week). The repeat sampling planned at week 26 and 38 has to be scheduled with a minimum of 12 weeks since last sampling.

[7] Including computed tomography (CT) or magnetic resonance imaging (MRI) and MIBG scan or ^{18}F-Fluorodeoxyglucose Positron Emission Tomography (FDG PET (if not avid on MIBG). Alternatively, by MIBG-SPECT/CT. Schedule for imaging follows timing of BM biopsies (see #6 above). Per imaging charter, serum glucose determination must be performed prior to the administration of FDG (PET scan only). Please refer to the charter for more details.

[8] Measurements of heart rate, respiratory rate, temperature, and blood pressure continuously in connection with each treatment cycle as outlined in section 9.2.8. Furthermore, vital signs should be captured at screening and EOT.

[9] Local assessment of electrocardiogram (ECG) during screening, before cycle 2 and 4 and at end of treatment. If the patient needs sedation before ECG, the ECG can be performed in connection with imaging, hence if no imaging is planned for cycle 4, the ECG can be postponed until next imaging.

[10] Liver and renal function tests and serum electrolytes prior to each naxitamab infusion, please refer to section 9.2.9.

[11] Complete blood count (CBC) to be drawn before each naxitamab infusion according to section 9.2.9. CBC must be checked before the first dose of GM-CSF (administered on Day -4) and before administration of GM-CSF on Day 1, 3 and 5. If Absolute Neutrophil Count (ANC) > 20 x10^9/L or WBC > 50 x10^9/L, GM-CSF should not be administered until values fall below the above-mentioned thresholds. CBC is repeated on sequential days if ANC > 20 x10^9/L or WBC > 50 x10^9/L.

[12] Blood for PK and ADA is collected within the time points as outlined in section 9.1.2.5.

[13] Cycles follow the daily schedule shown in Figure 1.

[14] Premedication must be administered prior to naxitamab infusion according to section 7.2.2.

[15] Naxitamab infusion is started ≥1 hour after subcutaneous injection with GM-CSF.

[16] The same pain scale must be used during all naxitamab infusions. Please refer to section 9.2.10 for more information.

[17] Adverse events and concomitant medications are assessed at each clinic visit. The standard AE collection stops at the EOT visit.

[18] Treatment cycles are repeated every 4 weeks (±1 week) until CR or PR followed by 5 additional cycles every 4 weeks (±1 week). Subsequent cycles are repeated every 8 weeks (±2 weeks) until EOT.

[19] EOT measurements/evaluations should take place minimum 6 weeks (acceptable range 6-10 weeks) after last infusion. If the patient is withdrawn from the trial and is planned for new anti-NB treatment or investigator judges that the patient will not to be able to comply with the protocol the investigator should ensure that the patient, whenever possible, is examined according to the EOT procedures (see section 6.3).

[20] Not to be performed at EOT if the patient is withdrawn due to PD. However, PD should be documented (e.g. scan or biopsy performed for diagnosis).

[21] Long-term FU evaluations should take place approximately quarterly until 3 years after EOT and can be done locally at the referral site. Only SAEs considered related to the trial medication or new onset of cancers regardless of causality should be reported. The investigator should report these SAEs within the same timelines as for SAEs during the trial. The investigator will also monitor and document treatments and assessments according to clinical practice. After EOT, patients must have response assessment performed according to section 6.3.

[22] During the long-term FU, information on OS and PD will be collected.

[23] During the long-term FU, only therapy related to cancer will be collected.

[24] Screening imaging and BM examinations must be obtained at least 10 calendar days after end of any prior anti-cancer treatment.

[25] QoL to be assessed each evening from C1, day-4 to C2, day 7. The parents will answer two questions using a VAS in a diary. See section 9.2.11.

[26] QoL diary to be handed out at C1, day-4 and returned to the site at day of imaging/biopsy in week 6.

4 INTRODUCTION

4.1 Medical background

Disease background

Neuroblastoma (NB) is a rare cancer but is the most common extracranial solid tumor of childhood. More than 50 percent of patients have metastatic disease (stage 4) and typically in the BM (Maris et al. 2007; Mueller and Matthay 2009). Intensive induction chemotherapy, aggressive surgery, and radiotherapy have improved remission rates of high-risk NB in young patients (Pearson et al. 1992; LaQuaglia MP et al. 2004; Kushner BH et al. 2004). Treatments have been less successful in adolescents and adults, where NB is typically chemoresistant (Franks et al. 1997; Kushner et al. 2003; Maris 2010). Despite advances in frontline multimodal therapy, ~50% of high-risk NB patients have persistence of disease or develop progressive disease (PD). BM invasion is associated with a very poor prognosis (Basta et al. 2016; Morgenstern et al. 2016). After relapse, these patients have poor outcomes with 5-year OS of ~10% (London et al. 2011). For both primary refractory patients and patients having incomplete response to salvage treatment, salvage chemotherapy regimens using 1-3 agents have shown disappointing ORR of 10-35% (London et al. 2011; Bagatell et al. 2011; Di Giannatale et al. 2014; Moreno et al. 2017; Modak et al. 2017), and the same holds for salvage with ^{131}I-MIBG therapy (Wilson et al. 2014). Median PFS from trial entry for all, primary refractory, and relapsed patients in a meta-analysis of ITCC/SIOPEN European Phase 2 trials were 6.4, 12.5, and 5.7 months, respectively. Median OS from trial entry for all, primary refractory, and relapsed patients was 16, 28, and 11 months (Moreno et al. 2017). A 6% (+/- 2) PFS at 4-years in high-risk relapsed or refractory patients, was reported in a COG meta-analysis covering trials from 2002 until 2014 (London et al. 2017). Thus, there is a medical need for better treatment of high-risk NB being primary refractory or having incomplete response to salvage treatment.

Rationale for monoclonal antibody therapy for NB

Monoclonal antibody (mAb) therapy is an accepted treatment modality for cancer. Regulatory agencies in Europe and US have approved various mAbs for treatment of solid tumors and hematological malignancies. This modality, however, has remained inadequately exploited for treatment of pediatric cancer. Unlike chemotherapy or radiotherapy, administration of mAb is not myelosuppressive or genotoxic, and it generally comes with little long-term toxicity. These are critical considerations for young children. More importantly, mAbs are effective against metastatic cancer in well-vascularized sites of metastases, including BM infiltrated by NB cells. Furthermore, the PK and toxicities of human or humanized IgG1 mAbs have been extensively documented.

Several factors make NB well suited for mAb targeting. First, mAb mediates highly efficient antibody-dependent cellular cytotoxicity (ADCC) in the presence of human white blood cells. Second, mAb induces complement-dependent mediated cytotoxicity (CDC) of NB cells which lack decay accelerating factor CD55 and homologous restriction factor CD59 (Chen et al. 2000). Third, if colony-stimulating factors are administered, deposition of complement on NB cells enhances ADCC through activation of the iC3b receptor on neutrophils (Kushner and Cheung 1992; Metelitsa et al. 2002) which are not ablated by dose-intensive chemotherapy and recover early after myeloablative therapy plus stem cell transplantation (Mackall 2000). Fourth, intensive chemotherapy (standard of care for NB) designed to achieve clinical remission causes prolonged T-lymphopenia and immunosuppression (Mackall et al. 2000) in recipients who then are less likely to reject even murine mAbs (Kushner et al. 2007).

GD2 as a target in high-risk NB

GD2 is an adhesion molecule abundantly expressed in NB (Modak and Cheung 2007). By contrast, GD2 is rarely expressed in normal tissues, except for neurons, skin cells and pain fibers. This genetically stable antigen is rarely lost and relatively inert on the cell membrane. Scintigraphy, using radiolabeled mAb, confirms excellent targeting to tumors (Modak and Cheung 2007). At least two anti-GD2 mAb families (3F8 and 14.18) have been assessed clinically (Mujoo et al. 1989). Chimeric 14.18 (ch14.18) was recently approved as maintenance therapy for high-risk NB patients treated with autologous stem cell transplantation. Murine 3F8 (mu3F8), a specific anti-GD2 IgG3 kappa light chain murine immunoglobulin, induces cell death and mediates efficient ADCC and CDC *in vitro* and anti-NB activity *in vivo*. Over the past three decades, mu3F8 has been administered to >1,000 patients. Mu3F8 has shown therapeutic benefit in patients with metastatic NB when administered as a monotherapy or with GM-CSF in settings of refractory and relapsed disease as well as minimal residual disease (Cheung et al. 1987; Cheung et al. 2014; Cheung et al. 2012).

4.2 Drug profile

4.2.1 Rationale for naxitamab

The favorable clinical effect of mu3F8 against NB would not be possible if mu3F8 could not be given as multiple treatment cycles over months to years. Rapid production of human anti-mouse antibody (HAMA) can delay treatment, prevent further cycles, and compromise anti-tumor effects of mAb treatment. Reduction or elimination of mouse epitopes should reduce HAMA response, but even chimeric mAbs can induce human anti-chimeric antibody (HACA) unless the patient is immunocompromised (Albert et al. 2008). An obvious strategy to overcome these shortcomings is to humanize 3F8 (hu3F8, naxitamab).

4.2.2 Non-clinical data for naxitamab

Naxitamab was made using standard recombinant methods by transfection into Chinese Hamster Ovary (CHO) cells and selected for high IgG expression. In vitro studies indicate that ADCC with naxitamab exceeds that of mu3F8 (by 10- to >100-fold based on EC50), while CDC was slightly less intense. This superiority was consistently observed in ADCC assays, irrespective of the choice of donor and killer cells (see the Investigators Brochure (IB)). This large improvement in ADCC is most desirable given evidence for its role in the anti-tumor effects of mAbs in patients. CDC is effective against human NB cells (Saarinen et al. 1985) because of their low expression of CD55 (Cheung et al. 1988) and CD59 (Chen et al. 2000).

4.2.3 Clinical status of naxitamab

In five clinical trials at Memorial Sloan Kettering Cancer Center (MSK), naxitamab has been administered to >180 human outpatients in since 2011 and clinical details are available from all 57 patients in the Phase 1 part of the 12-230 trial (Cheung et al. 2017).

The 57 patients enrolled from 2012 to 2016 in the Phase 1 part were 0.6-9.0 (median 3.1) years from diagnosis, 0.9-24.3 (median 4.0) years old at diagnosis, and 2.4-31.3 (median 6.8) years old at start of naxitamab+GM-CSF immunotherapy. All had stage 4 NB and 12 (24%) of 49 tested had *MYCN*-amplified disease.

All 57 patients were heavily prior-treated, including with autologous stem-cell transplantation (n=24) and ^{131}I-MIBG therapy (n=17). Treatment <2 months before trial entry included

relatively non-immunosuppressive therapy in 50 patients (irinotecan-temozolomide in 21 patients, low-dose ifosfamide-carboplatin-etoposide in 15 patients, investigative agents in six patients, low-dose cyclophosphamide-topotecan in five, and ^{131}I-MIBG therapy in three patients), and strongly immunosuppressive chemotherapy (high-dose alkylators) in seven patients. Prior treatment also included anti-GD2 mAbs in 47 patients of whom 37 had prior exposure to mu3F8 and 16 had prior exposure to dinutuximab. Ten patients had previously been exposed to >1 anti-GD2 mAb.

After having excluded two patients with early dose-limiting toxicity (DLT) on the first day of treatment, 55 of 57 patients were included in the analysis of efficacy. Patients with PD at trial entry had no benefit. In contrast, an analysis of the 23 non-PD patients (with evaluable disease) which constitutes the patient population intended for future naxitamab therapy, showed evidence of anti-NB activity in [redacted] patients.

A sub-analysis of non-PD refractory disease by primary refractory disease and incomplete response to salvage treatment (secondary refractory disease) was performed and showed an encouraging ORR of [redacted] in the primary refractory population and ORR of [redacted] in the secondary refractory population (Table 2).

Among 25 patients with ‘no evaluable disease’, it was not possible to classify response by INRC. These patients, who were status-post 1-5 (median 2) prior relapses and therefore had a dismal prognosis, showed an encouraging 2-year event-free survival (EFS) of [redacted].

Table 2 Efficacy results among non-PD patients in trial 12-230

Patient group	CR/PR	SD	PD	2-year l EFS[1]
Primary refractory (n = 11)	[redacted]	[redacted]	[redacted]	-
Secondary refractory (n = 12)	[redacted]	[redacted]	[redacted]	-
No evaluable disease (n = 25)	-	-	-	[redacted]
All patients with evaluable disease (n = 23)	[redacted]	[redacted]	[redacted]	-

[1] EFS is assessed from the first infusion of naxitamab.
CR=complete remission/response; EFS=Event-free survival; PD=progressive disease; PR= partial response; SD= stable disease

These results are encouraging in the light of the poor results with existing treatment regimens, and they support the rationale for development of naxitamab for high-risk NB.

4.2.4 Rationale for GM-CSF

GM-CSF can amplify 3F8 anti-NB activity in patients via effects on granulocytes and macrophages (Cheung, Hsu, and Cheung 2012). Other reasons for combining GM-CSF with naxitamab include: (1) granulocyte production is only transiently suppressed by chemotherapy, and GM-CSF increases neutrophil and eosinophil production and is well tolerated compared to other cytokines such as interleukin-2 (Yu et al. 2010; Petrella et al. 2007), and (2) GM-CSF can activate monocytes-macrophages to phagocytose NB cells, and enhances the proliferation, maturation, and function of antigen-presenting cells that promote host anti-tumor response. Myeloid activation markers on granulocytes (CD11a, CD63, CD87, CD11b and the activation epitope CBRM1/5) were increased following GM-CSF therapy among NB patients.

5 RATIONALE AND OBJECTIVE

5.1 Rationale for performing the trial

Despite intensive frontline multimodal therapy, at least 50% of high-risk NB patients have persistence of disease or develop PD. After relapse, these patients have poor outcomes with 5-year OS of ~10% (London et al. 2011). For both primary refractory patients and patients having incomplete response to salvage treatment, salvage chemotherapy regimens using 1-3 agents have shown disappointing ORR of 10-35% (London et al. 2011; Bagatell et al. 2011; Di Giannatale et al. 2014; Moreno et al. 2017; Modak et al. 2017), and the same holds for salvage with ^{131}I-MIBG therapy (Wilson et al. 2014). Promising clinical results in trial 12-230 were achieved in patients with primary refractory disease and patients with incomplete response to salvage treatment in bone and/or BM (secondary refractory disease).

Various strategies have been applied to induce or augment immune-mediated attack against cancer. However, few clinical trials have successfully used mAb-mediated immunotherapy against solid tumors in children or adults, especially with ADCC as the key underlying immune mechanism. Building on the favorable results of mu3F8, naxitamab has several potential advantages over mu3F8 and 14.18 which include: (1) low immunogenicity allowing repeat treatments over years, (2) greatly improved ADCC potency, (3) longer serum half-life reducing the necessity of daily injections, (4) substantial reduction of pain side effects. This pivotal Phase 2 trial builds on the highly encouraging recent findings from the Phase 1 and 1/2 trials as regards major anti-NB activity of naxitamab + GM-CSF.

5.2 Rationale for dose selection

5.2.1 Naxitamab

Clinical trials with naxitamab have shown it to be safe across a large dose range from <1 mg/kg to 10 mg/kg per cycle. Neither DLTs nor Grade 3-4 toxicities were dose-related. No maximum tolerated dose was identified during the Phase 1 portion of trial 12-230, and dose escalation stopped at 9.6 mg/kg per cycle. Dose escalation was stopped on the basis of PK data, the investigator's assessment that 9.0 mg/kg per cycle provided the best balance between manageable pain for the patients, and clinical effectiveness of naxitamab. In addition, 9.6 mg/kg per cycle exceeded the dose level of chimeric 14.18 (ch14.18) and mu3F8 by more than 2.5-fold. Ch14.18 is approved as dinutuximab (Unituxin®), and mu3F8 is an investigational product previously used at MSK. Thus, a dose of 9 mg/kg per cycle was chosen for this Phase 2 trial. The distribution of three doses administered over five days (days 1, 3, and 5) was found to be safe, effective, and feasible as an outpatient treatment across several trials with naxitamab.

5.2.2 GM-CSF

Myeloid activation markers on granulocytes were increased following GM-CSF therapy among patients with metastatic NB. The favorable results of mu3F8 + GM-CSF in high-risk NB provide a strong rationale for combining naxitamab with GM-CSF. The doses of GM-CSF and the regimen used has proven effective and safe in a multitude of prior trials with murine and humanized 3F8 as well as other anti-cancer regimen immune-stimulatory agents (Hoeller et al. 2016).

5.3 Benefit - risk assessment

The presented clinical evidence demonstrates treatment effects on clinically relevant endpoints. High-risk NB with primary refractory disease or having incomplete response to salvage treatment has an unfavorable response to treatment with reported ORR of 30% and 15% to standard chemotherapy. The data with naxitamab + GM-CSF obtained with protocol 12-230 showed ORR of 73% and 42% in similar populations, respectively. The preliminary results show that naxitamab + GM-CSF substantially improved the outcome of high-risk NB. The sponsor therefore foresees that naxitamab will be of notable benefit to high-risk NB patients with primary refractory disease and patients with incomplete response to salvage treatment.

5.4 Trial objectives

5.4.1 Primary objective

1. To evaluate the centrally assessed ORR to naxitamab + GM-CSF

5.4.2 Secondary objectives

1. To evaluate the safety of naxitamab + GM-CSF
2. To evaluate DoR to naxitamab + GM-CSF
3. To evaluate the CR rate with naxitamab + GM-CSF
4. To evaluate the investigator assessed ORR to naxitamab + GM-CSF
5. To evaluate the PK of naxitamab
6. To investigate the formation of ADAs
7. To evaluate the safety of naxitamab + GM-CSF in patients with positive ADA at trial entry
8. To evaluate QoL

5.4.3 Secondary objectives in long-term follow-up

1. To evaluate PFS with naxitamab + GM-CSF
2. To evaluate OS with naxitamab + GM-CSF

6 DESCRIPTION OF DESIGN AND TRIAL POPULATION

6.1 Overall trial design and plan

It is planned that each patient will receive IMP for a maximum of 101 weeks after the first naxitamab infusion. After EOT, each patient will enter a long-term FU for 3 years where they will be monitored at the referral site.

Each investigational cycle is started with five days (days -4 to 0) of GM-CSF administered at 250 µg/m^2/day in advance of the start of naxitamab infusion. GM-CSF is thereafter administered at 500 µg/m^2/day on days 1 to 5. Naxitamab is infused at 3 mg/kg/day on days 1, 3, and 5 for a total dose of 9 mg/kg per cycle as shown in Figure 1. Please refer to section 7.1.1 for more information.

Figure 1 One cycle of naxitamab + GM-CSF

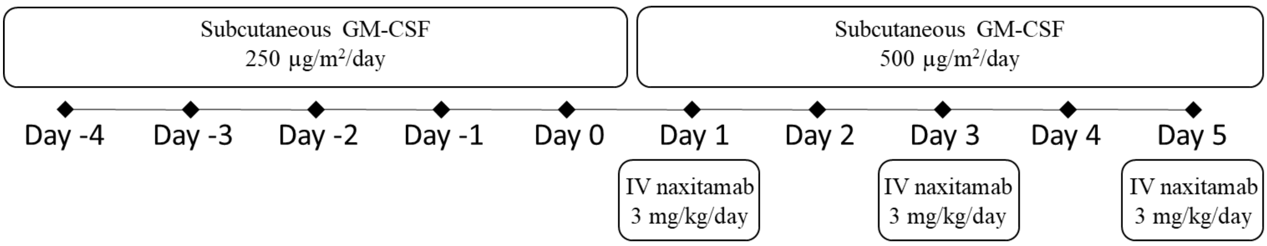

The treatment and evaluation schedule may require minor adjustment as clinically indicated (e.g., significant AEs) or due to non-medical circumstances (e.g., public holidays). Patients can complete the missed day of naxitamab the following week, but the 3 infusions of naxitamab must be administered within a period of maximum 10 days. If a patient is treated on a modified schedule, the GM-CSF dosage will be the same as the standard on days -4 to 0, but GM-CSF will be administered at 500 µg/m^2/day on the day of the first infusion of naxitamab, on the day before and on the day of the second infusion of naxitamab, and on the day before and on the day of the third infusion of naxitamab. The modified schedule ensures that the same total dosage of GM-CSF is administered in the standard and in the modified schedules.

Treatment cycles are repeated every 4 weeks (±1 week) until CR or PR followed by 5 additional cycles every 4 weeks (±1 week). Subsequent cycles are repeated every 8 weeks (±2 weeks) through 101 weeks from first infusion (see Figure 2) at the discretion of the investigator. This trial protocol is designed as a treatment trial securing that all patients receive the optimal number of treatment cycles for their disease. However, only few patients need treatment cycles repeated for two years. The average number of cycles given in the 12-230 was for responders (patients with CR/PR) 7.2 cycles and for non-responders 4.3 cycles. For the number of cycles to response the median was 2, minimum number of cycles was 1 and maximum number of cycles were 9. The 12-230 investigators had the following considerations when deciding the total number of treatment cycles. In case of:

- CR or PR: 5 additional cycles after CR/PR meaning in most cases 7 cycles in total
- SD: 7 cycles with 4 weeks interval followed by cycles with 8 weeks interval at the discretion of the investigator
- PD: Withdrawal of the patient at the discretion of the investigator

Figure 2 Treatment and response assessment schedule

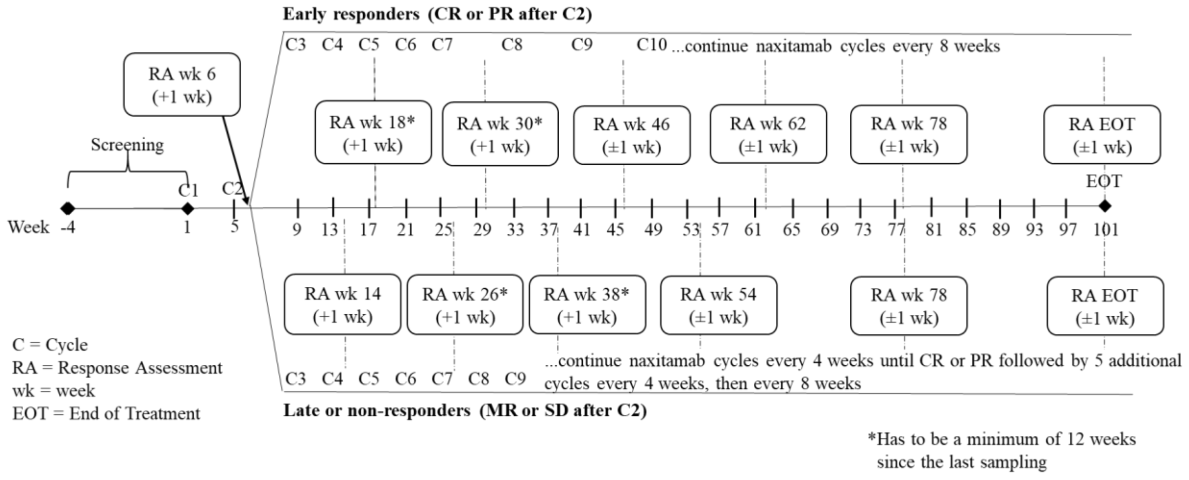

To modulate pain from naxitamab, patients should be pre-medicated, see sections 6.7.4, 6.7.5 and 7.2.2.

6.1.1 Screening/pre-treatment evaluation

The patient/patient's legal guardian must have received the patient information and signed the informed consent form prior to any trial-related procedures being performed. The outcome of the screening period (up to 25 days prior to first naxitamab infusion) will be evaluated before a patient will receive IMP in the trial. To be eligible for inclusion in the trial, the patient must meet all inclusion criteria and must not violate any of the exclusion criteria. Patient eligibility must furthermore be approved by the sponsor before the patient receive IMP in the trial. This is done by completing the patient eligibility form.

6.2 Data monitoring committee

An external Data Monitoring Committee (DMC) is established to review and evaluate accumulated data (e.g. severe and serious AEs) from the trial approximately every 3 months as well as ad-hoc. The purpose is to protect the safety of the patients and to evaluate the benefit-risk balance. The DMC will work according to a charter and provide recommendations on trial continuation, modification or termination. The outcome of the DMC meeting will be discussed and confirmed by the sponsor safety committee and communicated to the investigators if there are significant changes to trial conduct.

6.3 Follow-up

EOT measurements/evaluations should take place minimum 6 weeks (acceptable range 6-10 weeks) after the last infusion. However, there will be clinical situations where the EOT must be performed earlier than 6 weeks after last dose e.g. if the patient has PD or withdraws consent, see section 6.7.3. The EOT visit is of major importance for the study and all EOT measurements (see Table 1) must be performed before any new anti-NB treatments are started.

After EOT, a long-term FU period will start where patients should come for quarterly visits for 3 years. Standard long-term FU visit will include:

- Type of meeting (physical meeting/telephone call/skype meeting with either referral site doctor, nurse, patients relative or patient)
- Date and status for last confirmation of patients being alive

- Date and status of patients having progressive disease including information on the quality behind the assessment
- Information on all anti-cancer therapies
- Adverse events (section see section 10.4. For safety reporting in case of withdrawal, please see section 6.7.3)

In addition the investigator should in some situations ask the patient to come for response assessment at site according to Table 3.

Table 3 Expectations to response assessment after EOT

Long-term FU timepoint (months after EOT)	Patients with documented investigator assessed PD combined with initiation of new prohibited anti-cancer therapies*	All other patients
3, 6 and 9	Standard long-term FU visit	Standard long-term FU visit<br>+<br>Response assessment according to section 9.1.1 (local and independent review) at the investigator site.
All following visits	Standard long-term FU visit	Standard long-term FU visit

* Prohibited anti-cancer therapies as defined in section 7.2.3.

6.4 Recruitment period

It is estimated that ~60 months will be needed to recruit the planned number of patients.

6.5 End of trial

End of trial is defined as last patient's last visit in the long-term FU period. When patients have attended EOT visit they are defined as having ended treatment phase. When patients have attended the last visit in the long-term FU phase or die, whichever comes first, they are defined as having ended long-term FU.

6.6 Number of patients

Trial 201 plans to enroll 37 patients evaluable for objective response assessed by central review, screened negative for ADA and with no pre-planned radiotherapy for metastatic lesions (cohort 1). During discussion with regulatory authorities further data on PFS has been requested, which is why it has been decided to enroll additional patients, targeting a total of at least 85 patients in cohort 1. With an estimated screen failure rate of 25% it is likely that approximately 113 patients will be screened to reach 85 eligible cohort 1 patients. Additionally, eligible patients screened positive for ADA and/or pre-planned radiotherapy for metastatic lesions and/or by independent review assessed as non-evaluable for objective response at baseline will be included in a separate cohort 2. The enrollment will be stopped when the 85 eligible cohort 1 patients have been enrolled. The number of patients in cohort 2 is expected to consist of a maximum of 10 patients, therefore the maximum number of patients in the trial will be 85 + 10 patients. In summary the two cohorts are defined as follows:

Cohort 1 include patients:

- Screened negative for ADA *and*
- With no pre-planned radiotherapy for metastatic lesions *and*
- Fulfilling the protocol for baseline disease defined as:

- Evaluable disease at baseline in bone and/or bone marrow

Cohort 2 include patients:

- Screened positive for ADA *or*
- With pre-planned radiotherapy for metastatic lesions *or*
- Not fulfilling the protocol for baseline disease defined as:
  - No evaluable disease at baseline in bone and/or bone marrow

Cohort 1 is the cohort of primary interest. Cohort 2 serves subgroup analyses purposes. Disease status at enrollment (primary refractory disease or patients with incomplete response to salvage treatment) will be monitored to ensure there are at least 18 evaluable primary refractory patients in cohort 1. The patients will be recruited according to institutional guidelines.

6.7 Patient selection and withdrawal

The patient population for the trial comprises high-risk NB patients with primary refractory disease or incomplete response to salvage treatment in bone and/or BM. Patients with other prior anti-GD2 therapy are permitted to enroll in this trial. The patients will be enrolled in two separate cohorts according to section 6.6.

High-risk NB defined as (INRG (Cohn et al. 2009)):

- *MYCN*-amplified any INRG stage of any age and
- *MYCN*-nonamplified with INRG stage M patients, diagnosed at ≥18 months of age

Primary refractory disease
Defined as no relapse or PD but incomplete metastatic response (SD, MR, PR defined by INRC (Park et al. 2017) to treatment in bone and/or BM as documented either by histologic evidence of NB in bone or BM and/or abnormal ^{123}I-MIBG uptake in skeletal sites.
In addition, at trial enrolment, patient cannot be more than 18 months from initiation of chemotherapy and must have received at least 4 cycles of standard induction chemotherapy for high-risk NB comparable to the regimens applied in the following trials:

a) COG protocol 0532 or protocol A3973
b) MSKCC N5 or N7
   or
c) Rapid COJEC

Incomplete response to salvage treatment (secondary refractory disease)
Defined as relapse or PD and incomplete response (SD, MR, PR, but not PD, defined by INRC (Park et al. 2017)) to salvage chemotherapy or MIBG therapy immediately before enrollment.
Incomplete response to salvage treatment must be restricted to lesions in bone and/or BM as documented either by histologic evidence of NB in bone or BM and/or abnormal ^{123}I-MIBG uptake in skeletal sites. In addition, a minimum of two months from last MIBG/PET-CT documented relapse or PD prior to enrolment is required. The salvage therapies could include but are not restricted to:

a) Irinotecan, temozolomide or a combination of the two

b) MIBG therapy
c) Cyclophosphamide and topotecan
d) Topotecan and temozolomide
e) Topotecan, doxorubicin and vincristine
f) Ifosfamide, carboplatin and etoposide

To be eligible, the patient must meet all inclusion criteria and must not violate any of the exclusion criteria.

6.7.1 Inclusion criteria

1. Documented diagnosis of NB as defined per INRC as
   a. histopathology of tumor biopsy, or
   b. BM aspirate or biopsy indicative of NB by histology plus high blood or urine catecholamine metabolite levels or *MYCN* amplification, or
   c. MIBG-avid lesion(s)
2. High-risk NB patients with either primary refractory disease or incomplete response to salvage treatment (in both cases including SD, MR and PR) evaluable in bone and/or BM as defined in section 6.7. If disease is only present in bone the patient must have evaluable disease outside the radiation areas for being eligible in the trial, please see section 7.2.1. If disease is only present in the BM the involvement must be >5%.
3. Life expectancy ≥6 months
4. Age ≥12 months
5. Acceptable hematological status at screening, (hematological support is allowed if administered ≥1 week before screening procedure), defined as:
   a. Hemoglobin ≥8 g/dL (5.0 mmol/L)
   b. White blood cell count ≥1000/µL (1.0 x10^9/L)
   c. ANC ≥500/µL (0.5 x10^9/L)
   d. Platelet count ≥25,000/µL (25 x10^9/L)
6. Acceptable liver function defined as:
   a. ALT and AST ≤5 times ULN
   b. Bilirubin ≤1.5 x ULN
7. Acceptable kidney function defined as:
   a. eGFR >60 mL/min/1.73 m^2 calculated by the 2009 revised Bedside Schwartz Equation (Appendix 2)
8. Written informed consent from legal guardian(s) and/or patient in accordance with local regulations. Children must provide assent as required by local regulations.

6.7.2 Exclusion criteria

1. Any systemic anti-cancer therapy, including chemotherapy or immunotherapy, within 3 weeks of 1st dose of GM-CSF
2. Evaluable NB outside bone and BM defined as follows:

- MIBG-avid tumor: Definite MIBG uptake in tumor tissues outside bone and BM
- MIBG nonavid tumor: Definite uptake in tumor tissues outside bone and BM on FDG-PET

3. Actively progressing disease at trial entry according to Park criteria (Park et al. 2017) (see section 6.7)
4. Existing major organ dysfunction CTCAE >Grade 2, with the exception of hearing loss, hematological status, kidney and liver function
5. Active life-threatening infection
6. Prior treatment with naxitamab
7. Karnofsky/Lansky score < 50%
8. Pregnancy or a woman who is breast-feeding (women of child-bearing potential must have a negative pregnancy test at screening). A woman of child-bearing potential is excluded if she does not agree to use highly effective contraception for a period of 40 days after the last naxitamab infusion according to section 9.2.5. A sterilized or infertile woman is exempt from the requirement to use contraception after naxitamab treatment: she must have undergone surgical sterilization (hysterectomy, or bilateral ovariectomy).
9. Inability to comply with protocol requirements, including PK studies, as determined by the investigator
10. History of allergy or known hypersensitivity to GM-CSF, yeast-derived products, or any component of GM-CSF or naxitamab
11. History of anaphylactic reactions CTCAE grade 4 related to prior GD2 antibody therapy
12. NB in CNS within 6 months of 1st dose of GM-CSF
13. Prior treatment with omburtamab (mu8H9) within 6 months of 1st dose of GM-CSF
14. Patients who have had allogeneic hematopoietic stem cell transplantation (allo-SCT) or donor-lymphocyte-infusion (DLI). DLI or buffy coat infusion is defined as any kind of active allogenic lymphocyte suspension
    a. within 6 months of 1st dose of GM-CSF or
    b. with a lymphocyte count < 0.2 x10^9/L
15. Patients who received Hematopoietic Progenitor Cell (HPC) boost or "top-up" of allogenic stem cells (lymphocyte-depleted) within 2 months of 1st dose GM-CSF.
16. Any clinically meaningful abnormal finding in physical examination, vital signs, ECG, hematology, clinical chemistry, or urinalysis prior to inclusion into the trial, which in the opinion of the investigator, may put the subject at risk because of his/her participation in the study.

6.7.3 Withdrawal criteria

A patient should be withdrawn from treatment and enter long-term follow-up, if any of the following situations occur:

1. CTCAE Grade 4 anaphylaxis
2. CTCAE Grade 3 anaphylaxis not responding to medical intervention
3. CTCAE Grade 3 pain unresponsive to maximum supportive measures

4. CTCAE Grade 4 sensory neuropathy or Grade 3 sensory neuropathy that interferes with daily activities for more than 2 weeks
5. CTCAE Grade 4 bronchospasm
6. CTCAE Grade 3 bronchospasm not responding to medical intervention
7. Posterior reversible encephalopathy syndrome (PRES)
8. Atypical hemolytic uremic syndrome (HUS) in the absence of documented infection and resulting in renal insufficiency, electrolyte abnormalities, anemia, and hypertension
9. The investigator judges it necessary for medical reasons
10. The patient receives prohibited therapy and/or procedures during the trial, see section 7.2.3.
11. Pregnancy

A patient should be withdrawn from the trial at any time if:

- It is the wish of the patient/legal guardian for any reason

If the patient is withdrawn from the trial, the investigator should ensure that:

- The patient, whenever possible, irrespective of the reason for the withdrawal, will be examined as soon as possible according to the procedures for an EOT visit (see section 6.3)
- The investigator must follow the patients for AEs/SAEs according to section 10.4.
- Any SAE still ongoing after the end of the trial will be followed until the event has been resolved or the investigator assesses the SAE as being chronic or stable
- The date of the premature termination and the main reason is documented in the eCRF
- The investigator, in consultation with the patient/legal guardian, will decide upon future treatment based on clinical practice at the investigational site

6.7.4 Management of selected naxitamab Adverse Reactions

Pre-medication must be administered to prevent infusion-related reactions/allergic reactions and is specified in section 7.2.2. Patients should be monitored closely during the infusion and investigator should always use best clinical judgement in order to ensure the safety of the patient. Supportive therapies that may be needed during the infusion is presented in section 7.2.2.

In the following sections management of the selected adverse drug reactions is described separately for hypoxia, bronchospasm, hypotension, hypertension and “other infusion related reactions”. Finally, management of GM-CSF (sargramostim) related adverse reactions is described.

6.7.4.1 Management of Bronchospasm and Hypoxia

Hypoxia CTCAE Grade ≥3

Is the patient:

- Holding their breath?
- Having bronchospasm?

Act accordingly.

Bronchospasm with hypoxia CTCAE ≥3

- Pause naxitamab infusion

- Give nebulizer with beta2-adrenegic receptor agonist and inform the treating physician
- If the response is not satisfactory based on investigator's clinical judgement
    - Consider S2 inhalation (epinephrine racemic)
    - Consider additional IV antihistamine
    - Consider IV corticosteroids
- On the investigator's discretion, if the patient stabilizes and recovers from hypoxia (meaning bronchospasm CTCAE Grade ≤ 2), resume infusion at ~50% of previous infusion rate. If the patient continues to be stabilized, infusion speed can be resumed based on clinical judgement.
- Please refer to section 6.7.3 for withdrawal criteria

Bronchospasm CTCAE Grade 2

- Reduce infusion speed to ~50% of previous infusion rate
- Give nebulizer with beta2-adrenegic receptor agonist
- If the patient stabilizes and recovers (meaning bronchospasm CTCAE Grade ≤ 1), the infusion speed can be resumed based on clinical judgement.

6.7.4.2 Management of Hypotension

Hypotension Grade 2

- Initiate normal saline bolus 10 – 20 mL/kg IV over 5 – 15 min

Hypotension Grade 3 without other symptoms

- Decrease infusion rate to ~50%
- Initiate normal saline bolus 10 – 20 mL/kg IV over 5 – 15 min and inform the treating physician. Repeat vital signs after completion of the bolus and again 15 min later.
    - If the patient remains hypotensive, consult the treating physician, repeat bolus as described above (maximum a total of 60 mL/kg) and based on clinical judgement consider additional treatments as described below.
    - If the patient stabilizes, consider resuming to normal infusion speed based on clinical judgment

Hypotension Grade 3 with other symptoms such as bradycardia, hypoxia, not adequately responding OR Grade 4 hypotension

- Pause naxitamab infusion
- Initiate aggressive fluid resuscitation according to local standard of care (e.g. American College of Critical Care Medicine (ACCC) (Brierley et al. 2009).
- Consult the treating physician Repeat vital signs after completion of the bolus and again 15 min later.
    - If the patient remains hypotensive, repeat bolus as described above.
        - If resolution to CTCAE ≤ Grade 2: resume naxitamab infusion at ~50% of the previous infusion rate. On the investigator's discretion consider increasing infusion speed if the patient has stabilized and the infusion is well tolerated.
        - If the patient remains hypotensive consult the treating physician and based on clinical judgement, consider additional treatments as described below.

- If the patient is NOT warm and well-perfused **OR** do not respond satisfactorily to IV saline bolus treatments **OR do** respond to IV saline bolus but only temporarily, the treating physician should based on clinical judgement:
    - If the patient is difficult to arouse: give naloxone
    - If naloxone has no effect or based on clinical judgement: Consider inotropes according local standard of care (e.g. adrenalin IV/IM)
        - If desired effect is achieved and the hypotension is CTCAE ≤ Grade 2: On the investigator's discretion, resume naxitamab infusion at 50% of the previous infusion rate. If hypotension CTCAE Grade 3 occurs again consider halting naxitamab infusion based on the investigator's judgement.
        - If the patient continues to be hypotensive and difficult to arouse:
            - Permanently stop the current naxitamab infusion
            - Initiate appropriate treatment and observation on the investigator's judgement e.g. hospitalization in the intensive care unit.
- Next infusions to be initiated at the discretion of the investigator, on ~50% of the initial infusion rate prior to the event and the investigator should consider adjustments of the premedication.

6.7.4.3 Management of Hypertension outside of hypertension associated with acute pain

- Treat all possible causes first including pain, urinary retention, allergic reaction, anxiety, and agitation.
- Treat hypertension according to local standard of care (SoC)

If hypertension persists at the 90th percentile or higher of BP for age, height and sex according to NIH[1] guideline

- Before naxitamab infusion:
    - Consult the treating physician
- During naxitamab infusion:
    - Follow the patient closely with additional BP measurements
- After naxitamab infusion:
    - The patient should be followed closely with additional BP measurements
        - During the naxitamab treatment cycle: additional BP measurements between treatment days (on Day 2 and 4)
        - After naxitamab last dose in a cycle (Day 5): additional BP measurements the following 3 days (on Day 6, 7 and 8)
    - Physical examination Day 8

If hypertension persists at the 99th percentile or higher of BP for age, height and sex according to NIH[1] guideline

- Before naxitamab infusion:

[1] National Heart, Lung and Blood Institute. Blood Pressure Levels for Boys by Age and Height Percentile. Link: https://www.nhlbi.nih.gov/files/docs/guidelines/child_tbl.pdf

  - Treating physician must be consulted and the BP should be treated before infusion can be initiated (see below for details hypertension algorithm)
- During naxitamab infusion:
  - Hypertension seen during the infusion is often related to e.g. pain, anxiety, fluid retention. Notify the physician and treat the underlying cause.
  - Follow the patient closely with additional BP measurements
  - If the patient is not responding, consider other interventions on the discretion of the investigator
- After naxitamab infusion:
  - Start patient on isradipine 0.05 mg/kg/dose every 6 hr as needed or comparable therapy for acute hypertension according to local SoC (see naxitamab hypertension algorithm, Figure 3).
  - Admit to inpatient service for monitoring
  - If the patient does not respond to isradipine, consider permanent withdrawal from treatment
  - Enalapril (starting dose 0.08 mg/kg/dose once daily; max dose 5 mg; or equivalent angiotensin-converting enzyme (ACE) inhibitor) or amlodipine according to local SoC may be prescribed for the patient prior to future cycles of naxitamab as per the discretion of the investigator.
  - If the patient has a history of hypertension with naxitamab requiring enalapril/amlodipine on treatment days, consider holding the enalapril/amlodipine on Day 1 to avoid possible hypotension.

Figure 3 Hypertension algorithm for BP parameter >99th percentile by age

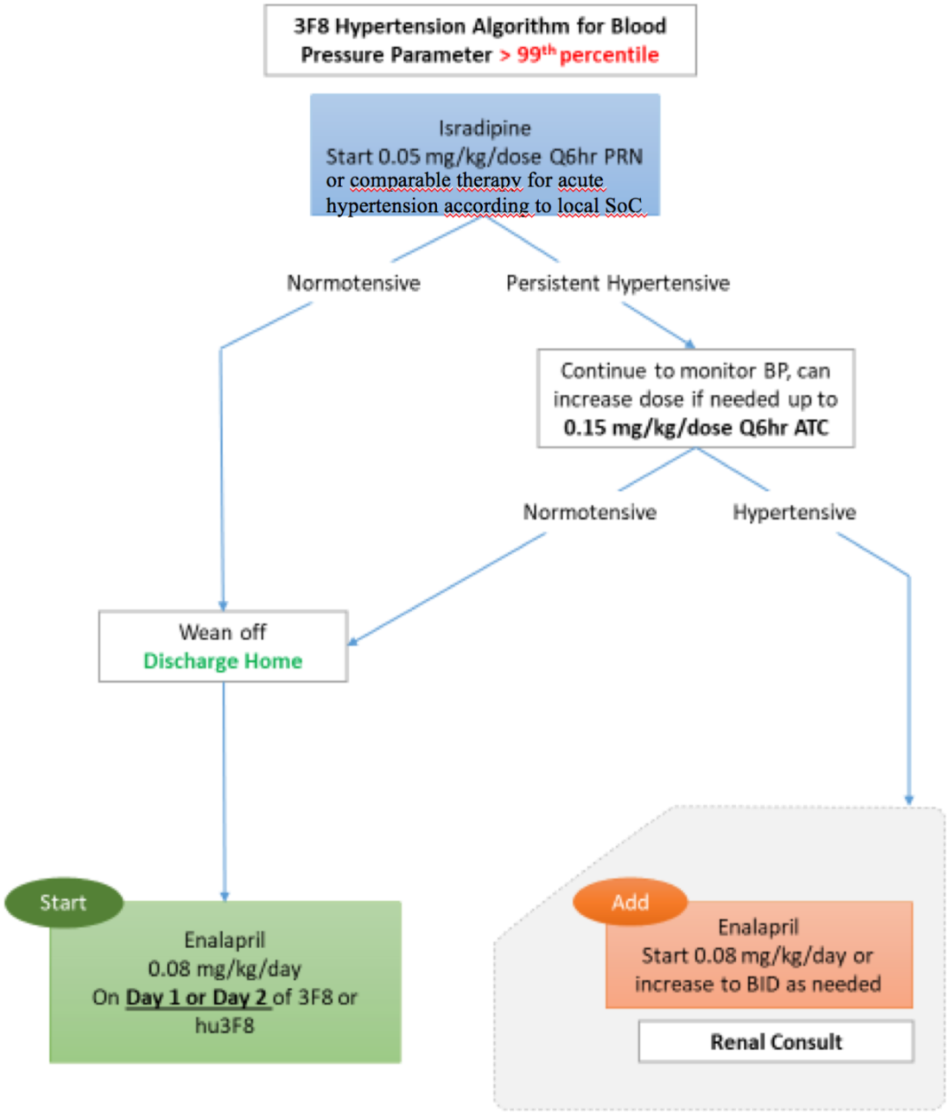

PRES

In case of increased risk of PRES in patients receiving naxitamab, (including high BP, headache, seizures, altered consciousness, and visual disturbance):

- Admit patient for observation and possible anti-hypertension therapy
- When BPs are stable <99th percentile for height according to NIH[1] patients can be discharged, but return the next day for check-up and follow the further instruction for hypertension above
- Please also refer to section 6.7.3 for withdrawal criteria

6.7.4.4 Management of Infusion-related Reactions CTCAE Grade ≥ 2, other than hypoxia, bronchospasm, hypotension, hypertension

- Pause naxitamab infusion
    - If resolution CTCAE ≤ Grade 1:

        - Resume naxitamab infusion at reduced rate of ~50% of the previous infusion rate and monitor closely.
        - On the investigator's judgement consider to gradually increase infusion rate up to the rate before the event.
        - If reaction occurs again, reduce naxitamab to the tolerated infusion rate throughout the remaining time of the infusion.
        - At the next naxitamab infusion, the infusion rate should be initiated at this reduced infusion speed. If well tolerated the infusion rate can be gradually increased based on the investigator's clinical judgement.
    - If no resolution:
        - On the investigator's judgement consider appropriate PRN medicine (see section 7.2.2)
            - If resolution CTCAE ≤ Grade 1:
                - Consider resuming naxitamab infusion at reduced rate of ~50% of the previous infusion rate as described above
            - If no resolution:
                - Depending on the treated symptoms and severity and based on the investigator's clinical judgement, consider further appropriate treatments, additional observation and whether to stop current infusion.

- Please also refer to section 6.7.3 for withdrawal criteria

6.7.5 Management of GM-CSF (sargramostim) Adverse Reactions

Stimulation of the BM can cause excessive production of both neutrophils (resulting in elevated ANC) and leucocytes. CBC must be checked before the first dose of GM-CSF and before administration of GM-CSF on days 1, 3, and 5. If ANC > 20 $x10^9$/L or WBC > 50 $x10^9$/L, GM-CSF should not be administered. GM-CSF can subsequently be resumed if values fall below the above-mentioned thresholds. Naxitamab should still be administered according to sections 6.1 and 7.1.1 even if ANC and/or WBC are above the defined thresholds.

7 TREATMENTS

7.1 Investigational medicinal products (IMPs)

Both GM-CSF and naxitamab are regarded as IMPs. The IMP must only be dispensed to patients who meet the eligibility criteria, see sections 6.7.1 and 6.7.2.

Table 4 IMP characteristics

IMP	Sargramostim (GM-CSF)	Naxitamab - humanized IgG1 mAb against GD2
Route:	Subcutaneous injection, per cycle	Intravenous infusion, 9 mg/kg per cycle
Treatment:	Days –4 to 0: 250 µg/m^2 per day<br>Days 1 to 5: 500 µg/m^2 per day	Day 1: 3 mg/kg per day<br>Day 3: 3 mg/kg per day<br>Day 5: 3 mg/kg per day
Pharmaceutical form:	Lyophilized 250 µg single use vial	Solution for infusion
Unit strength:	250 µg/vial	4 mg/mL

7.1.1 Administration of IMP for each patient

7.1.1.1 GM-CSF

GM-CSF should be administered at 250 µg/m^2 per day from Day -4 to Day 0 and 500 µg/m^2 per day at Day 1 to Day 5. GM-CSF and auxiliary supplies can be provided to the patient/parents to be administered at home after the patient/parents has received appropriate training on how to administer the drug. Thorough instructions will be given orally and in writing including instructions on how to store and administer the reconstituted GM-CSF at home. The patient/parents will be asked to fill in records of administration (patient diary). Auxiliary supplies include e.g. syringe, needles, sharps disposal container, gloves, alcohol wipes.

7.1.1.2 Naxitamab

Before any naxitamab infusions the patient should have a well-functioning intravenous catheter. The personal should be trained in resuscitation and treatment of severe hypotension according to local standard (Brierley et al. 2009).

Naxitamab should be infused at 3 mg/kg/day on Day 1, 3, and 5 for a total dose of 9 mg/kg per cycle. Administration of naxitamab should be adjusted according to section 6.7.4 if selected naxitamab adverse reactions are observed. If any events occur fulfilling a withdrawal criterion the patient must be withdrawn from treatment immediately and enter long-term follow-up, see section 6.7.3.

<u>At cycle 1 Day 1 (C1D1):</u>
Naxitamab should be infused over approx. 60 min.

<u>All subsequent naxitamab infusions:</u>
Naxitamab should be infused over approx. 30 min.

<u>Special circumstances:</u>
At the investigator’s discretion (approx.) 60 min infusion can be chosen at Day 1 for the following cycles if:

- The patients during C1D1 experienced related hypotension:

    - Grade 4 OR
    - Grade 3 not adequately responding
- At the investigators discretion due to severe infusion related adverse drug reactions not adequately responding to interventions

At infusion start the infusion rate is set to reach the 30/60 min goal. In case of interventions due to mitigations required according to section 6.7.4 the infusion time will become longer meaning that the 30/60 min infusion time is a minimum infusion time, when no pauses or infusion rate reductions are required.

Please refer to sections 6.7.4, 6.7.5 and 7.2.2 and the IMP manual for more details of storage conditions, preparation and administration of the IMPs.

7.1.2 Drug accountability and compliance check

The investigator or designee (e.g. pharmacist) is responsible for ensuring adequate accountability of all used, partially used and unused IMP. This includes acknowledgment of receipt of each shipment of trial product (quantity and condition), patient dispensing records, and returned or destroyed trial product. Dispensing records will document quantities received from sponsor and quantities dispensed to patients, including lot and vial number, date dispensed, patient identifier number, patient initials, and the initials of the person dispensing the medication. The patient/parents are provided with forms to record the administration of GM-CSF at home and bring these records to all hospital visits.

If applicable, the clinical research associate (CRA) will evaluate the site's standard operating procedure (SOP) for IMP disposal/destruction in order to ensure that it complies with sponsor's requirements. If applicable, drug may be returned or destroyed on an ongoing basis as agreed during the trial after drug accountability has been performed. At the end of the trial, following final drug inventory reconciliation by the CRA, the trial site will dispose and/or destroy all unused IMP supplies, including empty containers, according to their procedures after obtaining written approval from sponsor. If the site cannot meet sponsor requirements for disposal, arrangements will be made between the site and sponsor or its representative for destruction or return of unused IMP supplies. All drug supplies and associated documentation will be periodically reviewed and verified by the CRA over the course of the trial. Please refer to the IMP manual for more information.

7.1.3 Packaging and labelling

Packaging, labelling and release of naxitamab and GM-CSF will be performed under the responsibility of the IMP supplier in accordance with good manufacturing practice (GMP) and national regulatory requirements. The labels will be in accordance with Annex 13, EudraLex, volume 4, and national requirements. Each dispensing unit is uniquely numbered with a vial number (Vial No.)

7.1.4 Storage and handling

The Pharmacy will ensure that the IMPs will be stored at appropriate conditions in a secure location with controlled access. In order to secure proper IMP handling, the storage temperature must be recorded and evaluated. Any deviations in storage temperature must be reported immediately to sponsor and the IMP must not be used until acceptance from the

sponsor is received. The IMP should be stored according to the label until it is dispensed to the patient. Extended information about IMP handling can be found in the IMP manual.

7.1.5 Patient compliance

Clinical personnel at the site will record the start and end time of infusions and injection/infusion site location of all administrations (GM-CSF and naxitamab) in the source notes, which will be captured in the electronic case report form (eCRF). Any reasons for non-compliance should be documented.

7.1.6 Overdose, medication errors, misuse and abuse

An overdose is defined as a patient receiving a dose of the IMP in excess of that specified in this protocol.

Medication errors and uses outside what is foreseen in the protocol, including misuse and abuse of the product may include:

- Administration of wrong drug
- Wrong route of administration, such as intramuscular instead of IV
- Accidental administration of a lower or higher dose than intended. The administered dose must deviate from the intended dose to an extent where clinical consequences for the trial patient were likely to happen as judged by the investigator, although they did not necessarily occur

Overdose and medication errors (exceeding ± 10% as compared to protocol-specified dose) should be reported as protocol deviations to the CRA or in the eCRF. If an overdose, medication error, misuse or abuse results in an AE, the AE must also be reported in the eCRF. If the event qualifies as serious it must be reported using a paper Safety Form in addition to the AE form in the eCRF.

7.2 Concomitant therapy

7.2.1 Therapy allowed during trial

Any treatment, which is considered necessary for the patient's wellbeing (including supportive care) and which is not defined as prohibited therapies in section 7.2.3 and is assessed not to interfere with the assessment of safety and efficacy of the IMP under evaluation, may be given at the discretion of the investigator.

Radiotherapy to primary site
For patients who are enrolled without having previously received external-beam radiotherapy to the primary site, this standard consolidative therapy for high-risk NB should be performed prior to the screening MIBG/PET scans. In circumstances where the patient is in a critical state with an urgent need of systemic treatment from this trial protocol, the patient may receive the standard consolidation radiotherapy to primary site, after completion of at least one treatment cycle, preferably between cycle 1 and cycle 2. The site for external beam radiotherapy to the primary tumor site must be predefined and recorded in the CRF at baseline and has to be accepted by medical expert during the approval of the patient's eligibility form.

Radiotherapy to metastatic sites

For patients who are enrolled where it has been judged clinically necessary to treat with external-beam radiotherapy to the metastatic site(s), this treatment should be performed prior to the screening MIBG/PET scans. In circumstances where the patient is in a critical state with an urgent need of systemic treatment from this trial protocol, the patient may receive external-beam radiotherapy for sites with bulky/measurable bony metastasis during trial. The radiation should be given after completion of at least one treatment cycle, preferably between cycle 1 and cycle 2. The metastatic lesions for external beam radiotherapy must be predefined and recorded in the CRF at baseline and lesions must be accepted by medical expert during the approval of the patient's eligibility form. For both scenarios the patient must have evaluable disease outside the radiation areas for being eligible in the trial.

7.2.2 Pre-medications prior to naxitamab infusions

Infusion with naxitamab may be associated with pain. The pain pattern generally involves the abdomen, lower back, and sometimes the chest and tends to spread peripherally to the ankles and the feet. It usually starts during the first few min of the infusion and can last for up to one hour after the infusion is completed. Due to the pain, many of the patients will "hold their breath" and it is highly recommended that all sites are equipped with humidified oxygen inhalation equipment.

A pre-medication scheme is needed to mitigate expected AEs, including pain associated after infusion of naxitamab. As a marked intra-patient variation in the naxitamab-associated pain is observed, the recommendation relates to the first infusion. Pre-medications related to mitigation of pain associated with subsequent infusions of naxitamab will be adjusted according to the treating physician and all medications including opioid usage should be captured under concomitant medication.

Administer the following pre-infusion medications to reduce the risk of infusion-related reactions, allergic reactions, nausea/vomiting and if applicable anxiety to all patients prior to every infusion of naxitamab:

At home

- Gabapentin given as a dose of 5 to 10 mg/kg with a max dose of 600 mg, and titrated during the first 3 days as follows:
  - Day -4: once daily
  - Day -3: twice daily
  - Day -2 and onwards until Day 7: 3 times daily

  According to product resume, the total daily dose of Gabapentin in patients 6 years of age and older is 25–35 mg/kg/day.

Premedication

- Saline solution
  - Patients should be preloaded with 1 hour saline bolus 10 mL/kg just prior to the start of all naxitamab infusions.
- Opioids

  PO opioids are preferred over IV in order to diminish IV related side effect such as hypotension, respiratory depression and decreased responsiveness

  - Oxycodone 0.1 – 0.2 mg/kg with a max dose of 5 mg PO (45 – 60 min before naxitamab infusion) OR equivalent dose of PO opioids OR
  - In cases where PO administration is not feasible (15 min before naxitamab infusion)
    - Hydromorphone 0.00375 – 0.015 mg/kg IV administration over 2 - 10 min **OR**
    - Morphine Sulfate/ Morphine Sodium Chloride 0.025-0.1 mg/kg given IV over 2-10 min

- Corticosteroids (30 min – 2 h prior to naxitamab infusion)
  - All patients at the first infusion in the first cycle must receive:
    - IV methylprednisolone 2 mg/kg with a max dose of 80 mg OR equivalent corticosteroid dose
  - The same corticosteroid premedication should be given in the following circumstances:
    - At the first infusion in the following cycle if the patients had a Grade 3 bronchospasm/anaphylaxis at the last cycle's first infusion
    - At the following naxitamab infusion if the patients had a Grade 3 bronchospasm/anaphylaxis at the last infusion
    - At the investigator's discretion when infusion related reactions are not satisfiable controlled by the other premedications.

- Antipyretics (30 min before naxitamab infusion)
  - Paracetamol (acetaminophen) 10 mg/kg to maximum 15 mg/kg (max 750 mg total dose) PO or IV

- Antihistamine (30 min before naxitamab infusion)
  - Hydroxyzine 0.5 – 1 mg/kg (max 50 mg) PO/IV, **OR**
  - Diphenhydramine 0.5 – 1 mg/kg (max 50 mg) IV **OR**
  - Cetirizine <20 kg: 2.5 mg; >20 kg: 5 mg; If > 12y AND > 30 kg: 10 mg PO **OR**
  - Loratadine 5 mg for patients 2 – 5 yr; 10 mg for patient > 5 yr PO **OR**
  - Equivalent according to local SoC

- Antiemetics (30 min before naxitamab infusion)
  - Ondansetron 5 mg/m^2 **OR**
  - Equivalent according to local SoC

- Anxiolytics, if applicable
  - Lorazepam 0.01 – 0.02 mg/kg (max 1mg) IV PRN **OR**
  - Equivalent according to local SoC

- Proton-pump inhibitors (PPI) or H_2 antagonist PRN according to local SoC.

In case of inadequate pain control or hypersensitivity to the above-mentioned medications, alternative strategy to mitigate treatment-induced pain can be introduced at the discretion of the treating physician and after approval of sponsor Medical Expert. It is well known from treatment with anti-GD2 monoclonal antibodies, that ketamine can be a useful alternative to mitigate treatment-induced pain (Tong et al. 2015). In case of inadequate pain control with opioids, ketamine can be used according to local SOC after approval of sponsor Medical Expert.

Supportive Therapy

- At the onset of pain, ready as pre-charged syringes:
    - Hydromorphone 0.00375 – 0.015 mg/kg given IV over 1 – 5 min, **OR**
    - Morphine sulfate/ Morphine Sodium Cloride 0.025 – 0.1 mg/kg given IV over 1 – 5 min
    - Opiate doses may be repeated every 5 min for a total of 4 doses
    - Additional doses may be ordered and given PRN at the discretion of the treating physician

- PRN (ready at bedside)
    - Lorazepam 0.01-0.02 mg/kg PO or IV (max 1 mg) or equivalent according to local SoC
    - Dexclorfeniramine 0.15 mg/kg IV (max 5 mg) **OR** equivalent H1 receptor blocker according to local SoC
    - NSAID according to local SoC
    - Methylprednisolone 2 mg/kg IV with a max dose of 80 mg (In case glucocorticosteroid was not given as pre-medication) OR equivalent corticosteroid dose. If pre-medicated with glucocorticosteroid dose reduction should be considered on the investigator's discretion.
    - Adrenalin (epinephrine) 0.01 mg/kg IV or IM (max 0.5 mg)
    - Salbutamol nebulized < 20 kg: 2.5 mg; >20 kg: 5 mg, Levalbuterol **OR** comparable selective beta2-adrenergic receptor agonist (bronchodilator), according to local SoC. PRN bronchospasm
    - Racepinephrine OR equivalent S2-inhalant: PRN stridor
    - Naloxone should be readily available to revert potential acute cases of respiratory depression: Naloxone IV 1 mcg/kg/doses (to be repeated every 2-3 min until response)
    - Saline bolus: 20 mL/kg IV according to section 6.7.4
    - Cold and Hot Pads to be applied locally in any body area ad libitum
    - Oxygen mask

Discharge criteria

- Vital signs are stable and satisfactory
    - Minimum of 2 hours after completion of treatment, OR 2 hours after the last dose of any IV opioid.

7.2.3 Prohibited therapy and procedures during the trial

At baseline of the trial the therapies below are not allowed:

- Immunosuppressive (not including local steroid) within a month before the first dose of GM-CSF

During the treatment period of the trial the therapies below are not allowed:

- Any additional systemic anti-cancer therapy (including stem cell infusions, HPC boosts (top-ups) and DLIs)
- Any local radiotherapy not pre-defined at baseline
- Any live vaccines
- IV immunoglobulin
- Any IMP not defined in this protocol

7.3 Treatment after discontinuation of IMP

Any treatment which is deemed safe and justified can be administered according to clinical practice and at the discretion of the investigator.

8 ENDPOINTS

8.1 Primary endpoint

1. ORR during the naxitamab treatment period, centrally assessed according to the INRC

8.2 Secondary endpoints

1. Safety will be evaluated by the incidence of AEs and SAEs graded according to CTCAE, version 4.0
2. DoR, defined as the time from first objective response (CR or PR) to PD; data will be censored at the date of last disease evaluation before new anti-NB treatment
3. Complete response rate, during the naxitamab treatment period, centrally assessed according to the INRC
4. ORR, during the naxitamab treatment period, investigator assessed according to INRC
5. Assessment of the PK of naxitamab
6. Assessment of ADA formation
7. IV opioid use during cycle 1 defined as total dosage of IV morphine (or equivalent opioid) administered 2 hours before infusion until 4 hours after end of infusion of naxitamab
8. IV opioid use for each cycle during the trial defined as total dosage of IV morphine (or equivalent opioid) administered 2 hours before infusion until 4 hours after end of infusion of naxitamab
9. Number of hospitalization days related to naxitamab during cycle 1, defined as number of overnight stays. Hospitalizations required solely for protocol-specified assessments (e.g., PK sampling) or non-medical circumstances are excluded
10. Number and percentage of infusions done in an outpatient setting
11. In patients with positive ADA at trial inclusion, safety will be evaluated by the incidence of AEs and SAEs graded according to CTCAE, version 4.0
12. Happiness and activity levels measured over time assessed by caretaker

8.3 Secondary endpoints including long-term follow-up

1. PFS, defined as the time from the first 1st infusion of naxitamab until PD or death, whichever comes first; data will be censored at the date of last disease evaluation before new anti-NB treatment
2. OS, defined as the time from the first infusion of naxitamab until death; data will be censored at last date known to be alive

9 PROCEDURES AND ASSESSMENTS

9.1 Efficacy assessments

9.1.1 Primary endpoint assessments

The primary endpoint is ORR. ORR will be based on imaging (by CT/MRI and ^{123}I-MIBG scans/FDG-PET or MIBG-SPECT/CT) and BM histology (BM examination should comprise bilateral BM aspirates as well as BM biopsies, 2+2 samples, as per the INRC) (Park et al. 2017) (Table 5, Table 6, Table 7). The NB disease status at trial entry is confined to bone and/or BM sites, thus CT/MRI is used to detect PD in soft tissue, i.e., outside the bones and BM.

Table 5 Tumor response at metastatic bone sites

Response	Anatomic + MIBG (FDG-PET*) Imaging
CR	MIBG uptake or FDG-PET uptake (for MIBG-nonavid tumors) of nonprimary lesions resolves completely
PR	No new lesions AND<br>≥50% reduction in MIBG absolute bone score (relative MIBG bone score ≥0.1 to ≤0.5) or<br>≥50% reduction in number of FDG-PET–avid bone lesions§
PD	Any of the following:<br>-Any new soft tissue lesion detected by CT/MRI that is also MIBG avid or FDG-PET avid<br>-Any new soft tissue lesion seen on anatomic imaging that is biopsied and confirmed to be NB or ganglioneuroblastoma<br>-Any new bone site that is MIBG avid<br>-A new bone site that is FDG-PET avid (for MIBG-nonavid tumors) AND has CT/MRI findings consistent with tumor OR has been confirmed histologically to be NB or ganglioneuroblastoma<br>-Relative MIBG score ≥1.2§
SD	Neither sufficient shrinkage for PR nor sufficient increase for PD of nonprimary lesions
Abbreviations:	CR, complete response; CT, computed tomography; FDG, [18F] fluorodeoxyglucose; MIBG, metaiodobenzylguanidine; MRI, magnetic resonance imaging; PD, progressive disease; PET, positron emission tomography; PR, partial response; SD, stable disease

* Used for MIBG-nonavid tumors
§ Relative MIBG score (based on Curie) is the absolute score for bone lesions at time of response assessment divided by the absolute score for bone lesions at baseline before therapeutic interventions

Table 6 BM metastatic response

Response	Cytology/Histology
CR	BM with no tumor infiltration on reassessment, independent of baseline tumor involvement
PD	Any of the following:<br>-BM without tumor infiltration that becomes > 5% tumor infiltration on reassessment OR<br>-BM with tumor infiltration that increases by > twofold and has >20% tumor infiltration on reassessment
MD	Any of the following:<br>-BM with ≤5% tumor infiltration and remains >0 to ≤5% tumor infiltration on reassessment OR<br>-BM with no tumor infiltration that has ≤5% tumor infiltration on reassessment OR<br>-BM with >20% tumor infiltration that has >0 to ≤5% tumor infiltration on reassessment
SD	BM with tumor infiltration that remains positive with >5% tumor infiltration on reassessment but does not meet CR, MD, or PD criteria
NOTE. In the case of discrepant results between aspirations or core biopsies from two or more sites taken at the same time, the highest infiltration result should be reported using the criteria in this table<br>Abbreviations: BM, bone marrow; CR, complete response; MD, minimal disease; PD, progressive disease; SD, stable disease	

Objective response will be defined by combining response of the individual components (i.e., bone and BM disease).

Table 7 Determination of objective response

Response	Criterion
CR	All components meet criteria for CR
PR	PR in at least one component and all other components are either CR, MD* (BM), PR (bone), or NI†; no component with PD
MR	PR or CR in at least one component but at least one other component with SD; no component with PD
SD	SD in one component with no better than SD or NI† in any other component; no component with PD
PD	Any component with PD
Abbreviations: BM, bone marrow; CR, complete response; MD, minimal disease; MR, minor response; NI, not involved; PD, progressive disease; PR, partial response; SD, stable disease<br>* For BM assessment only.<br>† Site not involved at trial entry and remains uninvolved.	

Imaging with MIBG must utilize ^{123}-iodine. Imaging with ^{124}I-MIBG PET has not been incorporated in any treatment assessment criteria or compared head-to-head to ^{123}I-MIBG with documented improvement in patient-relevant outcome. For patients with MIBG non-avid lesions, ^{18}F-FDG PET (Park et al. 2017) should be used.

If a new soft lesion is identified on CT/MRI at FU timepoint, the lesion should be either ^{123}I-MIBG or FDG-PET positive to qualify PD.

BM analyses should comprise bilateral aspirates and bilateral biopsies (2+2) as per the INRC and will be reviewed locally and centrally. The investigator will collect the local response assessment in the CRF, and treatment decisions will be based upon the local response assessment.

Imaging and BM examination will be performed during screening and after cycle 2 (C2) in all patients. At screening, imaging and BM examinations must be obtained at least 10 calendar days after end of any prior anti-cancer treatment.
- For patients with CR or PR at week 6 (post C2): repeat sampling at weeks 18 (+1 week), 30 (+1 weeks), 46 (±1 weeks), 62 (±1 weeks) and 78 (±1 week). The repeat sampling planned at week 18 and 30 has to be scheduled with a minimum of 12 weeks since last sampling.
- For patients with MR or SD at week 6 (post C2): repeat sampling at weeks 14 (+1 week), 26 (+1 week), 38 (+1 week), 54 (±1 week), and 78 (±1 week). The repeat sampling planned at week 26 and 38 has to be scheduled with a minimum of 12 weeks since last sampling.

Additionally, for patients in long-term FU response assessment should be performed at 3, 6 and 9 months after EOT (see Table 3).

For imaging, a charter will be prepared prior to trial start including minimum technical criteria for patient preparation, image capture details, and quality controls of CT, MRI and gamma cameras, and control of the image quality. Per imaging charter, serum glucose determination must be performed prior to the administration of FDG (PET only). Please refer to the charter for more details. Reading of images for the primary efficacy analysis will be performed centrally by a panel of independent expert reviewers blinded to clinical information. A reading manual will be in place prior to any readings. The reading results of individual readers and a consensus will be captured. More detailed information will be provided in the imaging manual.

9.1.2 Secondary endpoint assessments

9.1.2.1 Duration of response (DoR)

DoR is defined as the time from first objective response to PD. Data will be censored at the date of last disease evaluation before new anti-NB treatment.

9.1.2.2 Complete Response Rate

The complete response rate is defined as the fraction of patients experiencing a CR according to INRC criteria (Table 5, Table 6, Table 7) during the treatment period.

9.1.2.3 Progression-Free Survival (PFS)

PFS is defined as the time from the 1st infusion of naxitamab until PD or death, whichever comes first. Data will be censored at the last disease evaluation before new anti-NB treatment.

9.1.2.4 Overall survival (OS)

OS is defined as the time from first infusion of naxitamab until death from any cause. Data will be censored at last date known to be alive.

9.1.2.5 PK of naxitamab and ADA formation

Serum concentrations of naxitamab will be determined before, during and after cycle 1 and 2 before every subsequent treatment cycle and at the EOT (Table 8). Blood samples must never be drawn from the naxitamab infusion line.

Table 8 Timing of serum collection for PK and ADA at cycle 1

Day	Treatment	Serum Collection Times
1	naxitamab	PK:<br>• Pre-naxitamab<br>• 15min (±2 min) after start of naxitamab infusion<br>• Right before the end of naxitamab infusion<br>• 10min (±2 min) post-naxitamab infusion<br>• 1h (±5 min)<br>• 3h (±10 min)<br>• 6h (±20 min)<br>ADA:<br>• Pre-naxitamab
2		PK:<br>• 24h (±1h)
3	naxitamab	PK:<br>• Pre-naxitamab<br>• 15min (±2 min) after start of naxitamab infusion<br>• Right before the end of naxitamab infusion<br>• 10min (±2 min) post-naxitamab infusion
5	naxitamab	PK:<br>• Pre-naxitamab<br>• Right before the end of naxitamab infusion<br>• 10min (±2 min) post-naxitamab infusion
8		PK:<br>• 168 h (±24h)
12		PK and ADA:<br>• 264h (±24h)
17		PK:<br>• 384h (+48h/-24h)
24		PK:<br>• 552h (-24h)

Table 9 Timing of serum collection for PK and ADA at cycle 2

Day	Treatment	Serum Collection Times
1	naxitamab	PK:<br>• Pre-naxitamab<br>• Right before the patient is discharged or minimum 2 hours after completion of infusion<br>ADA:<br>• Pre-naxitamab
3	naxitamab	PK:<br>• Right before the patient is discharged or minimum 2 hours after completion of infusion
5	naxitamab	PK:<br>• Right before the patient is discharged or minimum 2 hours after completion of infusion
Day of scanning / BM biopsy/aspirate		PK:<br>• Same day of scanning or BM biopsy/aspirate (post cycle 2)

For the analysis of ADA formation, blood samples will be collected:

- During cycle 1 as described in Table 8
- During cycle 2 as described in Table 9

- For all subsequent treatment cycles: pre-naxitamab infusion
- At EOT

There will be no dose modification if the investigator suspects that ADA has developed.

PK/ADA should be drawn at the EOT visit (acceptable range is 6-10 weeks after the last infusion) and before start of any new anti-cancer treatment.

9.2 Other

9.2.1 Demographics

Information on the patient's demographics will be collected during screening in the eCRF. The following demographic information will be recorded:

- Age or date of birth if allowed by local legislation
- Gender
- Ethnic origin
- Race
- Other baseline characteristics will be recorded if applicable:
    - Current average alcohol consumption per week
    - Smoking status

9.2.2 Physical examination

The physical examination should include an assessment of general appearance and a review of systems (dermatologic, head, eyes, ears, nose, mouth/throat/neck, thyroid, lymph nodes, and respiratory, cardiovascular, gastrointestinal, extremities, musculoskeletal, neurologic systems). A physical examination should be performed at screening, prior to first infusion of naxitamab in each cycle and otherwise as clinically indicated.

9.2.3 Height and body weight

Height (without shoes) must be measured at screening and recorded in the eCRF rounded to nearest centimeter. Body weight (without overcoat and shoes) will be measured at screening and before each administration with IMP (naxitamab) and recorded in the eCRF rounded to nearest kilogram. The weight and height will be measured and body surface area (BSA) will be calculated before each treatment cycle with naxitamab and GM-CSF for calculation of the IMP doses; i.e. GM-CSF doses according to the last measured weight/height and naxitamab doses according to the weight at first day of each cycle. Instructions regarding the related treatment scheme will be given to the patient/parents.

9.2.4 Performance test

Performance test will be performed prior to cycles 1, 4, 7, 10, 13 and at EOT. The scales to be used are: Lansky for children < 16 years of age and Karnofsky for adolescents or adults ≥ 16 years of age.

9.2.5 Non-child-bearing potential/serum pregnancy test

Non-child-bearing potential in women will be confirmed by one of the following:

- Women/girls have not reached menarche or
- Women/girls have not had menses within the past 12 months and have a follicle-stimulating hormone (FSH) ≥40 IU/L or
- Women/girls have not had menses within the past 24 consecutive months if an FSH measurement is not available or
- Women/girls have undergone surgical sterilization (e.g., hysterectomy, or bilateral oophorectomy, or bilateral salpingectomy).

Women of child-bearing potential must have a negative pregnancy test at screening and are excluded if they do not agree to use highly effective contraception for a period of 42 days after the last naxitamab infusion such as:

- Oral, intravaginal or transdermal combined (estrogen and progestogen containing) hormonal contraception associated with inhibition of ovulation.
- Oral, injectable or implantable progestogen-only hormonal contraception associated with inhibition of ovulation.
- Intrauterine device (IUD)
- Intrauterine hormone-releasing system (IUS)
- Bilateral tubal occlusion
- Vasectomised partner provided that partner is the sole sexual partner of the WOCBP trial participant and that the vasectomised partner has received medical assessment of the surgical success.
- Sexual abstinence

A sterilized or infertile woman is exempt from the requirement to use contraception after naxitamab treatment: she must have undergone surgical sterilization (hysterectomy, or bilateral ovariectomy). If a patient is assessed to be of child-bearing potential, either at screening or become so during the course of the trial, a serum pregnancy test should be performed prior to each treatment cycle.

Naxitamab, has not been tested in pregnant or breast-feeding women, but IgG1 antibodies, in general, are known to cross the placental barrier. As naxitamab mediates ADCC and CDC of GD2 expressing cells, there is a potential for fetal harm. It is theoretically possible that relevant systemic concentrations may be achieved in woman of childbearing potential (WOCBP) from exposure to seminal fluid.
Male patients must use contraception (condom) for a period of at least 42 days after last treatment with naxitamab.

9.2.6 Research blood

Effector functions of mAbs including naxitamab can depend on their Fc functions, i.e., binding to fragment crystallizable receptor (FcR) in ADCC or binding to C1q in CDC. This affinity is determined by polymorphic alleles which determine mAb effectiveness and patient outcome. Heparinized blood for genomic DNA for FcgRIIa (CD32a) and FcgRIIIa (CD16a) gene polymorphism will be drawn during screening and analyzed by a central laboratory (Cheung et al. 2006).

9.2.7 Medical and surgical history

Information on the patient’s medical history, past and all current diseases (including surgical procedures) and general health, will be collected if available during screening and includes, but is not limited to:

- General medical history
- Tumor characteristics that allow calculation of risk classification, e.g., *MYCN* status, DNA index, disease stage and LDH at the time of diagnosis, and pathology classifications.
- Any prior treatment for NB, e.g., frontline therapy and salvage regimens, responses to prior therapy, DoRs, sites of relapse, etc.

A concomitant illness is any illness, other than the disease being investigated, which is present at trial start or found as a result of the screening procedure. The information collected for medical history and concomitant illnesses includes:

- Diagnosis
- Date of onset
- Date of resolution
- Date of relapse

Any clinically significant worsening of a concomitant illness that occurs after a patient provided consent must be reported as an AE (section 10).

9.2.8 Vital signs & ECG

Vital sign measurements must include heart rate, respiratory rate, body temperature and blood pressure. Additionally, peripheral oxygen saturation (SpO_2) must be measured when clinically indicated. Temperature must be measured by using the same method (e.g., ear thermometer) each time. Vital signs must as a minimum be assessed at screening, in connection with each naxitamab infusion as outlined below and at EOT:

- Prior to pre-medication (e.g., hydromorphone or morphine)
- Prior to start of naxitamab infusion
- ~30 min after naxitamab infusion
- Prior to each hydromorphone or morphine IV therapy as possible/feasible (no temperature)
- At completion of naxitamab infusion (no temperature)
- At completion of the flush
- ~15 min after completion of the flush (no temperature)
- Every hour while patient is recovering in the outpatient clinic

Additional measurements must be done when clinically indicated and reported in the eCRF providing data for severity and onset/stop time for the event. Clinically indicated is defined as when a vital sign parameter is part of an AE on an infusion day. For example, if hypoxia is reported on an infusion day, abnormal SpO_2 measurements before, during and after the event should be reported.

ECG is a local assessment at screening, before cycle 2 and cycle 4 and at EOT. If the patient needs sedation before ECG, the ECG can be performed in connection with imaging, hence if no imaging is planned for cycle 4, the ECG can be postponed until next imaging.

9.2.9 Hematology and clinical chemistry

Hematology and clinical chemistry will be measured by a local laboratory during screening and with all treatment cycles and will assess liver and kidney function blood tests (ALT, AST, alkaline phosphatase, albumin, bilirubin, LDH, BUN and creatinine) and serum electrolytes (sodium, potassium, chloride and bicarbonate). All Grade 4 laboratory abnormalities must be re-tested within 24 hours after receiving results.

CBC and differential counts should be performed during screening and with all treatment cycles and should at a minimum include:

CBC	Differential counts
White blood cell count (WBC)<br>Red blood cell count (RBC)<br>Hemoglobin (HgB)<br>Hematocrit (Hct)<br>Platelet count	Neutrophils (% and absolute)<br>Lymphocytes (% and absolute)<br>Monocytes (% and absolute)<br>Eosinophils (% and absolute)<br>Basophils (% and absolute)

The CBC and differential counts should be repeated on sequential days during all treatment cycles if ANC was > 20 $x10^9$/L or WBC > 50 $x10^9$/L on the previous day.

9.2.10 Pain assessment

Pain scores will be assessed for all naxitamab infusions.

Pain scores will be assessed at the following timepoints: (a) prior to start of naxitamab infusion, (b) worst pain during infusion, to be assessed at end of infusion, (c) pain ~15 min after end of infusion, and (d) prior to discharge.

For each patient the same pain scale must be used during all naxitamab infusions. The FLACC scale for patients ≤5 years old at screening and Wong-Baker FACES® pain scale for patients 6 years or older at screening.

If it is deemed by investigator or other site staff that a patient of at least 6 years of age cannot comply with the Wong-Baker faces pain scale it is permitted to use the FLACC scale as long as it is used consistently throughout all naxitamab infusions.

9.2.11 Quality of Life

There is currently no validated, disease-specific QoL tool available in neuroblastoma. Furthermore, the patient group is highly heterogenous in age, culture and languages. Combined with the fact that the children are extremely ill and the number of clinical assessments therefore should be held at the lowest possible level, it was decided to use a simple, intuitive VAS scale focusing on activity and happiness as a proxy for assessing QoL.

Each evening from C1, day -4 to C2, day 7 (i.e. 2 days after last infusion in C2) the parents/caretakers will answer the following questions using a visual analog scale (VAS) in a diary regardless of patient's age:

- How happy was your child today?
- How active was your child today?

The VAS score will be used as a proxy for assessing the patient's QoL. The scales range from 0 (worst imaginable) to 10 (best imaginable).

At C1, day-4 the parents will receive a diary to answer these questions, which should be brought back to the site at the day of imaging/biopsy at week 6. The site should capture the VAS scores (including dates) from the diary to an accuracy of 1 decimal and enter the data in the eCRF.

9.2.12 Concomitant medication

A concomitant medication is any medication other than the IMP. All concomitant medications until EOT visit must be recorded in the eCRF with the following information:

- Start date
- Stop date of administration or ongoing at trial termination
- Indication/ reason for use
- Dose
- Frequency
- Outcome (for anti-cancer treatments)

Any changes to concomitant medication during the trial will be recorded in the eCRF. All doses of allowed pain relief medication should be recorded as concomitant medication in the eCRF.

During the long-term FU, only therapy related to cancer will be collected.

9.2.13 Unscheduled visits

If any assessments need re-evaluation, an additional response assessment is needed or FU on an (S)AE is required, the patient can be called for an unscheduled visit at the discretion of the investigator. Data pertaining to the clinical trial will be collected as unscheduled visits in the eCRF.

10 ADVERSE EVENTS

10.1 Definitions

10.1.1 Definition of Adverse Event

An AE is any untoward medical occurrence in a patient or clinical trial subject administered a medicinal product and which does not necessarily have a causal relationship with this treatment. An AE can therefore be any unfavorable and/or unintended sign (including an abnormal laboratory finding), symptom or disease temporally associated with the use of a product, whether or not considered related to the product.
An AE includes:

- A clinically significant worsening of a concomitant illness.
- A clinical laboratory adverse event (CLAE): a clinical laboratory abnormality which is clinically significant, i.e., an abnormality that suggests a disease and/or organ toxicity and is of a severity that requires active management. Active management includes active treatment or further investigations, for example change of medicine dose or more frequent FU due to the abnormality.

Throughout the clinical trial a DMC will monitor the patients' safety (section 6.2).

10.1.2 Definition of Serious Adverse Event

Each AE is to be classified by the investigator as serious or non-serious. This classification of the seriousness of the event determines the reporting procedures to be followed. An AE that meets one or more of the following criteria/outcomes is classified as serious:

- Requires inpatient hospitalization or prolongation of existing hospitalization
- Results in persistent or significant disability/incapacity
- Is a congenital anomaly/birth defect
- Medically important
- Results in death
- Is life-threatening

The term "life-threatening" in the definition of "serious" refers to an event in which the patient was at risk of death at the time of the event; it does not refer to an event, which hypothetically might have caused death if it were more severe. Death alone is not considered an AE; it is an outcome of an AE. Reports of death should be accompanied by the corresponding AE term for the event that led to the outcome of death. However, sudden death or death due to unexplainable cause(s) should be reported as an SAE, while FU is pursued to determine the cause.

Elective surgery or other scheduled hospitalization periods that were planned before the patient was included in this trial are not to be considered serious.

Medical and scientific judgment must be exercised in deciding whether an AE is believed to be "medically important". Medical important events may not be immediately life-threatening or result in death or hospitalization but may jeopardize the patient or may require intervention to prevent one of the other outcomes listed in the definition above.

10.1.3 Definition of non-serious AE

A non-serious AE is any AE which does not fulfil the definition of an SAE.

10.1.4 Pre-defined AEs of Special Interest

The following AEs are selected as AEs of Special Interest (serious and non-serious) based on previously experience with anti-GD2 antibodies, GM-CSF and prior exposure to cytotoxic chemotherapy:

- PRES (Posterior Reversible Encephalopathy Syndrome)
- Myelosuppression (Grade 4)
- Hypertension
    - requiring antihypertension therapy *or*
    - which persist at the 90th percentile or higher of BP for age, height and sex requiring additional monitoring after naxitamab infusion according to section 6.7.4.3.
- Adverse events leading to incomplete dosing during a cycle (meaning total planned dose was not given in a cycle)

10.2 Adverse Event Recording

10.2.1 Pre-existing conditions

In this trial, a pre-existing condition (i.e., a disorder present before the AE reporting period started/ signing the Informed Consent Form (ICF) and noted on the medical history/physical examination form) should not be reported as an AE unless the condition worsens or episodes increase in frequency during the AE reporting period.

10.2.2 Procedures

Diagnostic and therapeutic non-invasive and invasive procedures, such as surgery, should not be reported as AEs. A medical condition for which an unscheduled procedure was performed should, however, be reported if it meets the definition of an AE. For example, an acute appendicitis should be reported as the AE and not the appendectomy. Additionally, any procedures planned before entering the trial (e.g., for pre-existing conditions) can be excluded.

10.2.3 Signs and symptoms of the cancer disease

Signs and symptoms which, according to the investigator are expected and well established and known consequences of the indication both in intensity and frequency, should not be reported as AEs or SAEs except for events with a fatal outcome. Any unexpected change in the intensity or frequency should be reported as an AE or SAE as applicable. In addition, all deaths (including death caused by PD) and secondary cancers must be reported as SAE.

10.2.4 Pregnancy

Any pregnancy that occurs during trial participation must be reported to sponsor within 24 hours of learning of its occurrence using the pregnancy form. Pregnant trial patients must be withdrawn from treatment immediately. The pregnancy must be followed up to determine outcome and status of mother and child. The child must be followed at least to the age of one month. Pregnancy complications and elective terminations for medical reasons must be reported as an AE or SAE. Spontaneous abortions must be reported as an SAE.

10.2.5 Severity

The investigator will use the National Cancer Institute (NCI) CTCAE version 4.0 (US Department of Health and Human Services 2010) to describe the severity of the AE, see

below. The grade assigned by the investigator should be the most severe, which occurred during the AE period:

1. Grade 1: Mild; asymptomatic or mild symptoms; clinical or diagnostic observations only; intervention not indicated
2. Grade 2: Moderate; minimal, local or noninvasive intervention indicated; limiting age-appropriate instrumental Activities of Daily Living
    a. Instrumental Activities of Daily Living refer to preparing meals, shopping for groceries or clothes, using the telephone, managing money, etc.
3. Grade 3: Severe or medically significant but not immediately life-threatening; hospitalization or prolongation of hospitalization indicated; disabling; limiting self-care Activities of Daily Living
    a. Self-care Activities of Daily Living refer to bathing, dressing and undressing, feeding self, using the toilet, taking medications, and not bedridden
4. Grade 4: Life-threatening consequences; urgent intervention indicated
5. Grade 5: Death related to AE

10.2.6 Relationship to IMP

The investigator must assess whether the event is related to naxitamab and GM-CSF. A suspected adverse reaction is defined as one in which there is a reasonable possibility that the drug caused the adverse event. Relatedness has to be assessed and reported from the first time the AE is being reported. When assessing the causal relationship of an AE to the IMP, the following should be taken into consideration:

Not related (unlikely)

- Does not follow a reasonable temporal sequence from drug administration
- Is readily explained by the patient's clinical state or by other modes of therapy administered to the patient

Related (possible/probable)

- Follows a reasonable temporal sequence from drug administration
- Abates spontaneously upon discontinuation of the drug (de-challenge) without any curative treatment
- Is confirmed by reappearance of the same reaction on repeat exposure (re-challenge)
- Cannot be reasonably explained by the known characteristics of the patient's clinical state

Alternative etiology should be provided for all AEs assessed as possible related to IMP.

10.2.7 Outcome

The investigator must judge outcome of the AE by the following terms:

- Recovered
- Recovered with sequelae
    - Description of the sequelae should be provided
- Not recovered
- Death
    - Cause of death should be provided

- Unknown
    - Should only be used if the patient is lost to FU

10.3 Side-effects related to naxitamab and GM-CSF

Naxitamab

Please refer to current IB in countries where naxitamab is not approved. In countries where naxitamab is approved please refer to the locally approved label.

Anaphylactoid reactions including bronchospasm has been observed in connection with treatment with naxitamab. Assessment of bronchospasm according to the CTCAE criteria in connection with anaphylactic or anaphylactoid reactions has been difficult as the CTCAE grading 4 is worded as follows: “Life-threatening respiratory or hemodynamic compromise; intubation or urgent intervention indicated”.
Hypotension in connection to anaphylactic reaction and hemodynamic compromise as a result of a life-threatening bronchospasm can be difficult to distinguish. An inhalation with salbutamol or comparable treatments where the patient respond well to treatment is not in itself defining the bronchospasm as grade 4.

GM-CSF

GM-CSF is approved by the health authorities in the United States to help patient’s own immune cells during various diseases. Stimulation of the BM can cause excessive production of both neutrophils (resulting in elevated ANC) and leucocytes. CBC should be done before initiation of GM-CSF (Day -4), before administration of the 500 µg/m^2/day (Day 1), and on Day 3 and 5 in the naxitamab cycle. If ANC > 20 x10^9/L or WBC > 50 x10^9/L, GM-CSF should not be administered until values fall below the above-mentioned thresholds.

GM-CSF is not yet approved in the European Union (EU): please refer to Package Insert GM-CSF (sargramostim).

10.4 Reporting of AEs

Non-Serious Events:

Non-serious AEs must be reported from the day of first IMP administration (GM-CSF dose) until EOT visit. Non-serious AEs occurring between Screening and first treatment must be recorded as Medical History.

Serious Adverse Events:

SAEs must be reported from signing the ICF until EOT visit but for a minimum of 6 weeks after the last IMP administration (GM-CSF dose and/or naxitamab infusion). Hereafter and during the long-term FU period (3 years) only SAEs considered related to IMP or new onset of cancers/autoimmune diseases regardless of causality should be reported.

All Adverse Events:

All events meeting the definition of an AE must be collected in the eCRF. SAEs and Adverse Events of Special Interest (whether serious or non-serious) should be reported both in the eCRF and on the paper Safety Form.

During each contact with the trial site staff, the patient must be asked about AEs, for example by asking: “Have you experienced any problems since the last contact?”

All AEs, observed by the investigator or patient, must be reported by the investigator and evaluated unless specifically excluded in the protocol.

All AEs must be recorded by the investigator on an AE form. The investigator should report the diagnosis, if available. If no diagnosis is available, the investigator should record each sign and symptom as individual AEs using separate AE forms.

Timelines for reporting:

- Initial SAEs (including pre-defined AEs of Special Interest whether serious or not): The paper Safety Form must be reported from site to sponsor within 24 hours of the investigator's first knowledge of the event. The paper Safety Form is to be sent to Y-mAbsy e-mail (or by fax in emergency situations). Please ensure the eCRF AE form is updated in accordance to agreed data entry timelines.
- New FU information available at site must be reported within 24 hours
- FU information requested from Sponsor must be replied to within three working days by using the paper clarification form. Please ensure the eCRF AE form is updated in accordance to agreed data entry timelines.
- If the eCRF is unavailable, the concerned SAE information must be entered when the eCRF becomes available again.

Contact details for reporting:
E-mail: safetymailbox@ymabs.com
Fax: 0045 7879 6060

Sponsor assessment of AE expectedness:
The sponsor assessment of expectedness for naxitamab is performed according to the current version of the IB. The sponsor assessment of expectedness for GM-CSF (sargramostim) is performed according to the current prescribing information.

Reporting of trial product related SUSARs:
Y-mAbs will ensure that all relevant information about Suspected Unexpected Serious Adverse Reactions (SUSARs) is reported to regulatory authorities in accordance to regulatory requirements. Additionally, the DMC will be informed about all SUSARs.

The contract research organizations (CROs) will notify the investigator of SUSARs in accordance with local requirements and International Conference on Harmonization (ICH) Good Clinical Practice (GCP). In addition, the investigator will be informed of any trial-related SAEs that may warrant a change in any trial procedure. The CRO will inform the Institutional Review Boards (IRBs)/ Independent Ethics Committees (IECs) of SUSARs in accordance with local requirement and ICH GCP, unless locally this is an obligation of the investigator.

10.5 Follow-Up on AEs

All non-serious AEs should be followed until they are resolved or until the EOT visit, whichever comes first.

SAEs including AEs of Special Interest should be followed on a regular basis, according to the investigator's clinical judgment, until the event has been resolved or until the investigator can assess it as chronic or stable. This includes follow-up after EOT and Long-term FU.

If an ongoing SAE changes in intensity, relationship to IMP or as new information becomes available and/or known for the event, a FU SAE report form should be completed and sent to the sponsor within 24 hours of the change in SAE assessment.

If the investigator becomes aware of a SAE after the long-term FU period with a suspected causal relationship to the IMP, the investigator should report this SAE within the same timelines as for SAEs during the trial.

11 LABORATORIES

Clinical chemistry and hematology
Please see section 9.2.9 for details on clinical chemistry and hematology.

ADA
ADA response will be assessed by a central laboratory (see lab manual for method description) following a multi-tiered approach.

PK
PK samples will be analyzed by a central laboratory (see lab manual for method description).

Research blood
Research blood will be analyzed by a central laboratory (see lab manual for method description). Please see section 9.2.6.

Imaging and bone marrow
Imaging and BM will be assessed centrally. For bone and BM sampling, please see pathology manual for description and for imaging see imaging manual for description.

12 STATISTICAL METHODS AND DETERMINATION OF SAMPLE SIZE

12.1 General overview

The data will be summarized in tables, as appropriate, showing the number of patients with non-missing data, mean, standard deviation, median, minimum, and maximum for continuous data and showing counts and percentage for categorical data. Data will also be listed as deemed appropriate. All statistical analyses will be performed and data appendices will be created by using SAS (Statistical Analysis Software) version 9.4 or later.

All statistical tests will be two-sided with an α-level of 0.05. A Statistical Analysis Plan (SAP) will be prepared before data base lock. The SAP will describe in detail the analyses presented below. Furthermore, handling and presentation of data including base-line data and other trial assessments will be described in the SAP.

12.2 Populations of interest

Full analysis set
The FAS will include all patients enrolled in the trial cohort 1 who begin an infusion of naxitamab. Cohort 2 will serve subgroup analyses and safety purposes only. The cohorts are defined in section 6.6.
Any exclusions from the FAS deemed reasonable based on ICH-E9 criteria, will be defined and documented in the minutes of the data review meeting prior to database lock.

Per protocol analysis set (PPAS)
The PPAS will include all FAS patients who have no major protocol deviations that are evaluated to affect the overall trial results. Such major protocol deviations can cause exclusion from the PPAS. The precise reasons for and timing of exclusion of patients from the PPAS will be defined and documented in the minutes of the data review meeting prior to the final data base lock.

Safety analysis set (SAF)
The safety analysis set will include all patients enrolled in the trial regardless of cohort who begin an infusion of naxitamab.

12.3 Efficacy analysis

An interim efficacy and safety analysis will occur when the first 37 patients evaluable for objective response assessed by independent central review in FAS cohort 1 have ended treatment and completed EOT visit or complete a response assessment following at least 4 treatment cycles, whichever comes first. The interim efficacy analysis will include centrally assessed as well as investigator assessed ORR and DoR as well as safety including ADA formation on these 37 patients. The interim efficacy analyses will be presented in total and by baseline disease status (primary refractory/incomplete response to salvage treatment) as well as by baseline disease compartment (bone alone, BM alone, or bone and BM). Based on this analysis an interim clinical trial report will be produced.
A final analysis of long-term follow-up including all efficacy endpoints, and safety will be completed after the last evaluable patient has completed follow-up, has withdrawn consent, is lost to follow-up or is dead. Analyses will be done separately for each cohort as well as in total and within each cohort also by baseline disease status and disease compartment. This analysis will be used as the basis for the full clinical trial report.

For the final analysis, the FAS will be used for all efficacy endpoints, for DoR only the sub-group of objective responders is relevant. Additionally, the primary and secondary endpoint analyses will also be performed on the PPAS. In the interim as well as the final analysis, or the proportion of patients obtaining an objective response i.e. the ORR, a two-sided 95% confidence interval will be calculated using the Clopper-Pearson exact methodology. Effectiveness will be concluded if the lower limit of the confidence interval exceeds 20%. Secondary efficacy endpoints of DoR, PFS and OS will be analyzed using Kaplan-Meier methods. The median DoR and median survival times will be estimated, and 95% confidence intervals will be calculated. PFS at 3-years with 95% CI will be estimated. The results will be displayed with survival plots. For PFS and DoR, progression is registered as objective PD until end of centrally assessed response assessment and will for PFS also be supplemented with investigator assessed progression during long-term follow-up.

12.4 Safety analysis

The SAF will be used for the final safety analyses. Analyses will be presented by cohort and in total, as well as by baseline disease status within each cohort. AE data will be listed individually, and the incidence of AEs will be summarized by system organ class and preferred terms within a system organ class.

AEs will be coded using the Medical Dictionary for Regulatory Activities (MedDRA) version 19.0 or later. AEs will be regarded as Treatment-Emergent Adverse Event (TEAEs) if they occur after start of first infusion of naxitamab. Related or possible related AEs are defined as adverse drug reaction (ADRs).

Listings will be made of all AEs by cohort, including non-TEAEs. All TEAEs, ADRs, SAEs and SUSARs will be summarized. The summaries will include number of events, number of patients, and percentage of patients reporting these events and will be tabulated by system organ class and preferred term. TEAEs will also be summarized by severity and by relationship to trial drug.

When calculating the incidence of AEs, each AE will be counted only once for a given patient within a specified system organ class, preferred term and will be done as follows: If the same AE occurs on multiple occasions for a patient, the occurrence with the highest severity and relationship to trial drug will be reported.

Changes in vital signs, hematology, and clinical chemistry parameters from baseline to the end of the trial will be examined. Treatment-emergent changes from normal values to abnormal values in key laboratory parameters will be identified.

12.5 PK analysis and analysis of ADA formation

Serum concentrations of naxitamab will be evaluated. PK parameters including C_{max}, C_{min}, clearance, volume of distribution, AUC, and $t_{1/2}$ will be calculated with non-compartmental analysis and summarized with descriptive statistics. Any additional population PK analysis will be described in a separate analysis plan and report.

ADA formation will be investigated following a multi-tiered approach: A screening-confirmation-titration analysis plus a ligand binding assay to examine a potential neutralizing effect of anti-naxitamab antibodies. Details of the analysis of ADA formation and relationship to response and safety will be described in the SAP.

12.6 Pharmacodynamic analysis

Analyses exploring the relationship between naxitamab PK parameters and the efficacy endpoints will be performed.

12.7 Quality of Life

The QoL information recorded by VAS (see section 9.2.11) will be summarized and plotted over time.

12.8 Interim analysis

Ad-hoc interim analyses may be performed in connection with health authority interactions.

12.9 Subgroup analyses

The efficacy analyses will be repeated for five subgroups:

1. Patients in cohort 2 screened positive for ADA
2. Patients with disease in BM with bone involvement
3. Patients with disease in BM without bone involvement
4. Patients with prior anti-GD-2 therapy
5. Patients with no prior anti-GD-2 therapy

Further subgroups will be described in the SAP.

12.10 Sample size

Assuming an actual ORR of 45%, a sample size of 37 initially ADA-negative patients with no pre-planned radiotherapy (cohort 1), is sufficient to ensure at least 90% power to exclude an ORR of 20% or less at the two-sided 5% level or equivalently to demonstrate that the lower limit of the two-sided 95% exact Clopper-Pearson confidence interval is greater than 20%. Since cohort 2 serves exploratory purposes only, no formal sample size calculations for these will be done.

For long-term follow-up, the meta analysis of the COG trials including almost exclusively high-risk patients (92%), estimates the 4-year PFS in trials after 2009 to 10% +/- 4% (London et al. 2017). Still assuming exponential survival, this extrapolates to a PFS at 3 years of 18% with a lower limit of 12%. Based on this we have conducted a simulation assuming exponential survival with 10,000 repetitions, which shows that if we assume a 23% 3-year PFS in cohort 1, then including 80 evaluable patients will give approximately 80% power to show that the lower bound of the two-sided 95% CI for the 3-year PFS is above 12% i.e. above the extrapolated lower limit of the COG meta analysis. Thus, the aim is to have 80 patients in cohort 1 evaluable for long-term follow-up. The attrition rate is assumed to be very low, so the overall inclusion target for cohort 1 will be set at 85 patients.

12.11 Handling of missing data

Patients with evaluable disease at baseline who cannot be evaluated for response post-baseline will be considered to be non-responders. For the time to event efficacy endpoints of DoR, PFS, and OS, patients who do not have an event will be censored at their date of their last evaluation. Missing safety data will not be imputed.

12.12 Reporting

The full clinical trial report will be generated after all patients have reached EOT and will be complemented with an addendum after the long-term FU period.

13 ETHICS

13.1 Independent Ethics Committee (IEC) or Institutional Review Board (IRB)

This protocol and any accompanying material to be provided to the patient (such as patient information sheets and/or descriptions of the trial used to obtain informed consent) will be submitted by the investigator to an IRB/IEC. Approval from the IRB/IEC must be obtained before starting the trial and should be documented in a letter to the investigator.

It is the responsibility of the investigator or his/her representative to obtain approval of the trial protocol/protocol amendments, the patient information and the informed consent from the IRB/IEC before enrolment of any patient into the trial.

13.2 Ethical conduct of the trial

The trial will be conducted in accordance with the protocol, applicable regulatory requirements, ICH GCP and the ethical principles of the Declaration of Helsinki as adopted by the 18th World Medical Assembly in Helsinki, Finland, in 1964 and subsequent versions.

13.3 Patient information and informed consent

The Principal Investigator (PI) or his/her designee must obtain the written informed consent from each patient, and/or the patients acceptable authorized representative, before any trial related procedures are performed as applicable to local regulations. The written patient information must not be changed without prior discussion with the sponsor and approval by the IRB/IEC. Patient and legal representative (e.g. parent(s) or guardian(s)) must receive full trial information, both verbally and written, before consent is given. A child patient will be informed and included in the conversations with the parents, to the extent the child can understand given his/her age. A patient information sheet will be prepared addressing legal representative(s) and a version especially addressing the adolescent population will also be prepared, as and when applicable to local regulations. The patient information will contain full and adequate verbal and written information regarding the objective and procedures of the trial and the possible benefits and risks involved. This will include any information of possible transfer of biological materials, imaging and other needed for central analysis. The consent shall be given in interest of the child, meaning that he/she is presumed willing to participate. Regardless of legal representative(s) written consent, the participation shall not take place if the patient objects. Objection can also be non-verbal and expressed by the child’s attitude, body language or resistance. Informed consent (parents), and if applicable informed assent (child), must be signed in accordance with local regulations.

If applicable to local regulations: if the child turns 18 during the participation in the trial, a written consent must be obtained from him/her before the trial can continue. Before signing the informed consent, the patient/parents must be given sufficient time to consider the possible participation. Further, each patient must be informed about their right to withdraw from the trial at any time. Parents and patients will also be informed that research participation is voluntary but if they withdraw from the trial, their data will still be used. When the ICF has been signed, the patient/parent(s) receives a copy of the signed form and the original is retained in the investigator site file. A second copy may be kept in the patient’s medical notes. The ICFs must be signed and dated both by the signee and by the person providing the information to the patient/parents. It is recommended to notify the patient’s family doctor of the patient’s consent to participate in the trial.

Any data that is generated within the screening period and is considered SoC per local procedures is permitted to be used for the evaluation of patient eligibility even if the evaluation is performed prior to ICF signature.

13.4 Confidentiality

The investigator must assure that patients' anonymity will be strictly maintained and that their identities are protected from unauthorized parties according to local requirements. The investigator must keep a screening log showing codes, names, and addresses for all patients screened and for all patients enrolled in the trial.

The investigator agrees that the IMPs and all information received from sponsor including but not limited to the IB, this protocol, data, eCRFs, and other trial information, remain the sole and exclusive property of sponsor during the conduct of the trial and thereafter. This information is not to be disclosed to any third party (except employees or agents directly involved in the conduct of the trial or as required by law) without prior written consent from sponsor. The investigator further agrees to take all reasonable precautions to prevent the disclosure by any employee or agent of the trial site to any third party or otherwise into the public domain.

14 MONITORING AND QUALITY ASSURANCE

14.1 Compliance with Good Clinical Practice

The investigator will ensure that this trial is conducted in accordance with the principles of the "Declaration of Helsinki" (as amended in Edinburgh, Tokyo, Venice, Hong Kong, Washington, Seoul, and South Africa), International Conference on Harmonization (ICH) guidelines, or with the laws and regulations of the country in which the research is conducted, whichever affords the greater protection to the trial patient.

14.1.1 Protocol compliance

The investigator is responsible for ensuring the trial is conducted in accordance with the procedures and evaluations described in this protocol.

14.1.2 Training of personnel

A training session in the use of naxitamab is mandatory and will be conducted by personnel from sites with recent naxitamab experience e.g. by a competent clinical research nurse. This training is related to the new site's first patient, first visit only.

Furthermore, training of personnel will be conducted during the site initiation visit. If change of personnel occurs, it is the responsibility of the PI to train new personnel and it should be documented by e.g. completion of a training log form. If the protocol or any trial specific procedures are updated, it is the responsibility of the CRA and PI to ensure documented training of all personnel.

14.2 Monitoring

In accordance with the principles of ICH GCP and the sponsor or its designee's SOPs, monitoring of the trial will be arranged. During the trial, the CRA will have regular contacts with the trial site, including visits to ensure that the trial is conducted and documented properly in compliance with the protocol, ICH GCP, and applicable local regulations. The extent of monitoring will be based on a risk assessment and will be described in a monitoring plan produced by the CRO. The CRA will ensure that accountability of IMPs is performed and will review source documents for verification of consistency with the data recorded in the eCRFs (source data verification). The CRA will also provide information and support to the investigational sites.

The trial sites may also be patient to quality assurance audits by the sponsor or its designee as well as inspection by a regulatory authority. The investigator and other responsible personnel must be available during the monitoring visits, audits and inspections and should devote sufficient time to these processes.

The investigator should provide a curriculum vitae or equivalent documentation of suitability to be responsible for the trial including valid GCP training, a copy of current licensure, and should sign a financial disclosure on conflict of interests. All investigators and other responsible personnel should be listed together with their function in the trial on the signature and delegation list to be filed in the investigator site file.

During these contacts, the monitoring activities will include:

- Drug accountability

- AE identification/review
- Checking and assessing the progress of the trial
- Reviewing trial data collected to date for completeness and accuracy
- Conducting source document verification by reviewing each patient's eCRF against source documents (e.g. medical records, ICF, laboratory result reports, raw data collection forms)
- Identifying any issues and addressing resolutions

These activities will be done in order to verify that the:

- Data are authentic, accurate, and complete
- Safety and rights of the patients are being protected
- Trial is conducted in accordance with the currently approved protocol (and any amendments), GCP, and all applicable regulatory requirements

The investigator will allow the CRA direct access to all relevant documents and allocate his/her time and the time of his/her personnel to the CRA to discuss findings and any relevant issues.
In addition to contacts during the trial, the CRA will contact the site prior to the start of the trial to discuss the protocol and data collection procedures with the site personnel.

14.3 Source Data Verification

14.3.1 Source data

All digital or paper hospital records regarding the treatment of the patient included in the trial are considered source data. The following minimum amount of information should be recorded in the hospital records:

- Clinical trial identification.
- Patient identification.
- Date when patient information was given and when signed informed consent was obtained.
- Diagnosis.
- Fulfilment of inclusion criteria.
- Specification of visit dates, concomitant medication and any (S)AEs.
- Specification of the patient's cessation in the trial (e.g. premature withdrawal).
- Specification of the patient's outcome in the trial.

14.3.2 Direct access to source data/documents

The investigator(s)/institution(s) will permit trial-related monitoring, audits, review and regulatory inspection(s), providing access to source data/hospital records. The CRA verifies that each patient has consented in writing to direct access to the original source data/hospital records by the use of written patient information and signed informed consent. During monitoring, the data recorded in the eCRFs by the investigator will be compared for consistency with the source data/hospital records by the CRA. Any discrepancies of data will be documented and explained in the monitoring reports.

14.3.3 Access to information for monitoring

In accordance with ICH GCP guidelines, the CRA must have direct access to the investigator's source documentation in order to verify the data recorded in the eCRFs for consistency. The CRA is responsible for routine review of the eCRFs at regular intervals

throughout the trial to verify adherence to the protocol and the completeness, consistency, and accuracy of the data being entered into the eCRF. The CRA should have access to any patient records needed to verify the entries on the eCRFs. The investigator agrees to cooperate with the CRA to ensure that any problems detected in the course of these monitoring visits are resolved.

14.3.4 Monitoring of pharmacies

If a pharmacy is needed to be involved at the site, then monitoring of the records kept here will be held to the same standard as described elsewhere in this section.

14.3.5 Access to information for auditing or inspections

Representatives of regulatory authorities or of sponsor may conduct inspections or audits of the clinical trial. If the investigator is notified of an inspection by a regulatory authority, the investigator agrees to notify the medical monitor immediately. The investigator agrees to provide to representatives of a regulatory agency or sponsor access to records, facilities, and personnel for the effective conduct of any inspection or audit.

14.4 Quality Assurance

At its discretion, the sponsor (or designee) may conduct a quality assurance audit of this trial. Auditing procedures of the sponsor (or designee) will be followed in order to comply with GCP guidelines and ensure acceptability of the trial data for registration purposes. If such an audit occurs, the investigator will give the auditor direct access to all relevant documents, and will allocate his/her time and the time of his/her personnel to the auditor as may be required to discuss findings and any relevant issues. Additionally, the trial may be patient an inspection. The investigator must allocate his/her time and the time of his/her personnel to the inspector to discuss findings of any relevant issues.

15 DATA HANDLING AND RECORD KEEPING

15.1 Electronic Case Report Forms

For each patient enrolled, an eCRF must be completed and signed by the PI or sub-investigator (as appropriate) within a reasonable time period after data collection. This also applies to records for those patients who fail to complete the trial. If a patient withdraws from the trial, the reason must be noted in the eCRF. If a patient is withdrawn from the trial because of a treatment-limiting AE, thorough efforts should be made to clearly document the outcome.

15.2 eCRF

An eCRF will be established to collect data in a validated and effective way and in compliance with ICH guidelines including audit trail and a query module. An eCRF is required and should be completed for each included patient. The patient's identity must always remain confidential. All information in the eCRFs should be in English.

The completed original eCRF data are the sole property of the sponsor and should not be made available in any form to third parties (except for authorized representatives of appropriate regulatory authorities) without written permission from the sponsor.

The investigator is responsible for ensuring the accuracy, completeness, legibility and timeliness of the data recorded in the eCRFs. It is expected that data is entered in the eCRF within 5 working days.

15.3 Trial documents at site and record retention

The investigator must maintain adequate and accurate records to enable the conduct of the trial to be fully documented and the trial data to be subsequently verified. These documents should be classified into at least the following two categories: (1) investigator's trial file, and (2) patient clinical source documents. The investigator's trial file will contain the protocol/amendments, and IRB/IEC approval with correspondence, informed consent, drug records, personnel curriculum vitae and authorization forms, and other appropriate documents and correspondence. During the trial the investigator will have full access to the eCRF. After the trial is completed, the investigator will receive a copy of the eCRF on CD-ROM or other appropriate electronic storage device. The investigator is required to complete a source data list, defining where the specific source data can be found (patient record/trial specific patient record).

All clinical trial documents must be retained by the investigator for at least two years after the last approval of a marketing application in an ICH region (i.e. US, EU or Japan) and until there are no pending or contemplated marketing applications in an ICH region; or, if no application is filed or if the application is not approved for such indication, for two years after the investigation is discontinued and regulatory authorities have been notified. Investigators may be required to retain documents longer if required by applicable regulatory requirements, by local regulations, or by an agreement with sponsor. The investigator must notify sponsor before destroying any clinical trial records. Should the investigator wish to assign the trial records to another party or move them to another location, sponsor must be notified in advance.

If the investigator cannot guarantee this archiving requirement at the trial site for any or all of the documents, special arrangements must be made between the investigator and sponsor to store these in sealed containers outside of the site so that they can be returned sealed to the investigator in case of a regulatory audit. When source documents are required for the continued care of the patient, appropriate copies should be made for storage outside of the site. Biological samples will be discarded after the analysis has been completed and no later than at the finalization of the full clinical trial report.

15.4 Data management

All data, except laboratory data, will be collected using an eCRF compliant with 21 CFR Part 11 regulation. Part of the laboratory data will be collected via a central laboratory and the other part by local laboratories. Data management will be performed in accordance with applicable standards and data cleaning procedures. Only authorized access to the eCRF will be possible using encrypted username and password. Roles in the system are given according to functions. All tasks performed in the eCRF are logged in an audit trail. The eCRF will contain validation checks to maintain an ongoing quality check of data entered. All data validation will be performed as part of the system.

The investigator will approve the data using an electronic signature and thereby confirm the accuracy of the data recorded. Medical History and AEs will be coded using the MedDRA dictionary. Concomitant medication will be coded using the World Health Organization Drug dictionary.

16 REPORTING AND COMMUNICATION OF RESULTS

16.1 Publication

Data collected in this trial are the property of the sponsor, who agrees to communicate and make available for public disclosure the results of the clinical trial regardless of outcome. Public disclosure means publication in scientific journals, submission of abstract(s) for scientific meetings, and other types of disclosure (e.g. via ClinicalTrials.gov). Co-authorship with investigators will comply with International Committee of Medical Journal Editors rules (ICMJE n.d.) regarding:

- Substantial contributions to the conception or design of the work; or the acquisition, analysis, or interpretation of data for the work; AND
- Drafting the work or revising it critically for important intellectual content; AND
- Final approval of the version to be published; AND
- Agreement to be accountable for all aspects of the work in ensuring that questions related to the accuracy or integrity of any part of the work are appropriately investigated and resolved.

A predefined publication committee will decide on a) whether, where, and when to publish, b) manuscript authorship for the entire clinical trial, and c) authorship of potential additional manuscripts based on sub-trials. All manuscripts relating to sub-trials will state that they are sub-trials and cite the main publication.

16.2 Use of information

Sponsor will make one main publication from the clinical trial and all other publications should come afterwards and refer to the main clinical trial.

All information not previously published concerning the IMPs, including patent applications, manufacturing processes, basic scientific data, clinical trial data and results, etc., is considered confidential and remains the sole property of the sponsor. The investigator agrees to use this information only in connection with this trial and will not use it for other purposes without written permission from the sponsor.

No such communication, presentation, or publication will include sponsor’s confidential information. All presentation and publications will be governed by the publication committee. Proposed publication(s) or presentation(s) along with the respective scientific journal or presentation forum should be provided to the sponsor at least 30 days prior to submission of the publication or presentation. Authors will comply with sponsor’s request to delete references to its confidential information (other than the trial results) in any paper or presentation and also agree to withhold publication or presentation for an additional 60 days to enable application for patent if deemed necessary.

17 INSURANCE

Insurance and liability will comply with applicable laws and GCP.

18 CHANGES AND DEVIATIONS TO THE PROTOCOL

Protocol modifications, except those intended to reduce immediate risk to trial patients, may only be made by the sponsor. Protocol modifications will follow local requirements for submission to the competent authorities and IRB/IECs. Approval must be obtained before changes can be implemented. Any protocol or other deviations that occur during the trial will be documented and reported to the sponsor. Some deviations may be reported to the appropriate regulatory authority. No change in trial procedures shall be made without the mutual agreement of the investigator and the sponsor (except where necessary to eliminate an immediate hazard to patients).

19 PREMATURE TERMINATION OF THE TRIAL

This trial may be terminated by the sponsor. The trial may also be terminated prematurely at any time when agreed to by both investigators and the sponsor as being in the best interest of patients and justified by either medical or ethical grounds. In terminating the trial, sponsor and investigators will ensure that adequate consideration is given to the protection of the patient's interest.

19.1 Criteria for putting patient enrolment on hold

In the following situations enrolment will be put on hold temporarily until the DMC feedback on continuation of the trial has been received:

- If an event, alone or in combination with other safety information, is judged by either Sponsor or DMC Chairman to warrant an acute DMC meeting. This could be an unexpected Serious Adverse Reaction (SARs), or an increased frequency or severity of an already known AE and could be one of the events mentioned in section 6.7.3.
  or
- If one or more patients experience a related Grade 5 event (death) before EOT visit

If one of the above-mentioned situations is met, the DMC will review the safety information and recommend how to proceed.

Patients enrolled before the decision to put the trial enrolment on hold will continue in the trial, but further treatment will be postponed until Sponsor has received the DMC Chairman's recommendation for these patients.

19.2 Criteria for termination of the trial

The sponsor reserves the right to discontinue the trial prior to inclusion of the intended number of patients but intends to exercise this right only for valid scientific or administrative reasons. After such a decision, all delivered unused investigational products and other trial-related materials must be collected without delay and all eCRFs must be completed as far as possible.

The trial could be prematurely discontinued in the following situations (examples):

- New findings about the IMPs that significantly worsens the benefit/risk ratio.
- Compliance with the trial protocol proves difficult.
- Recruitment of eligible patients is far too low or slow.
- Level of investigator, sponsor or patient compliance becomes unacceptable.
- Critical changes are observed in sponsor or trial site personnel, administrative or scientific standards.
- The DMC recommends discontinuation.

20 REFERENCES

Albert, D., J. Dunham, S. Khan, J. Stansberry, S. Kolasinski, D. Tsai, S. Pullman-Mooar, F. Barnack, C. Striebich, R. J. Looney, E. T. Prak, R. Kimberly, Y. Zhang, and R. Eisenberg. 2008. 'Variability in the biological response to anti-CD20 B cell depletion in systemic lupus erythaematosus', *Ann Rheum Dis*, 67: 1724-31.

Bagatell, R., W. B. London, L. M. Wagner, S. D. Voss, C. F. Stewart, J. M. Maris, C. Kretschmar, and S. L. Cohn. 2011. 'Phase II study of irinotecan and temozolomide in children with relapsed or refractory neuroblastoma: a Children's Oncology Group study', *J Clin Oncol*, 29: 208-13.

Basta, N. O., G. C. Halliday, G. Makin, J. Birch, R. Feltbower, N. Bown, M. Elliott, L. Moreno, G. Barone, A. D. Pearson, P. W. James, D. A. Tweddle, and R. J. McNally. 2016. 'Factors associated with recurrence and survival length following relapse in patients with neuroblastoma', *Br J Cancer*, 115: 1048-57.

Brierley, J., J. A. Carcillo, K. Choong, T. Cornell, A. Decaen, A. Deymann, A. Doctor, A. Davis, J. Duff, M. A. Dugas, A. Duncan, B. Evans, J. Feldman, K. Felmet, G. Fisher, L. Frankel, H. Jeffries, B. Greenwald, J. Gutierrez, M. Hall, Y. Y. Han, J. Hanson, J. Hazelzet, L. Hernan, J. Kiff, N. Kissoon, A. Kon, J. Irazuzta, J. Irazusta, J. Lin, A. Lorts, M. Mariscalco, R. Mehta, S. Nadel, T. Nguyen, C. Nicholson, M. Peters, R. Okhuysen-Cawley, T. Poulton, M. Relves, A. Rodriguez, R. Rozenfeld, E. Schnitzler, T. Shanley, S. Kache, S. Skache, P. Skippen, A. Torres, B. von Dessauer, J. Weingarten, T. Yeh, A. Zaritsky, B. Stojadinovic, J. Zimmerman, and A. Zuckerberg. 2009. 'Clinical practice parameters for hemodynamic support of pediatric and neonatal septic shock: 2007 update from the American College of Critical Care Medicine', *Crit Care Med*, 37: 666-88.

Chen, S., T. Caragine, N. K. Cheung, and S. Tomlinson. 2000. 'CD59 expressed on a tumor cell surface modulates decay-accelerating factor expression and enhances tumor growth in a rat model of human neuroblastoma', *Cancer Res*, 60: 3013-8.

Cheung, I. Y., K. Hsu, and N. K. Cheung. 2012. 'Activation of Peripheral-Blood Granulocytes Is Strongly Correlated With Patient Outcome After Immunotherapy With Anti-GD2 Monoclonal Antibody and Granulocyte-Macrophage Colony-Stimulating Factor', *J Clin Oncol*, 30: 426-32.

Cheung, I. Y., B. H. Kushner, S. Modak, E. M. Basu, S. S. Roberts, and N. V. Cheung. 2017. 'Phase I trial of anti-GD2 monoclonal antibody hu3F8 plus GM-CSF: Impact of body weight, immunogenicity and anti-GD2 response on pharmacokinetics and survival', *Oncoimmunology*, 6: e1358331.

Cheung, N. K., I. Y. Cheung, K. Kramer, S. Modak, D. Kuk, N. Pandit-Taskar, E. Chamberlain, I. Ostrovnaya, and B. H. Kushner. 2014. 'Key role for myeloid cells: phase II results of anti-G(D2) antibody 3F8 plus granulocyte-macrophage colony-stimulating factor for chemoresistant osteomedullary neuroblastoma', *Int J Cancer*, 135: 2199-205.

Cheung, N. K., I. Y. Cheung, B. H. Kushner, I. Ostrovnaya, E. Chamberlain, K. Kramer, and S. Modak. 2012. 'Murine anti-GD2 monoclonal antibody 3F8 combined with granulocyte-macrophage colony-stimulating factor and 13-cis-retinoic acid in high-risk patients with stage 4 neuroblastoma in first remission', *J Clin Oncol*, 30: 3264-70.

Cheung, N. K., H. Lazarus, F. D. Miraldi, C. R. Abramowsky, S. Kallick, U. M. Saarinen, T. Spitzer, S. E. Strandjord, P. F. Coccia, and N. A. Berger. 1987. 'Ganglioside GD2

specific monoclonal antibody 3F8: a phase I study in patients with neuroblastoma and malignant melanoma', *J Clin Oncol*, 5: 1430-40.

Cheung, N. K., R. Sowers, A. J. Vickers, I. Y. Cheung, B. H. Kushner, and R. Gorlick. 2006. 'FCGR2A polymorphism is correlated with clinical outcome after immunotherapy of neuroblastoma with anti-GD2 antibody and granulocyte macrophage colony-stimulating factor', *J Clin Oncol*, 24: 2885-90.

Cheung, N. K., E. I. Walter, W. H. Smith-Mensah, W. D. Ratnoff, M. L. Tykocinski, and M. E. Medof. 1988. 'Decay-accelerating factor protects human tumor cells from complement-mediated cytotoxicity in vitro', *J Clin Invest*, 81: 1122-8.

Cohn, S. L., A. D. Pearson, W. B. London, T. Monclair, P. F. Ambros, G. M. Brodeur, A. Faldum, B. Hero, T. Iehara, D. Machin, V. Mosseri, T. Simon, A. Garaventa, V. Castel, K. K. Matthay, and Inrg Task Force. 2009. 'The International Neuroblastoma Risk Group (INRG) classification system: an INRG Task Force report', *J Clin Oncol*, 27: 289-97.

Di Giannatale, A., N. Dias-Gastellier, A. Devos, K. Mc Hugh, A. Boubaker, F. Courbon, A. Verschuur, S. Ducassoul, K. Malekzadeh, M. Casanova, L. Amoroso, P. Chastagner, C. M. Zwaan, C. Munzer, I. Aerts, J. Landman-Parker, R. Riccardi, M. C. Le Deley, B. Geoerger, and H. Rubie. 2014. 'Phase II study of temozolomide in combination with topotecan (TOTEM) in relapsed or refractory neuroblastoma: a European Innovative Therapies for Children with Cancer-SIOP-European Neuroblastoma study', *Eur J Cancer*, 50: 170-7.

Franks, L. M., A. Bollen, R. C. Seeger, D. O. Stram, and K. K. Matthay. 1997. 'Neuroblastoma in adults and adolescents: an indolent course with poor survival', *Cancer*, 79: 2028-35.

Hoeller, C., O. Michielin, P. A. Ascierto, Z. Szabo, and C. U. Blank. 2016. 'Systematic review of the use of granulocyte-macrophage colony-stimulating factor in patients with advanced melanoma', *Cancer Immunol Immunother*, 65: 1015-34.

Kushner, B. H., I. Y. Cheung, K. Kramer, S. Modak, and N. K. Cheung. 2007. 'High-dose cyclophosphamide inhibition of humoral immune response to murine monoclonal antibody 3F8 in neuroblastoma patients: broad implications for immunotherapy', *Pediatr Blood Cancer*, 48: 430-4.

Kushner, B. H., K. Kramer, M. P. LaQuaglia, S. Modak, and N. K. Cheung. 2003. 'Neuroblastoma in adolescents and adults: the Memorial Sloan-Kettering experience', *Med Pediatr Oncol*, 41: 508-15.

Kushner, B.H., and N.K. Cheung. 1992. 'Absolute requirement of CD11/CD18 adhesion molecules, FcRII and the phosphatidylinositol-linked FcRIII for monoclonal antibody-mediated neutrophil antihuman tumor cytotoxicity', *Blood*, 79: 1484-90.

Kushner BH, Kramer K, LaQuaglia MP, Modak S, Yataghene K, and Cheung N-KV. 2004. 'Reduction from seven to five cycles of intensive induction chemotherapy in children with high-risk neuroblastoma.', *J Clin Oncol*, 22: 4888-92.

LaQuaglia MP, Kushner BH, Su W, Heller G, Kramer K, Abramson S, Rosen N, Wolden S, and Cheung N-KV:. 2004. 'Impact of gross total resection on local control and survival in high-risk neuroblastoma.', *J Pediatr Surg*, 39: 412-17.

London, W. B., R. Bagatell, B. J. Weigel, E. Fox, D. Guo, C. Van Ryn, A. Naranjo, and J. R. Park. 2017. 'Historical time to disease progression and progression-free survival in

patients with recurrent/refractory neuroblastoma treated in the modern era on Children's Oncology Group early-phase trials', *Cancer*, 123: 4914-23.

London, W. B., V. Castel, T. Monclair, P. F. Ambros, A. D. Pearson, S. L. Cohn, F. Berthold, A. Nakagawara, R. L. Ladenstein, T. Iehara, and K. K. Matthay. 2011. 'Clinical and biologic features predictive of survival after relapse of neuroblastoma: a report from the International Neuroblastoma Risk Group project', *J Clin Oncol*, 29: 3286-92.

Mackall, C. L. 2000. 'T-cell immunodeficiency following cytotoxic antineoplastic therapy: a review', *Stem Cells*, 18: 10-8.

Mackall, C. L., D. Stein, T. A. Fleisher, M. R. Brown, F. T. Hakim, C. V. Bare, S. F. Leitman, E. J. Read, C. S. Carter, L. H. Wexler, and R. E. Gress. 2000. 'Prolonged CD4 depletion after sequential autologous peripheral blood progenitor cell infusions in children and young adults', *Blood*, 96: 754-62.

Maris, J. M. 2010. 'Recent advances in neuroblastoma', *N Engl J Med*, 362: 2202-11.

Maris, J. M., M. D. Hogarty, R. Bagatell, and S. L. Cohn. 2007. 'Neuroblastoma', *Lancet*, 369: 2106-20.

Metelitsa, L. S., S. D. Gillies, M. Super, H. Shimada, C. P. Reynolds, and R. C. Seeger. 2002. 'Antidisialoganglioside/granulocyte macrophage-colony-stimulating factor fusion protein facilitates neutrophil antibody-dependent cellular cytotoxicity and depends on FcgammaRII (CD32) and Mac-1 (CD11b/CD18) for enhanced effector cell adhesion and azurophil granule exocytosis', *Blood*, 99: 4166-73.

Modak, S., and N. K. Cheung. 2007. 'Disialoganglioside directed immunotherapy of neuroblastoma', *Cancer Invest*, 25: 67-77.

Modak, S., B. H. Kushner, E. Basu, S. S. Roberts, and N. V. Cheung. 2017. 'Combination of bevacizumab, irinotecan, and temozolomide for refractory or relapsed neuroblastoma: Results of a phase II study', *Pediatr Blood Cancer*: e26448.

Moreno, L., H. Rubie, A. Varo, M. C. Le Deley, L. Amoroso, A. Chevance, A. Garaventa, M. Gambart, F. Bautista, D. Valteau-Couanet, B. Geoerger, G. Vassal, X. Paoletti, and A. D. Pearson. 2017. 'Outcome of children with relapsed or refractory neuroblastoma: A meta-analysis of ITCC/SIOPEN European phase II clinical trials', *Pediatr Blood Cancer*, 64: 25-31.

Morgenstern, D. A., W. B. London, D. Stephens, S. L. Volchenboum, T. Simon, A. Nakagawara, H. Shimada, G. Schleiermacher, K. K. Matthay, S. L. Cohn, A. D. Pearson, and M. S. Irwin. 2016. 'Prognostic significance of pattern and burden of metastatic disease in patients with stage 4 neuroblastoma: A study from the International Neuroblastoma Risk Group database', *Eur J Cancer*, 65: 1-10.

Mueller, S., and K. K. Matthay. 2009. 'Neuroblastoma: biology and staging', *Curr Oncol Rep*, 11: 431-8.

Mujoo, K., T. J. Kipps, H. M. Yang, D. A. Cheresh, U. Wargalla, D. J. Sander, and R. A. Reisfeld. 1989. 'Functional properties and effect on growth suppression of human neuroblastoma tumors by isotype switch variants of monoclonal antiganglioside GD2 antibody 14.18', *Cancer Res*, 49: 2857-61.

Park, J. R., R. Bagatell, S. L. Cohn, A. D. Pearson, J. G. Villablanca, F. Berthold, S. Burchill, A. Boubaker, K. McHugh, J. G. Nuchtern, W. B. London, N. L. Seibel, O. W. Lindwasser, J. M. Maris, P. Brock, G. Schleiermacher, R. Ladenstein, K. K. Matthay,

and D. Valteau-Couanet. 2017. 'Revisions to the International Neuroblastoma Response Criteria: A Consensus Statement From the National Cancer Institute Clinical Trials Planning Meeting', *J Clin Oncol*, 35: 2580-87.

Pearson, A. D., A. W. Craft, C. R. Pinkerton, S. T. Meller, and M. M. Reid. 1992. 'High-dose rapid schedule chemotherapy for disseminated neuroblastoma', *Eur J Cancer*, 28A: 1654-9.

Petrella, T., I. Quirt, S. Verma, A. E. Haynes, M. Charette, K. Bak, and Care Melanoma Disease Site Group of Cancer Care Ontario's Program in Evidence-based. 2007. 'Single-agent interleukin-2 in the treatment of metastatic melanoma: a systematic review', *Cancer Treat Rev*, 33: 484-96.

Saarinen, U. M., P. F. Coccia, S. L. Gerson, R. Pelley, and N. K. Cheung. 1985. 'Eradication of neuroblastoma cells in vitro by monoclonal antibody and human complement: method for purging autologous bone marrow', *Cancer Res*, 45: 5969-75.

Schwartz, G. J., A. Muñoz, M. F. Schneider, R. H. Mak, F. Kaskel, B. A. Warady, and S. L. Furth. 2009. 'New equations to estimate GFR in children with CKD', *J Am Soc Nephrol*, 20: 629-37.

Tong, W., M. Maira, M. Gagnon, and H. U. Saragovi. 2015. 'Ligands Binding to Cell Surface Ganglioside GD2 Cause Src-Dependent Activation of N-Methyl-D-Aspartate Receptor Signaling and Changes in Cellular Morphology', *PLoS One*, 10: e0134255.

US Department of Health and Human Services. 2010. 'Common Terminology Criteria for Adverse Events.'. http://evs.nci.nih.gov/ftp1/CTCAE/CTCAE_4.03_2010-06-14_QuickReference_5x7.pdf.

Wilson, J. S., J. E. Gains, V. Moroz, K. Wheatley, and M. N. Gaze. 2014. 'A systematic review of 131I-meta iodobenzylguanidine molecular radiotherapy for neuroblastoma', *Eur J Cancer*, 50: 801-15.

Yu, A. L., A. L. Gilman, M. F. Ozkaynak, W. B. London, S. G. Kreissman, H. X. Chen, M. Smith, B. Anderson, J. G. Villablanca, K. K. Matthay, H. Shimada, S. A. Grupp, R. Seeger, C. P. Reynolds, A. Buxton, R. A. Reisfeld, S. D. Gillies, S. L. Cohn, J. M. Maris, P. M. Sondel, and Group Children's Oncology. 2010. 'Anti-GD2 antibody with GM-CSF, interleukin-2, and isotretinoin for neuroblastoma', *N Engl J Med*, 363: 1324-34.

Appendix 1 Contact list of Clinical Trial Team Members

Sponsor
Y-mAbs Therapeutics A/S
Agern Allé 11
DK-2970 Hoersholm
Denmark

[redacted]
Senior Clinical Project Manager
E-mail: [redacted]
Phone: [redacted]

Medical Monitor/Expert
Y-mAbs Therapeutics A/S
Agern Allé 11
DK-2970 Hoersholm
Denmark

[redacted]
Medical Director
E-mail: [redacted]
Phone: [redacted]

Safety/Pharmacovigilance
Y-mAbs Global Clinical Drug Safety
Agern Allé 11
DK-2970 Hoersholm
Denmark

E-mail: safetymailbox@ymabs.com
Fax: 0045 7879 6060

Clinical Trial Supply Coordination
Packaging and labelling
Y-mAbs Therapeutics A/S
Agern Allé 11
DK-2970 Hoersholm
Denmark

[redacted]
Senior Supply Chain Manager
E-mail: [redacted] /
[redacted]

Appendix 2 Creatinine-based 2009 revised Bedside Schwartz Equation

eGFR = 0.413 x (height/Scr) (Schwartz et al. 2009)

Height is expressed in centimeters

Abbreviations / Units

eGFR = mL/min/1.73 m^2

Scr (standardized serum creatinine) = mg/dL

Appendix 3 Wong-Baker FACES® Pain Rating Scale

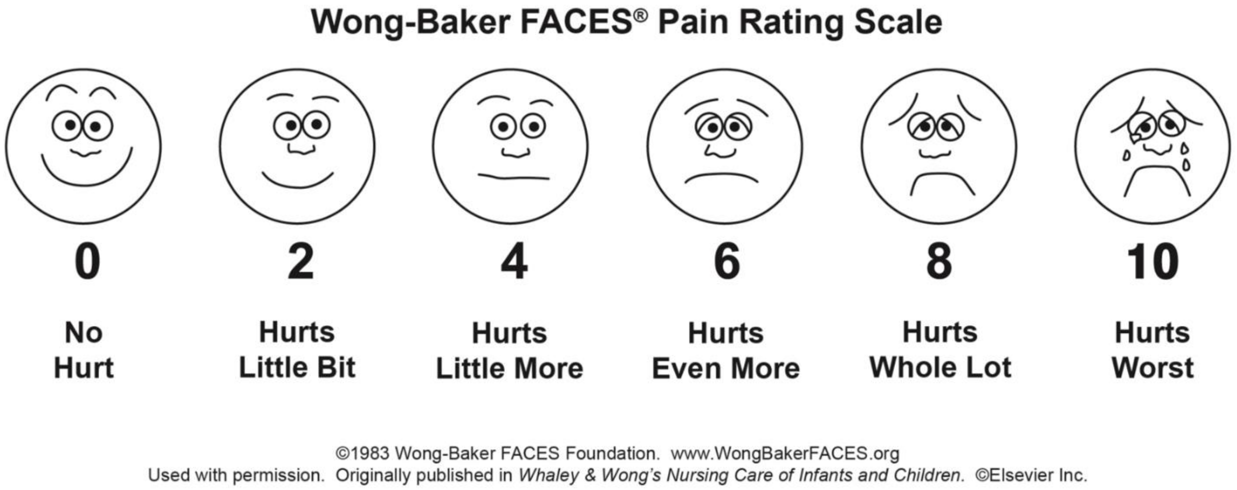

Instructions

Prior to the naxitamab infusion, the Wong Baker FACES® Pain Rating Scale, should be presented and explained to the patient, by informing the participant that each face on the scale represents a person who has no pain, some pain or a lot of pain. The Wong Baker FACES® Pain Rating Scale should be used (a) prior to commencement of naxitamab, (b) worst pain during infusion to be assessed at end of infusion) and, (c) pain 15 min after end of infusion (d) prior to discharge immediately after every naxitamab infusion in all cycles. The staff who will be asking the participant to rate his/her pain should explain that:

- Face 0 does not hurt at all.
- Face 2 hurts just a little bit.
- Face 4 hurts a little bit more.
- Face 6 hurts even more.
- Face 8 hurts a whole lot.
- Face 10 hurts as much as you can imagine, although you don´t have to be crying to have this worst pain.

The rating should be done by asking the participant to choose the face that best depicts the pain they are experiencing.

Appendix 4 FLACC Behavioral Scale

FLACC Behavioral Scale

Categories	Scoring		
	0	1	2
Face	No particular expression or smile	Occasional grimace or frown, withdrawn, disinterested	Frequent to constant frown, clenched jaw, quivering chin
Legs	Normal position or relaxed	Uneasy, restless, tense	Kicking, or legs drawn up
Activity	Lying quietly, normal position, moves easily	Squirming, shifting back and forth, tense	Arched, rigid, or jerking
Cry	No cry (awake or asleep)	Moans or whimpers, occasional complaint	Crying steadily, screams or sobs, frequent complaints
Consolability	Content, relaxed	Reassured by occasional touching, hugging, or being talked to, distractable	Difficult to console or comfort

Each of the five categories (F) Face; (L) Legs; (A) Activity; (C) Cry; (C) Consolability is scored from 0-2, which results in a total score between zero and ten.

Copyright© 2002, The Regents of the University of Michigan. All Rights Reserved.

FLACC Behavioral Pain Scale

Patients who are awake: Observe for at least 1-2 min. Observe legs and body uncovered. Reposition patient or observe activity, assess body for tenseness and tone. Initiate consoling interventions if needed

Patients who are asleep: Observe for at least 2 min or longer. Observe body and legs uncovered. If possible reposition the patient. Touch the body and assess for tenseness and tone.

Face

Score 0 point if patient has a relaxed face, eye contact and interest in surroundings

Score 1 point if patient has a worried look to face, with eyebrows lowered, eyes, partially closed, cheeks raised, mouth pursed

Score 2 points if patient has deep furrows in the forehead, with closed eyes, open mouth and deep lines around nose/lips

Legs

Score 0 points if patient has usual tone and motion to limbs (legs and arms)

Score 1 point if patient has increase tone, rigidity, tense, intermittent flexion/extension of limbs

Score 2 points if patient has hyper tonicity, legs pulled tight, exaggerated flexion/extension of limbs, tremors

Activity
Score 0 points if patient moves easily and freely, normal activity/restrictions
Score 1 point if patient shifts positions, hesitant to move, guarding, tense torso, pressure on body part
Score 2 points if patient is in fixed position, rocking, side-to-side head movement, rubbing body part

Cry
Score 0 points if patient has no cry/moan awake or asleep
Score 1 point if patient has occasional moans, cries, whimpers, sighs
Score 2 points if patient has frequent/continuous moans, cries, grunts

Consolability
Score 0 points if patient is calm and does not require consoling
Score 1 point if patient responds to comfort by touch or talk in - 1 min
Score 2 points if patient require constant consoling or is unconsoled after an extended time
Whenever feasible, behavioral measurement of pain should be used in conjunction with self-report. When self-report is not possible, interpretation of pain behaviors and decision making regarding treatment of pain requires careful consideration of the context in which the pain behaviors were observed.
Each category is scored on the 0-2 scale which results in a total score of 0-10Assessment of Behavioral Score:
0 = Relaxed and comfortable
1-3 = Mild discomfort
4-6 = Moderate pain
7-10 = Severe discomfort/pain

Copyright© 2002, The Regents of the University of Michigan. All Rights Reserved.

Signature Page for CLIN-000004 v12.0

Approval	[redacted]<br>19-Jan-2021 10:57:29 GMT+0000

Approval	[redacted]<br>19-Jan-2021 11:11:24 GMT+0000

Approval	[redacted]<br>19-Jan-2021 13:03:21 GMT+0000

Signature Page for CLIN-000004 v12.0

Statistical Analysis Plan

A Pivotal Phase 2 Trial of Antibody Naxitamab (hu3F8) and Granulocyte-Macrophage Colony Stimulating Factor (GM-CSF) in High-Risk Neuroblastoma Patients with Primary Refractory Disease or Incomplete Response to Salvage Treatment in Bone and/or Bone Marrow

Clinical Trial ID: 201

Preplanned Interim Analysis using December 31st 2021 data cut-off

Version 5.0, 30MAR2022

**Investigational Medicinal Product:**	Monoclonal antibody Naxitamab (hu3F8) and Sargramostim (yeast-derived human recombinant GM-CSF)
**Indication:**	High-risk neuroblastoma (NB) with primary refractory disease or incomplete response to salvage treatment in bone and/or bone marrow (BM).
**Phase:**	2
**Date:**	30Mar2022

Protocol version and date: Protocol version 12.0, 14Jan2021

Confidentiality Statement

The information in this document is confidential and is not to
be disclosed without written consent of Y-mAbs Therapeutics A/S

Approval of Statistical Analysis Plan

Statistical Analysis Plan Author		
Y-mAbs Therapeutics A/S<br>[redacted]<br>Senior Project Statistician	Please refer to the e-signature page	
Print Name:	Signature:	Date:

Table of Contents

List of abbreviations and definitions of terms 7

1 Introduction 9
1.1 Trial objectives for preplanned interim analysis 9
1.1.1 Primary objective 9
1.1.2 Secondary objectives 9
1.1.3 Other objectives 10
1.2 Trial design 10
1.2.1 Background 10
1.2.2 General design considerations 11
1.3 Sample size for preplanned interim analysis 12
1.4 Randomization 13

2 Endpoints 14
2.1 Primary endpoint 14
2.2 Secondary endpoints 14
2.3 Other endpoints 14

3 Analysis populations 16
3.1 Full analysis set (FAS) 16
3.2 Per-Protocol Analysis Set (PPAS) 16
3.3 Safety analysis set (SAF) 16

4 Data handling specifications 17
4.1 Technical specifications for display 17
4.2 Handling of missing data 17
4.3 Visits and visit windows 18
4.4 Baseline 18
4.5 Subgroup analyses 18

5 Statistical methods 21
5.1 General specifications 21
5.2 Subject enrolment and disposition 21
5.2.1 Protocol Deviations 21
5.3 Demographics and baseline characteristics 22
5.4 Medical and surgical history 22
5.5 Prior neuroblastoma therapy 22
5.6 Prior and concomitant medications 24
5.7 Exposure and compliance 25
5.7.1 Exposure to naxitamab 25
5.7.2 Exposure to GM-CSF 26
5.7.3 Dose interruptions and delays 26

6 Evaluations of Endpoints 27
6.1 Analysis of primary efficacy endpoint 27
6.1.1 Sensitivity analyses 28
6.2 Analysis of secondary efficacy endpoints 28

6.3 Analysis of other efficacy endpoints 28
6.4 Subgroup analyses 29

7 Pharmacokinetics and anti-drug antibody (ADA) formation 30
7.1 Naxitamab pharmacokinetics 30
7.2 Anti-drug antibody formation 30

8 Safety Endpoints 31
8.1 Adverse Events 31
8.2 Clinical laboratory evaluations 33
8.3 Vital signs 35
8.4 Electrocardiogram 35
8.5 Pain assessments 35
8.6 Performance assessments 36
8.7 Hospitalization days 36

9 Changes to planned analyses 37

10 SAP version history 38

Appendix 1 Templates for Tables and figures 40

List of Tables

Table 1 Definitions of subgroups 18
Table 2 Change log 38

List of Figures

Figure 1-1. One cycle of naxitamab + GM-CSF 12
Figure 1-2. Treatment and response assessment schedule 12

List of abbreviations and definitions of terms

Abbreviations

ADA	anti-drug antibody
ADCC	antibody-dependent cellular cytotoxicity
ADR	adverse drug reaction
CR	complete response
AE	adverse event
ALT	alanine aminotransferase
ANC	absolute neutrophil count
AST	aspartate aminotransferase
ATC	Anatomical Therapeutic Chemical
BM	bone marrow
BUN	blood urea nitrogen
ch14.18	chimeric antibody 14.18 (dinutuximab, trade name: Unituxin)
CBC	complete blood count
CR	complete response
CT	computed tomography
CTCAE	Common Toxicity Criteria for Adverse Events
DNA	deoxyribonucleic acid
DoR	duration of response
eCRF	electronic case report form
ECG	electrocardiogram
EOT	end of treatment
FAS	full analysis set
FDG PET	^{18}F-Fluorodeoxyglucose Positron Emission Tomography
FLACC	Face, Legs, Arms, Cry, Consolability
GM-CSF	granulocyte-macrophage colony stimulating factor
ICH	International Conference on Harmonization
IMP	investigational medicinal product
INRC	International Neuroblastoma Response Criteria
IV	intravenous
LDH	lactate dehydrogenase
mAb	monoclonal antibody
MedDRA	Medical Dictionary for Regulatory Activities
MIBG	metaiodobenzylguanidine
MRI	magnetic resonance imaging
mu3F8	murine 3F8
NB	neuroblastoma
NCI	National Cancer Institute

ORR	overall response rate
PD	progressive disease
PK	pharmacokinetic
PT	preferred term
QoL	quality of life
SAE	serious adverse event
SAF	safety analysis set
SAP	statistical analysis plan
SAR	serious adverse reaction
TEAE	treatment emergent adverse event
WHO DDE	World Health Organization Drug Dictionary Extended

Definitions of terms

Baseline	Baseline is defined as the last available measurement before first IMP treatment

1 Introduction

Trial 201 is a pivotal Phase 2 single arm, open-label, multi-site, international clinical trial evaluating the efficacy, safety, and pharmacokinetics of the humanized monoclonal antibody, naxitamab, for the treatment of high-risk neuroblastoma (NB) patients with primary refractory disease or incomplete response to salvage treatment in bone and/or bone marrow (BM).

The sources for this statistical analysis plan (SAP) include the Trial 201 Protocol version 12.0 and the corresponding annotated electronic case report form (eCRF) version 12.0 for Trial 201.

The proposed International Nonproprietary Name for the drug compound is naxitamab (United States Adopted Names accepted). The drug compound has also been described as hu3F8 and this name will thus also occur in documents for naxitamab.

The purpose and scope of this SAP is to address the preplanned interim analysis with data cut-off December 31st 2021. All data and clinical assessments made up to December 31st, 2021, for subjects receiving first dose of naxitamab no later than December 31st 2021 are included, even if they were entered into the eCRF database at a later date. The Full Analysis Set will comprise all subjects receiving first dose of naxitamab no later than November 19th 2021.

1.1 Trial objectives for preplanned interim analysis

The primary and secondary objectives described below correspond to the specification of the preplanned interim analysis in section 12.3 in the protocol.

1.1.1 Primary objective

The preplanned interim analysis has the following primary objective:

1. To evaluate the centrally assessed objective response rate (ORR) to naxitamab + GM-CSF.

1.1.2 Secondary objectives

The preplanned interim analysis has the following secondary objectives:

1. To evaluate the safety of naxitamab + GM-CSF
2. To evaluate duration of response (DoR) to naxitamab + GM-CSF
3. To evaluate the investigator assessed ORR to naxitamab + GM-CSF
4. To investigate the formation of anti-drug antibodies (ADAs)
5. To evaluate the safety of naxitamab + GM-CSF in patients with positive ADA at trial entry

1.1.3 Other objectives

The preplanned interim analysis has the following other objectives, which are falling outside the scope of the defined objectives in the specification of the preplanned interim analysis within section 12.3 in the protocol:

1. To evaluate the CR rate with naxitamab + GM-CSF
2. To evaluate PFS with naxitamab + GM-CSF
3. To evaluate OS with naxitamab + GM-CSF
4. To evaluate the rate of centrally assessed confirmed objective response (OR) with naxitamab + GM-CSF
5. To evaluate the pharmacokinetics (PK) of naxitamab
6. To evaluate quality of life (QoL)

1.2 Trial design

1.2.1 Background

Neuroblastoma is a rare cancer but is the most common extracranial solid tumor of childhood. More than 50 percent of patients have metastatic disease (stage 4) and typically in the BM (Maris et al. 2007; Mueller and Matthay 2009). Intensive induction chemotherapy, aggressive surgery, and radiotherapy have improved remission rates of high-risk NB in young patients (Pearson et al. 1992; LaQuaglia MP et al. 2004; Kushner BH et al. 2004). Treatments have been less successful in adolescents and adults, where NB is typically chemoresistant (Franks et al. 1997; Kushner et al. 2003; Maris 2010).).

Despite intensive frontline multimodal therapy, at least 50% of high-risk NB patients have persistence of disease or develop PD. After relapse, these patients have poor outcomes with 5-year OS of ~10% (London et al. 2011). For both primary refractory patients and patients having incomplete response to salvage treatment, salvage chemotherapy regimens using 1-3 agents have shown disappointing ORR of 10-35% (London et al. 2011; Bagatell et al. 2011; Di Giannatale et al. 2014; Moreno et al. 2017; Modak et al. 2017), and the same holds for salvage with ^{131}I-MIBG therapy (Wilson et al. 2014). Promising clinical results in Trial 12-230 were achieved in patients with primary refractory disease and patients with incomplete response to salvage treatment in bone and/or BM (secondary refractory disease).

Various strategies have been applied to induce or augment immune-mediated attack against cancer. However, few clinical trials have successfully used monoclonal antibody (mAb)-

mediated immunotherapy against solid tumors in children or adults, especially with antibody-dependent cellular cytotoxicity (ADCC) as the key underlying immune mechanism. Building on the favorable results of Memorial Sloan Kettering-developed murine 3F8 (mu3F8), naxitamab has several potential advantages over mu3F8 and the chimeric antibody ch14.18 (dinutuximab). These advantages include: (1) low immunogenicity allowing repeat treatments over years, (2) improved ADCC potency, (3) longer serum half-life reducing the necessity of daily injections, (4) reduction of pain side effects. This pivotal Phase 2 trial builds on the highly encouraging recent findings from earlier trials demonstrating the major anti-NB activity of naxitamab + GM-CSF.

1.2.2 General design considerations

This single-arm, open-label, non-randomized trial includes a screening period followed by treatment and observation periods. Patients will be enrolled until at least 80 patients in cohort 1 are evaluable for objective response and PFS. A preplanned interim analysis will be conducted when at least 37 patients in cohort 1 are evaluable for objective response.

Patients with high-risk NB, defined as MYCN-amplified NB stages 2/3/4/4s of any age and MYCN-nonamplified stage 4 patients > 18 months of age, meeting the inclusion and not violating the exclusion criteria will be enrolled into two separate cohorts depending on ADA status and the need for pre-planned radiotherapy at screening.

The two cohorts are:

1. Patients screened negative for ADA, and with no pre-planned radiotherapy for metastatic lesions, and with evaluable disease at baseline in bone and/or bone marrow.

2. Patients screened positive for ADA, or with pre-planned radiotherapy for metastatic lesions, or without evaluable disease in bone and/or bone marrow at baseline.

Evaluable disease at baseline will be as assessed by independent review. Cohort 1 is the cohort of primary interest. Cohort 2 serves subgroup analyses purposes.

Each patient will receive trial drug for a maximum of 101 weeks after the first naxitamab infusion. After the end of treatment (EOT), each patient will enter a long-term follow up for 3 years.

Each investigational cycle is started with five days (days -4 to 0) of GM-CSF administered at 250 μg/m^2/day in advance of the start of naxitamab infusion. GM-CSF is thereafter administered at 500 μg/m^2/day on days 1 to 5. Naxitamab is infused over ~30-60 min at 3 mg/kg/day on days 1, 3, and 5 for a total dose of 9 mg/kg per cycle as shown in Figure 1-1.

Figure 1-1. One cycle of naxitamab + GM-CSF

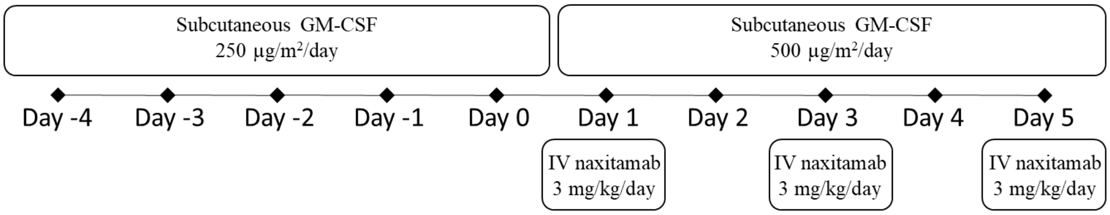

Treatment cycles are repeated every 4 weeks (±1 week) until CR or PR followed by 5 additional cycles every 4 weeks (±1 week). Subsequent cycles are repeated every 8 weeks (±2 weeks) through 101 weeks from first infusion (Figure 1-2) at the discretion of the investigator. The patients will undergo response assessments as shown in Figure 1-2.

Figure 1-2. Treatment and response assessment schedule

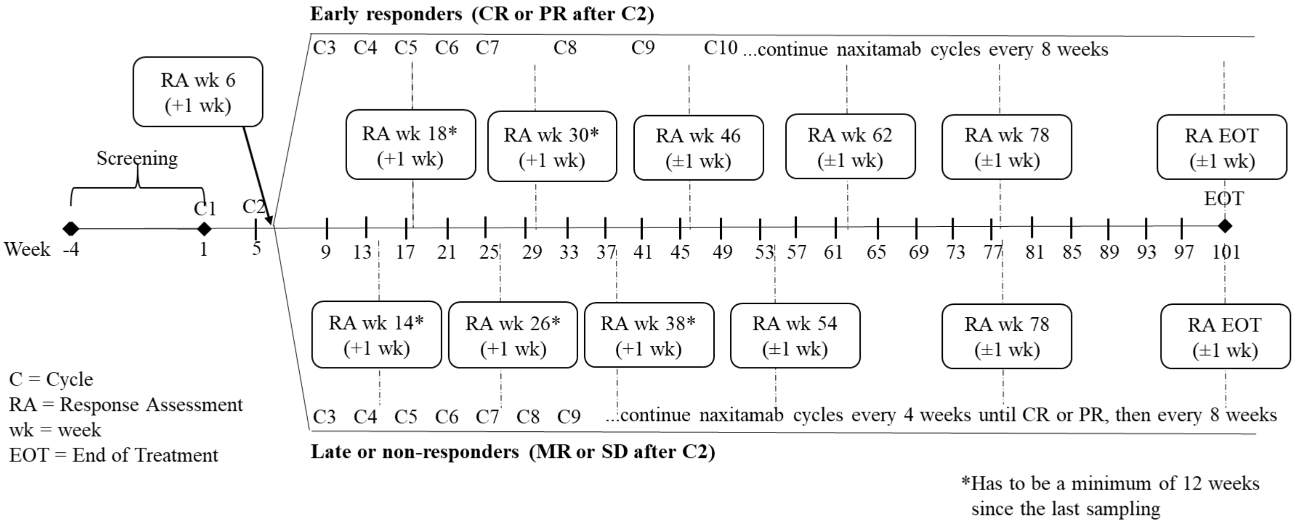

During and after naxitamab treatment, safety and efficacy will be assessed as described in the flow chart in the protocol with assessments for each treatment cycle followed by long-term follow up for up to 3 years after end of treatment.

1.3 Sample size for preplanned interim analysis

Sample size was calculated to ensure that the preplanned interim analysis for ORR in cohort 1 patients is well powered. Assuming an actual ORR of 45%, a sample size of 37 patients screened negative for ADA, with no pre-planned radiotherapy and with evaluable disease in bone and/or BM at baseline (cohort 1), is sufficient to ensure at least 90% power to exclude an ORR of 20% or less at the two-sided 5% level or equivalently to demonstrate that the lower limit of the two-

sided 95% exact Clopper-Pearson confidence interval is greater than 20%. Since cohort 2 serve exploratory purposes only, no formal sample size calculations for these will be done.

1.4 Randomization

This is a single arm, open-label trial and no randomization will be performed.

2 Endpoints

The following endpoints are defined for the preplanned interim analysis.

2.1 Primary endpoint

The preplanned interim analysis has the following primary endpoint:

1. ORR during the naxitamab treatment period, centrally assessed according to the INRC

2.2 Secondary endpoints

The preplanned interim analysis has the following secondary endpoints:

1. Safety will be evaluated by the incidence of AEs and SAEs graded according to CTCAE, version 4.0
2. DoR, defined as the time from first objective response (CR or PR) to PD; data will be censored at the date of last disease evaluation before new anti-NB treatment
3. ORR, during the naxitamab treatment period, investigator assessed according to INRC
4. Assessment of ADA formation
5. In patients with positive ADA at trial inclusion, safety will be evaluated by the incidence of AEs and SAEs graded according to CTCAE, version 4.0

2.3 Other endpoints

The preplanned interim analysis has the following other endpoints next to the endpoints defined in the specification of the preplanned interim analysis within section 12.3 in the protocol:

1. Complete response (CR) rate, during the naxitamab treatment period, centrally assessed according to the INRC
2. PFS, defined as the time from the 1st infusion of naxitamab until PD or death, whichever comes first; data will be censored at the date of last disease evaluation before new anti-NB treatment or last assessment during long-term follow-up, whichever comes first
3. OS, defined as the time from the first infusion of naxitamab until death; data will be censored at last date known to be alive during long-term follow-up.

4. Confirmed ORR, during the naxitamab treatment period, response (CR and PR) centrally assessed according to the INRC and confirmation information evaluated according to algorithm inspired by RECIST v1.1

5. Assessment of the PK of naxitamab

6. Happiness and activity levels measured over time assessed by caretaker

3 Analysis populations

The following analysis sets are defined in accordance with the ICH E9 Guidance.

3.1 Full analysis set (FAS)

The FAS will include all patients enrolled in the trial who begin an infusion of naxitamab. Cohort 1 within the FAS will be used for the primary efficacy analysis. Cohort 2 will serve subgroup analyses and safety purposes only.

Exclusions from the FAS may be made if deemed reasonable based on ICH-E9 guidance criteria (cf. Section 5.2.1 in ICH E9 guidance), e.g. in order to avoid bias in the assessment of the primary efficacy endpoint, patients without evaluable disease at screening according to eligibility criteria in section 6.7.1 in the protocol, as assessed by central review, will be excluded from the FAS.

Any exclusions from the FAS will be defined and documented.

For the preplanned interim analysis the FAS will include all subjects receiving first dose of naxitamab no later than November 19th 2021 to allow for a response assessment at the planned week 6 visit to occur no later than December 31st 2021.

3.2 Per-Protocol Analysis Set (PPAS)

The per-protocol analysis set will not be used for the interim analysis.

3.3 Safety analysis set (SAF)

The safety analysis set (SAF) will include all patients enrolled in the trial regardless of cohort who begin an infusion of naxitamab.

For the preplanned interim analysis the SAF will include all subjects receiving first dose of naxitamab no later than December 31st 2021.

4 Data handling specifications

4.1 Technical specifications for display

All tables and listings will be produced by using SAS®, Version 9.4 or higher. All analysis displays will be created by SAS®, Version 9.4 or higher. Displays will be produced by using the Courier New 8-point font. Headers will also be in Courier New 8-point font.

All displays are intended to be printed as landscape on 8.5×11-in paper. The top and bottom margins will be 0.50 inch, and the left and right margins will be 0.75 inch. Header and footer information should not invade the specified margins.

Relative to the number of digits after the decimal in the original data, summary statistics will have the following number of digits after the decimal:

- Mean, median, percentiles, and confidence interval: one more digit
- Standard deviation and standard error: two more digits
- Minimum, maximum, and range: same number of digits

Summary statistics will not exceed four digits after the decimal. For laboratory data converted to SI units, the above rule will apply to the converted data. For PK/Dosimetry data these rules may be applied with modifications in relation to the magnitude of the measurements.

Percentages will be displayed with one digit after the decimal. P-values will be displayed to 4 decimal places.

4.2 Handling of missing data

For the primary and secondary efficacy endpoints based on response to treatment, patients for whom a response cannot be determined will be considered non-responders. For the duration of response (DoR), patients who do not have an event will be censored at the date of their last evaluation/contact. If partial dates are present, a conservative approach will be used. If partial dates are present for efficacy time to event endpoints, missing months and/or days will be imputed as January and the first day of the month, respectively.

AEs with a (partial) missing start date or time that leads to ambiguity in whether the AE is treatment-emergent will be considered treatment-emergent with start date imputed to start date of treatment. Other missing AE dates will be handled conservatively i.e., for a partial start date missing month will be imputed as January and missing day will be imputed as the first. Whereas for a partial end date missing month will be imputed as December and missing day will be imputed as last day of the month.

Duration of AEs will be calculated including imputed dates. Treatment-emergent AEs with missing causality will be considered related to IMP. It is unlikely that other missing safety data will occur in the targeted patient population, but in case it happens the data will not be imputed.

4.3 Visits and visit windows

Data presented by visit will be summarized and listed by using the recorded nominal visit values in the eCRF regardless of the actual study day on which a value was collected. Unscheduled visits will not be summarized in tables in which data are summarized by visit but will be presented in data listings by date.

4.4 Baseline

For all analyses, the baseline value or baseline covariate will be defined as the last non-missing value prior to the first dose of naxitamab. eCRFs will be used to capture trial results and data.

4.5 Subgroup analyses

Selected subgroups among those defined in Table 1 below will be used for summaries of baseline characteristics and endpoints.

Table 1 Definitions of subgroups

Seq	Subgroup variable	Subgroups and codings, if any	Specification
1	Age group at baseline	1 = (0 - <2),<br>2 = (2 - <6),<br>3 = (6 - <12),<br>4 = (12 - <18) and<br>5 = (age >=18 years)	Derived from actual age (years) at baseline
2	Sex	1 = Female<br>2 = Male	As recorded in eCRF
3	Body weight group	1 = (<20 kg),<br>2 = (20-<50 kg),<br>3 = (>=50 kg)	Derived from actual body weight (kg) at baseline
4	Disease status at baseline	1 = Primary refractory,<br>2 = Incomplete response to salvage treatment	As recorded in eCRF
5	Baseline disease location according to investigator	1 = Bone,<br>2 = Bone marrow,<br>3 = Bone and bone marrow	As recorded in eCRF

Seq	Subgroup variable	Subgroups and codings, if any	Specification
6	Baseline disease location according to independent review	1 = Bone,<br>2 = Bone marrow,<br>3 = Bone and bone marrow	
7	Response compartment	1 = Bone (i.e. disease in "Bone" or "Bone and bone marrow")<br>2 = Bone marrow (i.e. disease in "Bone marrow" or "Bone and bone marrow")	As recorded in eCRF
8	Curie score	1 = (0-2),<br>2 = (>=3)	As recorded in eCRF
9	Prior anti-GD2 therapy	1 = Yes,<br>2 = No	As recorded in eCRF
10	ADA category	1 =Negative at all assessments,<br>2 = Positive at any time	As recorded in eCRF
11	Systemic steroid use as pre-medication	1 = Yes,<br>2 = No	Systemic steroids are identified as ATC code class 2= H02. Premedications are identified as ("Pre-infusion medication"-box in CRF=checked) or (Reason for use = "Premedication" and start time before start of naxitamab infusion).
12	Use of IV saline as pre-medication	1 = Yes,<br>2 = No	Specific details to be specified separately later
13	Responders	1 = (best response CR or PR),<br>2 = (best response not in (CR PR))	

Seq	Subgroup variable	Subgroups and codings, if any	Specification
14	Patients with and w/o CR	1 = Patients who during trial obtained Complete Response vs.<br>2 = Patients who obtained Partial Response (PR), Minor Response (MR), Stable Disease (SD), Progressive Disease (PD) or Not Evaluable (NE) only	

5 Statistical methods

5.1 General specifications

Summary tables of data will be provided as appropriate. For continuous variables, tables will show the total number of patients, number of patients with non-missing data, mean, standard deviation, median, 25th and 75th percentiles (Q1, Q3), minimum, and maximum. For categorical variables, tables will show the counts and percentages. The denominator of percentages will be the number of subjects in the analysis set, except for those variables collected by trial visit or scheduled time point, in which case the denominator of percentages will be the number of subjects with a non-missing value at the visit or the scheduled time point.

In summaries of adverse events, the number of events (E) will also be presented. The denominator of percentages will be the number of patients, except for those variables collected by trial visit or scheduled time point, in which case the denominator of percentages will be the number of patients with a non-missing value at the visit or the scheduled time point.

Data listings will also be provided.

For all analyses, the baseline value or baseline covariate will be defined as the last non-missing value prior to the first dose of naxitamab. eCRFs will be used to capture trial results and data.

Unless otherwise stated, all reference to responders or response assessment will be according to independent review.

All statistical tests will be two-sided and evaluated at a 5% significance level, unless otherwise specified.

5.2 Subject enrolment and disposition

Patient enrollment, inclusion in analysis populations, number of patients discontinuing trial treatment(s) or trial before trial completion, the trial stage during which they discontinued and the reasons for premature discontinuation of treatment and withdrawal from trial will be summarized with frequencies and, where appropriate, percentages of enrolled patients. Data will also be listed. A patient is considered enrolled as of first trial drug (GM-CSF) exposure.

5.2.1 Protocol Deviations

Any protocol or other deviations that occur during the trial will be documented and reported to the sponsor. Deviations will be categorized as major or minor by the trial monitor and recorded in the eCRF. Allocation to the major or minor category will be reviewed and confirmed prior to database lock. Major protocol deviations will be listed.

5.3 Demographics and baseline characteristics

Demographic and baseline characteristics will be presented for the FAS and SAF analysis sets.

Demographic and baseline characteristic data will include age (based on date of informed consent), sex, ethnicity, race, height, weight, body surface area, performance score (Lansky or Karnofsky). Age will be calculated as the difference between the patient's date of informed consent and the date of birth and presented in years, with one decimal. If the birth date is missing, completely or in part, the age as recorded in the CRF will be used instead. All demographic and baseline characteristic data will be summarized for the FAS and SAF analysis sets and will be listed.

Demographics and baseline characteristics will be presented for the subgroups specified in Table 1 in section 4.5 above.

Baseline disease characteristics will include neuroblastoma location, current neuroblastoma status, age at diagnosis in years and months, time from initial diagnosis to trial entry, MYCN amplification status, International Neuroblastoma Staging System stage at diagnosis, DNA diploidy, International Neuroblastoma Pathology Classification, prognostic group (favorable vs. unfavorable), time to first relapse, time from latest relapse to first dose of naxitamab, site of relapse, number of relapses, and lactase dehydrogenase level at time of diagnosis will be summarized for the FAS and SAF and will be listed.

The baseline disease characteristics will also be presented for the subgroups specified in Table 1 in section 4.5 above.

Descriptive summary statistics of (adjudicated) baseline Curie score will be presented for patients who obtained CR vs. patients who obtained PR, MR, SD, PD or NE.

Descriptive summary statistics of percentage of patients with disease in bone marrow within (adjudicated) categories 0-5% (not applicable to cohort 1), 5-20%, >20% bone marrow infiltration at baseline will be presented for patients who obtained CR vs. patients who obtained PR, MR, SD, PD or NE.

5.4 Medical and surgical history

Non-neuroblastoma medical history data will be coded using Medical Dictionary for Regulatory Activities (MedDRA), version 20.1 and summarized by system organ class and preferred term and listed for the SAF. Medical history related to NB disease will be summarized and listed separately.

5.5 Prior neuroblastoma therapy

Summaries of the following will be presented: the number of patients with prior surgery, prior chemotherapy, prior radiation, prior anti-GD2 therapy, prior stem cell infusions, prior

hematopoietic progenitor cell boosts (top-ups), prior donor-lymphocyte-infusions, response to latest prior therapy, and time from latest treatment to trial entry.

The following information will be summarized and listed:

- Best response to most recent prior systemic therapy
- Duration of response of most recent prior therapy
- Most recent systemic therapy prior to enrollment

- Did patient receive anti GD-2 treatment at any time prior to enrollment?
    - If yes, type of anti-GD2 treatment
    - Response to prior anti-GD2 treatment

- Did patient receive surgery?
    - At primary site:
        - Curative
        - Palliative
        - with complete resection
        - Without complete resection
        - Not Applicable
    - At metastatic site:
        - With complete resection
        - Without complete resection
        - Not Applicable

- Did patient receive ASCT?
    - Single
    - Tandem

- Did patient receive stem cell infusions,
- Did patient receive hematopoietic progenitor cell boosts (top-ups)
- Did patient receive donor-lymphocyte-infusions
- Did patient receive radiation
    - Treatment was part of:
        - Induction therapy
        - Consolidation therapy
        - Salvage therapy (treatment of active disease/relapse)
        - Other
    - Target for radiation
        - Primary site
        - Metastatic soft tissue Site
        - Metastatic bone site (listing only, see specification below)
    - Number of sites

    - Total radiation dose per cycle (Gy)
    - Number of fractions within a cycle
    - Number of cycles
- Did the patient receive 131-MIBG
    - Total 131I-MIBG dose per cycle
    - Number of cycles

For radiation to metastatic bone site a listing will provide details of the metastatic bone site.

The prior neuroblastoma therapy will be also presented for the following subgroups (see Table 1 in Section 4.5 for specifications): 4, 5, 6, 9, 13 and 14.

The above will be summarized for both the FAS and SAF as feasible and corresponding listings will be prepared.

5.6 Prior and concomitant medications

Prior and concomitant medication information is collected from screening through the follow-up period. All prior and concomitant medications will be coded using World Health Organization Drug Dictionary Extended (WHO DDE) September 2017 and will be summarized with frequencies and percentages of patients who received medications by Anatomical Therapeutic Chemical (ATC) class and preferred name (preferred term) and will be provided in a listing.

Pre-medications (as per tick-box for pre-medications in eCRF) will be summarized in total and by timing (administered 0-15 minutes; 15-30; and >30 minutes prior to naxitamab infusion).

Opioid use (number of doses, total dose (mg) and dose/kg (mg/kg)) will be calculated for each patient and summarized with descriptive statistics by Days 1 to 6 and All (where All=from infusion day 1 to day before first infusion in subsequent cycle) for each cycle (cf. Table Template 8). In the summary of total doses and dose/kg, opioids dosages will first be converted to their i.v. morphine dose equivalent. Furthermore, summaries of opioids differentiating between opioid info captured as i) pre-medication, ii) IV Opioid eCRF (IV opioids administered for infusion-related pain from 2 hours prior and 4 hours post infusion to be captured on IV Opioid page) and iii) opioid administered outside this window will be prepared.

Ketamine use within infusion days will be summarized in the same way (cf. Table Template 8).

The calculated opioid and ketamine use will be listed.

If a medication has an end date and end time that occur before first naxitamab dose date and time, that medication will be considered a prior medication. If a medication has a start date and start time that occur before first naxitamab dose date and time but an end date and end time that occur after first dose date and time, that medication will be considered prior and concomitant. If a medication has a start date, start time, end date, and end time that occur after first dose date and time, that medication will be considered concomitant. Should a missing start date, start time, end

date, or end time lead to ambiguity in whether a medication is prior or concomitant, the medication will be considered concomitant.

5.7 Exposure and compliance

Summaries of exposure will be presented for the FAS and SAF. The compliance results will be presented for the SAF by cohort.

5.7.1 Exposure to naxitamab

Naxitamab administration takes place on Days 1, 3 and 5 of each treatment cycle. The following will be summarized and listed: the number and proportion of patients receiving naxitamab by cycle, the number of cycles initiated, number of cycles completed, cumulative total exposure (mg), mean naxitamab dose (mg/kg) by cycle, number of infusions, and per-infusion exposure to naxitamab (mg/kg). The data will be listed.

The number and proportion of:

- infusions done in an outpatient setting
- infusions done in an inpatient setting
- Infusions requiring hospitalization
- infusions completed as planned (completed as planned=yes if actual dose (mg) infused is within 99%-101% of planned dose (mg))
- infusions completed as planned using intervals with a maximum 100% and minimums at 90%, 91%, …, 98% of planned dose

will be summarized by infusion and overall, as well as listed.

Duration of infusion per infusion number will be summarized both as a continuous variable as well as a categorized variable for all infusions. The following categories will be used: <30 min, 30 min-<45 min, 45 min-<60 min; 1-<2 hours, 2-<4 hours, 4-<6 hours and >=6 hours intervals.

Duration of infusion will also be presented in graphs for the first, second and third infusion (cf. Figure Template 7 in Appendix 1).

Follow-up time in the trial as well as the total number of patient months will be summarized. The total number of patient months is calculated as follows:

$$\mathrm{Totalpatientmonths} = \sum_{j=1}^{n} \mathrm{fu}_j,$$

where

n denotes the number of patients,

fu_j denotes the individual follow-up time for patient *j*,

and follow-up time is defined as the time from the first dose to the last assessment, in months.

Exposure time, defined as time from first to last naxitamab infusion in days, in the trial will be summarized, in total and for subgroups defined above, displaying the number of patients in the treatment phase for 1, 2, 4 weeks, and 2, 4, 6, 9, 12,15, 18, 21, and 24 months.

5.7.2 Exposure to GM-CSF

The following will be summarized: the number of patients receiving GM-CSF by cycle, the number of cycles administered, cumulative total exposure (mcg), mean dose days -4 to 0 (mcg/m^2) and mean dose days 1-5(mcg/m^2), total and mean number of doses, and per-infusion exposure to GM-CSF split into day -4 to 0 & day 1-5 (each split presented as mcg/m^2). The data will be listed.

5.7.3 Dose interruptions and delays

Dose modifications and interruptions or changes in infusion rates will be summarized and listed. The summaries will present the number and % of patients for whom there was a: change in infusion rate, infusion interruption, duration of interruption, interruption or rate change due to an AE, overall ad by cycle. If several changes or interruptions of infusions were made these will be presented (as available).

Proportion and 95% confidence intervals of patients with infusion rate change or interruption will be presented in a bar-chart (cf. Figure Template 8 in Appendix 1).

To further investigate protocol schedule compliance and dose delays, the following will be presented.

The within cycles compliance will be presented for as follows:

- Patients not receiving all 3 infusions in a cycle,
- Doses not completed (completed= yes if the actual dose administered (mg) >=99% of the planned dose) on an infusion day in total and by infusion
- Cycles not completed (completed=yes if the patient received dose on 3 infusion days in the same cycle) within 10 days

will be presented in a summary by cycle and overall.

The following will be presented in a listing:

- the nominal protocol scheduled administration study day (number of days from first dose),
- the nominal protocol scheduled administration weekday (e.g. Monday),

- actual administration study day (actual number of days from first dose)
- actual weekday,
- the difference (in days) between the nominal protocol scheduled and actual administration study days

Patients not following the Monday, Wednesday, Friday schedule will be marked in the listing.

The between-cycles compliance, as measured by weeks from start (infusion day 1) to start (infusion day 1) of two cycles, will be listed.

6 Evaluations of Endpoints

The FAS will be the primary analysis set for all efficacy analyses. Summaries will be presented by subgroups, see Section 6.3. Any statistical tests will be two-sided and performed on the 5% significance level.

6.1 Analysis of primary efficacy endpoint

The primary efficacy endpoint is ORR, during the naxitamab treatment period, centrally assessed according to the International Neuroblastoma Response Criteria (INRC) (Park, 2017).

The response evaluation, including Complete Response (CR) and Duration of Response (DoR), will be obtained from the independent, central review of images and pathology.

Response will be defined as achieving an overall best response of PR of CR as defined by INRC. The proportion of patients achieving response and a two-sided 95% confidence interval will be calculated using Clopper-Pearson exact methodology. Efficacy will be concluded if the lower limit of the 95% exact confidence interval for ORR for the FAS cohort 1 exceeds 20%.

The following SAS code can be used to produce the analysis:

```
proc freq;
        tables aval / nocum binomial (exact level='1' p=0.2);
        exact binomial;
run;
```

where ‘aval’ is binary variable indicating response.

Also, proportions and corresponding 95% confidence intervals of patients per INRC response category (CR, PR, MR, SD and Progressive Disease (PD)) will be presented.

Patients who are not evaluable post baseline will be considered as non-responders, and hence included in the denominators in the response rate calculations.

The primary analysis will be based on Cohort 1.

6.1.1 Sensitivity analyses

There are no sensitivity analyses defined for the primary endpoint within the preplanned interim analysis.

6.2 Analysis of secondary efficacy endpoints

DoR, duration of response, is defined as the time from first objective response to PD. DoR will be censored at the date of:

- last available and evaluable disease evaluation as per central review
- start of new anti-NB treatment.

New anti-NB treatment:

- Any additional systemic anti-cancer therapy (including stem cell infusions, hematopoietic progenitor cell boosts (top-ups) and donor-lymphocyte-infusions)
- Any radiotherapy not pre-defined at baseline
- Any other anticancer therapy outside this protocol

DoR will be analyzed using Kaplan-Meier methods. The first, second (median) and third quartile of the DoR distribution will be estimated and corresponding 95% confidence intervals will be presented. The Kaplan-Meier estimated distribution of DoR will be presented in a plot for Cohort 1. The number of patients, number of events and number of censored observations will be presented. Also, the reason for censoring (last response assessment or prohibited medication administered) will be summarized.

<u>Time (in weeks), as well as the number of cycles, to response</u> *i.e.* time from start of treatment to first PR or CR, to first CR, as well as to first occurrence of the best overall response will be summarized, for responders, by descriptive statistics and by Kaplan-Meier estimates with corresponding plots.

The investigator assessed versions of ORR and DoR will be analysed similar to the centrally assessed endpoints.

6.3 Analysis of other efficacy endpoints

The CR rate is defined as the proportion of patients experiencing a CR according to centrally assessed INRC criteria during the treatment period. The complete response rate will be analyzed using same methods as the primary efficacy endpoint, but no formal testing will be employed.

The endpoint for confirmed response is defined as the rate of centrally assessed confirmed OR (CR or PR) with naxitamab + GM-CSF. This endpoint will be evaluated similarly to the primary endpoint (albeit without formal testing), except that confirmed response is derived based on INRC 2017 for response assessment and using the algorithm specified in Table 3 in RECIST v1.1 for combining response assessments into evaluations of confirmed response.

PFS is defined as the time from the first infusion of naxitamab until PD or death, whichever comes first; data will be censored at the date of last disease evaluation before new anti-NB treatment or last assessment during long-term follow-up, whichever comes first. PFS will be analyzed using Kaplan-Meier methodology to estimate the survival curve and PFS at 3 years. No formal testing will be employed.

OS is defined as the time from the first infusion of naxitamab until death; data will be censored at last date known to be alive during long-term follow-up. OS will be analyzed using Kaplan-Meier methodology to estimate the survival curve and OS at 3 years. No formal testing will be employed.

6.4 Subgroup analyses

ORR, complete response rate and DoR will be summarized and presented for the subgroups specified in Table 1 in section 4.5, except for subgroups number 2, 13 and 14.

7 Pharmacokinetics and anti-drug antibody (ADA) formation

7.1 Naxitamab pharmacokinetics

The analyses of naxitamab PK are addressed in a separate PK analysis plan and report.

7.2 Anti-drug antibody formation

ADA results will be presented for the SAF.

For the analysis of Anti-Drug Antibody (ADA) formation, blood samples will be collected during screening, during cycle 1 at pre-infusion and Day 12 and within one hour before the first infusion of naxitamab for all subsequent treatment cycles, and at EOT.

ADA titers will be listed and summarized with descriptive statistics by baseline and post-baseline. The frequency and percentage of patients who are positive for ADA will also be calculated and presented by trial visit. For the patients with positive ADA result, the titer will be summarized by visit. For the patients with positive ADA result, presence of neutralizing antibodies will be summarized by visit. For the patients with positive ADA result, time to first positive ADA titer will be summarized as well as a patient data listing of all ADA results will be presented.

8 Safety Endpoints

The SAF will be used for all safety analyses. Analyses will be presented by cohort and in total.

8.1 Adverse Events

All adverse events (AEs) will be coded using the Medical Dictionary for Regulatory Activities (MedDRA) Version 20.1.

All adverse events will be collected from the day of first IMP administration (GM-CSF dose) and in addition serious adverse events will be collected from time of signed informed consent. Adverse events will be graded per the Common Toxicity Criteria for Adverse Events (CTCAE), version 4.0. Adverse events will be regarded as Treatment-Emergent Adverse Event (TEAEs) if they occur after start of first IMP administration (GM-CSF dose). AEs which are recorded by investigator as being related to naxitamab or GM-CSF treatment will be considered as adverse drug reaction (ADRs), hereafter named treatment-related TEAEs. Date and time for AE occurrences will not be imputed. AEs with a missing start date or time that leads to ambiguity as to whether the AE is treatment-emergent will be considered treatment-emergent.

TEAEs will be presented in an overview table (template in Appendix). The incidence of all TEAEs, all TEAEs of CTCAE Grade 3 or higher, treatment-related TEAEs, treatment-related TEAEs of CTCAE Grade 3 or higher, serious TEAEs and serious treatment-related TEAEs will be summarized by System Organ Class (SOC) and Preferred Term (PT). Non treatment emergent serious AEs will be presented in a separate summary table. The summaries will include number of events, number of patients, and percentage of patients exposed reporting these events. Each of the analyses will be done for all TEAEs, for naxitamab treatment-related TEAEs and for GM-CSF treatment-related TEAEs.

The incidence of all TEAEs, treatment-related TEAEs, serious TEAEs and treatment-related serious TEAEs will also be summarized by CTCAE grade (see Template 6 in appendix). In addition, summary table for all TEAEs, treatment-related TEAEs, serious TEAEs and treatment-related serious TEAEs by grade will be generated.

TEAEs resulting in death, and TEAEs leading to trial drug discontinuation (1 for naxitamab and 1 for GM-CSF) will be summarized and listed.

TEAEs which occur after the first dose of GM-CSF and prior to the first dose of naxitamab will be summarized separately. SAEs with onset after signed informed consent and before start of GM-CSF treatment will be listed separately.

<u>Infusion related reactions (Definitions 1 and 2)</u>

Infusion reactions will be investigated in two different ways, based on the following 2 definitions:

(1) Infusion related reactions are naxitamab treatment-related TEAEs which occur on an infusion day (after the infusion start; if start time is missing they are included as infusion related reactions to be conservative);

(2) Infusion-related hypersensitivity TEAE is an AE that match a selected list of hypersensitivity terms based on reported preferred terms (including the MedDRA SMQ narrow scopes of anaphylactic reaction, angioedema, hypersensitivity, and severe cutaneous reaction), that occurs on a day of naxitamab infusion (after the infusion start) or the day after a naxitamab infusion irrespectively of relatedness to naxitamab.

Infusion reactions will be investigated for frequency of the event per naxitamab infusion, presented by SOC and PTs, side by side as 'all' and 'Grade 3 or higher' (cf. Table Template 1). Infusion reactions will also be summarized by severity/Grade (cf. Table Template 6).

Infusion-related hypersensitivity TEAEs (definition 2) will also be analyzed by use of steroid pre-medication. These data will also be presented by SOC and PTs by cycle and infusion as well as by cycle and severity/Grade incl number of patients with events (N), percentage of patients with events (%) and total number of reports of the events (E) (see Template 1 in appendix).

<u>Events of special interest (AESIs) and other selected conditions</u>

TEAEs of special interest (AESIs) as pre-defined in the protocol and judged by Investigator as AESIs will be analyzed separately in both a summary table and a listing The events include:

- PRES,
- Myelosuppression,
- Hypertension requiring antihypertension therapy or which persist over 90th percentile or higher requiring additional monitoring after naxitamab infusion.
- Adverse events leading to incomplete dosing during a cycle (meaning total planned dose was not given in a cycle)

Furthermore, TEAEs of infusion related reactions (definition 1 and 2), hypertension, pain (PTs that include the word PAIN, excluding the following two: Procedural pain and Vessel puncture site pain') and oedema (PTs that include the word OEDEMA) will be presented by treatment cycle and summarized by severity/CTCAE Grade including column/row with total (cf. Table Template 5a). These terms will also be summarized by infusion for all grades and for "Grade 3 or higher" events (cf. Table Template 5b).

Other selected conditions include the following: Hypersensitivity, peripheral neuropathy and neurological disorders of the eye, summaries for total and by CTCAE grade (see Template 6).

Hypersensitivity data include MedDRA SMQs narrow and broad searches. Data to be presented in a grouped narrow search and a grouped broad search for the following SMQs: SMQ

Anaphylactic reaction, SMQ Angioedema, SMQ Hypersensitivity, SMQ Severe cutaneous reaction.

Peripheral neuropathy data include MedDRA SMQs narrow and broad searches. Data to be presented in a grouped narrow search and a grouped broad search for the following SMQs; SMQ Peripheral neuropathy, SMQ Demyelination and SMQ Guillain-Barre Syndrome.

Neurological disorders of the eye data include MedDRA HLGT Neurological disorders of the eye.

When calculating the proportion of patients experiencing an AE, each patient will be counted only once for a given MedDRA SOC and PT as well as overall. For the frequency of the AE (e) all reports of the AE are counted. When AEs are summarized within levels of another AE assessment (e.g., intensity), the individual patient may count more than once.

A summary of the most frequent TEAEs (≥ 10%) and a summary of the most frequent treatment-related TEAEs (≥ 10%) will presented in tables by MedDRA SOC and PT in descending order of incidence within each SOC.

To relate pain to the use of opioids and ketamine, pain (PTs that include the word PAIN, excluding the following two: Procedural pain and Vessel puncture site pain') will be summarized by infusion and cycle in groups of use of opioids only, ketamine only, both or neither (cf. Table Template 9).

Summaries by SOC and PT of Grade 3 or higher events for all TEAEs, TEAEs related to naxitamab, serious TEAEs and serious TEAEs related to naxitamab will be presented for the following subgroups (see Table 1, Section 4.5 for specification): 1, 2, 3, 9, 10, 12. Displays will be similar to Template 1 while also including overall data, i.e. across all cycles.

Summaries by the SOCs vascular disorders and Cardiac disorders and associated PTs will be summarized by use of normal saline as pre-medication, overall, by Grade and by Grade 3+.

8.2 Clinical laboratory evaluations

The following clinical laboratory parameters will be summarized and plotted: Sodium. Potassium, AST, ALT, ALP, Albumin, Bilirubin, LDH, BUN, Creatinine, White blood cell count, Red blood cell count, Hemoglobin, Hematocrit, Mean corpuscular hemoglobin, Mean corpuscular hemoglobin concentration, Mean corpuscular volume, Platelets, White Blood Cell (WBC), Neutrophils (absolute), Lymphocytes (absolute), Monocytes (absolute), Eosinophils (absolute) and Basophils (absolute).

Laboratory values with absolute and percentage change from baseline will be summarized by cycle and infusion day, as well as from baseline to end of treatment (cf. Table Template 7). Computational note: if a baseline value=0 and the post-baseline=0 then the relative change from

baseline=0%. Laboratory values will also be listed, with flags for abnormal values and if marked as clinically significant by the investigator.

For a variable, baseline is defined as the last non-missing value available prior to first naxitamab infusion. If only one value exists at the day of first naxitamab administration and information on time of infusion or laboratory sample is missing, the corresponding laboratory sample is assumed to have been take before the administration. In case more laboratory samples exist on first day of infusion (likely because the patient experienced adverse events) the first of these is used as baseline.

Laboratory values will be displayed in SI units.

In addition to the above summary tables, there will be further assessments of the laboratory parameters:

- Scatter plots of post-baseline vs. baseline laboratory values including a diagonal line of no change (i.e. shift-plots) will be presented in panels of infusion days within cycles and by cycle, see Figure Template 1 in Appendix 1.
- For each lab-parameter with post-baseline assessments, the following plots will be produced:
  - trend-plots (e.g. mean value with 95% confidence intervals) over time (per cycle and assessment day within cycle, as per the trial assessment schedule), see Figure Template 2 in Appendix 1.
  - individual patient profile- (a.k.a. spaghetti-) plots for all patients, see Figure Template 3 in Appendix 1.

In these individual patient profile plots reference ranges and NCI CTCAE Version 5.0 (reference:https://ctep.cancer.gov/protocoldevelopment/electronic_applications/docs/CTCAE_v5_Quick_Reference_8.5x11.pdf) grading thresholds will be presented, as available.

These plots will include all pre-treatment measurements made up to 30 days before the first dose (the screening period).

Hy's law plot will be provided (See Figure Template 4 in Appendix 1):

- Peak bilirubin vs. peak ALT (eDISH1)

- For subjects in upper right quadrant of the eDISH1 plot an eDISH2 plot will be done

Individual parameters to include in eDISH2: ALT, AST, ALP, total bilirubin, serum albumin, leukocytes and eosinophils.

8.3 Vital signs

Vital signs will include measurements of heart rate, respiratory rate, peripheral oxygen saturation, temperature, and blood pressure. These will be measured and recorded repeatedly in connection with each treatment cycle and in connection with adverse events. Temperature must be measured by using the same method (e.g., an ear thermometer). These measurements will be listed and summarized by cycle and time point or visit.

Heart rate, blood pressure and peripheral oxygen saturation will be presented in shift-plots, cf. Figure Template 1 in Appendix 1; from baseline to each of the following three:

- End-of-Treatment assessment
- Highest post-baseline assessment
- Lowest post-baseline assessment.

The same parameters will also be presented in as well as scatter plots of values vs. time from infusion, cf. Figure Template 5 in Appendix 1.

Systolic and diastolic blood pressure will be presented in individual patient profile plots, see Figure Template 6 in Appendix 1.

A physical examination should be performed at screening, prior to each treatment cycle, and at EOT. Significant findings are to be recorded as medical history or adverse events and thus separate summaries of physical exam results will not be performed.

8.4 Electrocardiogram

ECGs will be performed at screening, before cycles 2 and 4, and at EOT. ECG results will be recorded as normal, abnormal, not clinically significant, and abnormal, clinically significant. These results will be listed and summarized by the frequencies and percentages of patients with a treatment-emergent abnormal clinically significant result by cycle and at EOT. Patients who develop abnormal clinically significant ECG will be listed including ECG description reported in the eCRF.

8.5 Pain assessments

Pain scores are assessed using the FLACC scale for patients ≤5 years and Wong-Baker FACES® pain scale for patients >5 years. These will be recorded prior to each infusion of naxitamab, worst pain during 30-min infusion, pain approximately 15 minutes after end of infusion, and prior to discharge. Pain scores be listed and summarized with descriptive statistics by cohort, cycle, infusion, and time point.

Pain medication use on days of naxitamab infusion will be summarized by i) Opioids only, ii) Ketamine only, and iii) Opioids and ketamine.

8.6 Performance assessments

The Lansky (children < 16 years) or Karnofsky (children ≥ 16 years) performance score will be analyzed to evaluate gross neurologic function and a measure of a patient's overall function. Performance testing will be done at baseline, prior to cycles 4, 7 and 10, and at EOT. Changes from baseline will be summarized by cycle and at EOT and the data will be listed.

Quality of life assessments (happiness and activity level)

Happiness and activity will be assessed by use of a visual analogue scale (VAS) and be analyzed as a proxy for quality of life. These assessments will be done each evening from Cycle 1 day -4 to cycle 2 day 7. Data will be listed and summarized with descriptive statistics by cohort, overall and by day.

8.7 Hospitalization days

The number of hospitalization days, duration of hospitalization as well as the number of patients hospitalized overall and during each cycle will be summarized by reason and in total.

9 Changes to planned analyses

The objectives and endpoints in this SAP have been aligned with the availability and maturity of the data at the time of the preplanned interim analysis.

The addition of descriptive tables for infusion-related AE according to FDA criteria, and addition of other efficacy endpoints for CR, confirmed response, PFS and OS are not included in the scope of the preplanned interim analyses defined in section 12.3 in the protocol and have been added to cover other purposes.

10 SAP version history

Table 2 Change log

**Version and date**	**Revision author**	**Comments**
v1.0, 08MAR2020	[redacted]	Original SAP version for trial 201 reporting.
Interim v1.0, 12FEB2021	[redacted]	SAP for ad hoc interim analysis based on the data cut-off at 05AUG2020
Interim v2.0, 22FEB2021	[redacted]	Revised SAP for ad hoc interim analysis based on the data cut-off at 05AUG2020<br><br>Section 7.1: i) Summary of TEAEs leading to trial withdrawal is omitted, reflecting an information collection redesign in protocol version 10 and eCRFs Revision 8; ii) Omitted worst-case imputation of missing AE intensity: an imputation would have lead to having a grade 5 event in a case where this was not the fact; iii) Clarified that the summaries of treatment-related AEs by subgroups should be summaries of AEs related to naxitamab.<br><br>Section 7.4: Additional details for the presentations of shift-plots of vital signs are provided.<br><br>Table Template 5b: Defined *r* in the table template.<br><br>Table Template 7: Clarified that summaries should be presented by cycle and infusion days.<br><br>All sections: Minor typographic edits.
Interim v3.0, 22FEB2021	[redacted]	Revised SAP for ad hoc interim analysis based on the data cut-off at 05AUG2020.<br><br>Section 6.3: Added subgroup analysis: systemic steroids as pre-medication.

Interim v4.0, 31JAN2022	[redacted]	SAP updated to reflect preplanned interim analysis as described in protocol v12.0, based on the data cut-off at 31DEC2021.<br><br>The revision also entails additional descriptive baseline tables for use of steroids as pre-medication, descriptive tables for infusion-related AE according to FDA criteria, and addition of endpoints outside the formal scope of the preplanned interim analysis, such as CR, ORR for confirmed response and long-term follow-up endpoints PFS and OS.<br><br>This SAP was prepared using the Y-mAbs template TPL-00072 v2.0.
Interim v5.0, 30MAR2022	[redacted]	Explicit dates for data cut-off for the Full Analysis Set and Safety Analysis Set regarding the preplanned interim analysis was added.

Appendix 1 Templates for Tables and figures

This appendix contains outlines of table and figure layouts. The actual result may differ.

Table Template 1

Infusion related reactions by infusion - Cycle x

SOC	Preferred Term	Infusion 1<br>All AEs<br>n/N (%) E<br>/<br>Grade 3+<br>n/N (%) E	Infusion 2<br>All AEs<br>n/N (%) E<br>/<br>Grade 3+<br>n/N (%) E	Infusion 3<br>All AEs<br>n/N (%) E<br>/<br>Grade 3+<br>n/N (%) E	Total<br>All AEs<br>n/N (%) E<br>/<br>Grade 3+<br>n/N (%) E

Table Template 2

Adverse events category	Cohort 1<br>(N=24)<br>[n(%)], #events	Cohort 2<br>(N=1)<br>[n(%)], #events	Overall<br>(N=25)<br>[n(%)], #events
Treatment-emergent adverse events	x (yy.y) zz	x (yy.y) zz	x (yy.y) zz
Related treatment-emergent adverse events	x (yy.y) zz	x (yy.y) zz	x (yy.y) zz
Naxitamab	x (yy.y) zz	x (yy.y) zz	x (yy.y) zz
GM-CSF	x (yy.y) zz	x (yy.y) zz	x (yy.y) zz
Treatment-emergent grade 3 or higher events	x (yy.y) zz	x (yy.y) zz	x (yy.y) zz
Related treatment-emergent grade 3 or higher events	x (yy.y) zz	x (yy.y) zz	x (yy.y) zz
Naxitamab	x (yy.y) zz	x (yy.y) zz	x (yy.y) zz
GM-CSF	x (yy.y) zz	x (yy.y) zz	x (yy.y) zz
Serious treatment-emergent adverse events	x (yy.y) zz	x (yy.y) zz	x (yy.y) zz
Serious treatment-emergent grade 3 or higher	x (yy.y) zz	x (yy.y) zz	x (yy.y) zz
Related serious treatment-emergent adverse events	x (yy.y) zz	x (yy.y) zz	x (yy.y) zz
Naxitamab	x (yy.y) zz	x (yy.y) zz	x (yy.y) zz
GM-CSF	x (yy.y) zz	x (yy.y) zz	x (yy.y) zz
Related serious treatment-emergent grade 3 or higher	x (yy.y) zz	x (yy.y) zz	x (yy.y) zz
Naxitamab	x (yy.y) zz	x (yy.y) zz	x (yy.y) zz
GM-CSF	x (yy.y) zz	x (yy.y) zz	x (yy.y) zz
Treatment-emergent adverse events leading to discontinuation from treatment	x (yy.y) zz	x (yy.y) zz	x (yy.y) zz
Treatment-emergent adverse events with outcome of death	x (yy.y) zz	x (yy.y) zz	x (yy.y) zz

Table Template 3

CONFIDENTIAL FINAL Y-mAbs Therapeutics
Protocol 201 (Interim) Naxitamab

Table 14.3.1.2.2
Summary of Treatment Emergent Adverse Events
CTCAE Grade 3 or Higher
(Safety Analysis Set)

System Organ Class/ Preferred Term	Cohort 1 (N=24) [n(%)], #events	Cohort 2 (N=1) [n(%)], #events	All Subjects (N=25) [n(%)], #events
Subjects with at least one grade 3 or higher TEAE	22 (91.7), 385	1 (100), 4	23 (92.0), 389
General disorders and administration site conditions	18 (75.0), 211	0	18 (72.0), 211
Pain	18 (75.0), 208	0	18 (72.0), 208
Chest pain	1 (4.2), 3	0	1 (4.0), 3

Table Template 4 (NB the notes below the table)

CONFIDENTIAL FINAL Y-mAbs Therapeutics
Protocol 201 (Interim) Naxitamab

Table 14.3.1.3.3.1
Summary of Related Treatment Emergent Adverse Events
by CTCAE Grade
(Safety Analysis Set)

System Organ Class/ Preferred Term	Severity (CTCAE Grade)	Cohort 1 (N=24) [n(%)]	Cohort 2 (N=1) [n(%)]	All Subjects (N=25) [n(%)]
Subjects with at least one related TEAE		24 (100)	1 (100)	25 (100)
General disorders and administration site conditions	1: Mild	0	0	0
	2: Moderate	6 (25.0)	1 (100)	7 (28.0)
	3: Severe	18 (75.0)	0	18 (72.0)
	4: Life-threatening	0	0	0
Pain	1: Mild	0	0	0
	2: Moderate	6 (25.0)	1 (100)	7 (28.0)
	3: Severe	18 (75.0)	0	18 (72.0)
	4: Life-threatening	0	0	0
Pyrexia	1: Mild	11 (45.8)	0	11 (44.0)
	2: Moderate	4 (16.7)	1 (100)	5 (20.0)
	3: Severe	0	0	0
	4: Life-threatening	0	0	0

The template above should also include the following modifications:

- remove the explanations (mild, moderate etc.) of the severities
- after grade 4, add a row with grade 5
- below grade 5, add a row “All grades” that provides a summary across all grades

A patient may be included in several rows (grades), i.e. this table should not present the maximum severity only.

Table Template 5a

Preferred Term	Cycle	Sub-jects	Grade 1 [n(%), e]	Grade 2 [n(%), e]	Grade 3 [n(%), e]	Grade 4 [n(%), e]	Grade 3+ [n(%), e]	Any Grade [n(%), e]
Infusion-related reaction[1]	1	25	22 (88.0), 86	25 (100.0), 96	17 (68.0), 95	1 (4.0), 1	17 (68.0), 96	25 (100.0),278
	2	23	20 (87.0), 49	19 (82.6), 96	16 (69.6), 75	1 (4.3), 1	16 (69.6), 76	23 (100.0),221
	3	18	12 (66.7), 30	18 (100.0), 65	13 (72.2), 56	1 (5.6), 1	14 (77.8), 57	18 (100.0),152
	4	13	12 (92.3), 35	12 (92.3), 55	10 (76.9), 42	0	10 (76.9), 42	13 (100.0),132
	5	12	9 (75.0), 15	10 (83.3), 53	8 (66.7), 35	0	8 (66.7), 35	12 (100.0),103
	6	11	11 (100.0), 24	9 (81.8), 55	7 (63.6), 30	0	7 (63.6), 30	11 (100.0),109
	7	11	8 (72.7), 15	11 (100.0), 52	7 (63.6), 25	0	7 (63.6), 25	11 (100.0), 92
	8	2	1 (50.0), 2	2 (100.0), 4	2 (100.0), 5	0	2 (100.0), 5	2 (100.0), 11
	Any	25	25 (100.0),256	25 (100.0),476	20 (80.0),363	3 (12.0), 3	21 (84.0),366	25 (100.0), 98
Pain[2]	1	25	14 (56.0), 21	16 (64.0), 34	17 (68.0), 46	0	17 (68.0), 46	25 (100.0),101
	2	23	7 (30.4), 13	10 (43.5), 23	16 (69.6), 48	0	16 (69.6), 48	23 (100.0), 84
	3	18	3 (16.7), 3	11 (61.1), 20	12 (66.7), 33	0	12 (66.7), 33	18 (100.0), 56
	4	13	4 (30.8), 4	6 (46.2), 14	9 (69.2), 27	0	9 (69.2), 27	13 (100.0), 45
	5	12	3 (25.0), 3	4 (33.3), 12	8 (66.7), 23	0	8 (66.7), 23	12 (100.0), 38
	6	11	3 (27.3), 4	6 (54.5), 14	7 (63.6), 22	0	7 (63.6), 22	11 (100.0), 40
	7	11	3 (27.3), 3	4 (36.4), 10	7 (63.6), 20	0	7 (63.6), 20	11 (100.0), 33
	8	2	0	0	2 (100.0), 5	0	2 (100.0), 5	2 (100.0), 5
	Any	25	17 (68.0), 51	17 (68.0),127	18 (72.0),224	0	18 (72.0),224	25 (100.0),402
Bronchospasm	1	25	0	11 (44.0), 21	4 (16.0), 4	0	4 (16.0), 4	15 (60.0), 25
	2	23	0	11 (47.8), 21	1 (4.3), 1	0	1 (4.3), 1	12 (52.2), 22
	3	18	0	7 (38.9), 8	2 (11.1), 3	0	2 (11.1), 3	9 (50.0), 11
	4	13	0	6 (46.2), 9	1 (7.7), 1	0	1 (7.7), 1	7 (53.8), 10
	5	12	0	4 (33.3), 8	0	0	0	4 (33.3), 8
	6	11	1 (9.1), 1	4 (36.4), 7	0	0	0	5 (45.5), 8
	7	11	0	5 (45.5), 7	0	0	0	5 (45.5), 7
	8	2	0	0	0	0	0	0
	Any	25	1 (4.0), 1	16 (64.0), 81	7 (28.0), 9	0	7 (28.0), 9	19 (76.0), 91
Hypertension	1	25	5 (20.0), 6	3 (12.0), 3	1 (4.0), 1	0	1 (4.0), 1	8 (32.0), 10
	2	23	2 (8.7), 3	1 (4.3), 2	0	0	0	2 (8.7), 5
	3	18	1 (5.6), 1	1 (5.6), 1	0	0	0	2 (11.1), 2
	4	13	2 (15.4), 2	1 (7.7), 1	0	0	0	2 (15.4), 3
	5	12	2 (16.7), 2	1 (8.3), 1	0	0	0	3 (25.0), 3
	6	11	1 (9.1), 1	1 (9.1), 1	0	0	0	1 (9.1), 2
	7	11	0	0	0	0	0	0
	8	2	0	0	0	0	0	0
	Any	25	9 (36.0), 15	3 (12.0), 9	1 (4.0), 1	0	1 (4.0), 1	11 (44.0), 25

Table Template 5b

		Grade 3 or Higher			All Grades		
Preferred Term	Cycle	Infusion 1 [n/r(%), e]	Infusion 2 [n/r(%), e]	Infusion 3 [n/r(%), e]	Infusion 1 [n/r(%), e]	Infusion 2 [n/r(%), e]	Infusion 3 [n/r(%), e]
Infusion-related reaction[1]	1	17/25 (68.0), 50	16/24 (66.7), 25	14/23 (60.9), 21	25/25 (100.0),128	24/24 (100.0), 85	21/23 (91.3), 65
	2	16/23 (69.6), 30	15/22 (68.2), 23	15/22 (68.2), 23	23/23 (100.0), 94	21/22 (95.5), 67	22/22 (100.0), 60
	3	13/18 (72.2), 26	12/17 (70.6), 17	11/16 (68.8), 14	18/18 (100.0), 69	16/17 (94.1), 50	15/16 (93.8), 33
	4	9/13 (69.2), 15	10/13 (76.9), 15	9/13 (69.2), 12	13/13 (100.0), 53	13/13 (100.0), 41	13/13 (100.0), 38
	5	8/12 (66.7), 16	8/12 (66.7), 12	7/12 (58.3), 7	12/12 (100.0), 39	12/12 (100.0), 34	12/12 (100.0), 30
	6	7/11 (63.6), 11	7/11 (63.6), 10	7/11 (63.6), 9	11/11 (100.0), 40	11/11 (100.0), 38	11/11 (100.0), 31
	7	7/11 (63.6), 11	6/11 (54.5), 7	7/10 (70.0), 7	10/11 (90.9), 35	11/11 (100.0), 30	10/10 (100.0), 27
	8	2/2 (100.0), 2	1/2 (50.0), 1	2/2 (100.0), 2	2/2 (100.0), 5	1/2 (50.0), 2	2/2 (100.0), 4
	Any	19/25 (76.0),161	18/24 (75.0),110	17/23 (73.9), 95	25/25 (100.0),463	24/24 (100.0),347	23/23 (100.0),288
Pain[2]	1	17/25 (68.0), 17	16/24 (66.7), 16	13/23 (56.5), 13	25/25 (100.0), 44	24/24 (100.0), 28	19/23 (82.6), 29
	2	15/23 (65.2), 16	15/22 (68.2), 16	15/22 (68.2), 16	23/23 (100.0), 31	21/22 (95.5), 29	22/22 (100.0), 24
	3	11/18 (61.1), 11	10/17 (58.8), 11	11/16 (68.8), 11	18/18 (100.0), 21	16/17 (94.1), 18	14/16 (87.5), 17
	4	9/13 (69.2), 9	9/13 (69.2), 9	9/13 (69.2), 9	12/13 (92.3), 15	13/13 (100.0), 14	13/13 (100.0), 16
	5	8/12 (66.7), 8	8/12 (66.7), 8	7/12 (58.3), 7	12/12 (100.0), 13	12/12 (100.0), 13	12/12 (100.0), 12
	6	7/11 (63.6), 8	7/11 (63.6), 7	7/11 (63.6), 7	11/11 (100.0), 15	11/11 (100.0), 12	11/11 (100.0), 13
	7	7/11 (63.6), 7	6/11 (54.5), 6	7/10 (70.0), 7	11/11 (100.0), 12	10/11 (90.9), 11	10/10 (100.0), 10
	8	2/2 (100.0), 2	1/2 (50.0), 1	2/2 (100.0), 2	2/2 (100.0), 2	1/2 (50.0), 1	2/2 (100.0), 2
	Any	17/25 (68.0), 78	16/24 (66.7), 74	17/23 (73.9), 72	25/25 (100.0),153	24/24 (100.0),126	23/23 (100.0),123
Bronchospasm	1	4/25 (16.0), 4	0/24	0/23	11/25 (44.0), 11	7/24 (29.2), 7	7/23 (30.4), 7
	2	1/23 (4.3), 1	0/22	0/22	9/23 (39.1), 9	7/22 (31.8), 7	6/22 (27.3), 6
	3	2/18 (11.1), 2	1/17 (5.9), 1	0/16	7/18 (38.9), 7	2/17 (11.8), 2	2/16 (12.5), 2
	4	0/13	1/13 (7.7), 1	0/13	4/13 (30.8), 4	3/13 (23.1), 3	3/13 (23.1), 3
	5	0/12	0/12	0/12	3/12 (25.0), 3	2/12 (16.7), 2	3/12 (25.0), 3
	6	0/11	0/11	0/11	4/11 (36.4), 4	3/11 (27.3), 3	1/11 (9.1), 1
	7	0/11	0/11	0/10	2/11 (18.2), 2	3/11 (27.3), 3	2/10 (20.0), 2
	8	0/2	0/2	0/2	0/2	0/2	0/2
	Any	6/25 (24.0), 7	2/24 (8.3), 2	0/23	18/25 (72.0), 40	11/24 (45.8), 27	12/23 (52.2), 24

r-denotes the number of patients who received the relevant infusion in the cycle; the denominator for the %.

[1] Definition 1 of infusion reaction.

[2] Pain is defined as the collection the following terms: PTs of pain, abdominal pain, pain in extremity, bone pain, neck pain, back pain, non-cardiac chest pain, flank pain, and musculoskeletal pain.

Table Template 6

Table 2.6: Treatment Emergent Serious Adverse Events by grade, SOC and preferred term – Safety analysis set

	Grade 1<br>N (%) E	Grade 2<br>N (%) E	Grade 3<br>N (%) E	Grade 4<br>N (%) E	Grade 5<br>N (%) E	Total<br>N (%) E
**Any Serious Adverse Event**	2 (3.7) 3	5 (9.3) 6	13 (24.1) 14	9 (16.7) 9		23 (42.6) 32
**General disorders and administration site conditions**	2 (3.7) 2	1 (1.9) 1	3 (5.6) 3			6 (11.1) 6
Pyrexia	2 (3.7) 2	1 (1.9) 1				3 (5.6) 3
Pain			2 (3.7) 2			2 (3.7) 2
Fatigue			1 (1.9) 1			1 (1.9) 1

A patient may be included in several rows (grades), *i.e.* this table should not present the maximum severity only.

Table Template 7

In this table, summarize for each cycle and infusion day:

- Results (unit)
- Absolute change from baseline (same unit as results)
- Relative change from baseline (%)

Visits			Cohort 1<br>(N=24)	Cohort 2<br>(N=1)	Overall<br>(N=25)
Test (units): Alanine Aminotransferase (U/L)					
Cycle 2	Change from baseline	n	68	2	70
		Mean	-8.6	-1.5	-8.4
		Median	-5.0	-1.5	-5.0
		SD	17.71	4.95	17.50
		Q1, Q3	-12, -1	-5, 2	-11, -1
		Min, Max	-69, 49	-5, 2	-69, 49
Cycle 3	Results	n	49	3	52
		Mean	22.5	20.0	22.3
		Median	15.0	17.0	15.5
		SD	28.08	9.85	27.31
		Q1, Q3	12, 19	12, 31	12, 20
		Min, Max	6, 170	12, 31	6, 170
	Change from baseline	n	49	3	52
		Mean	0.4	0.0	0.4
		Median	-4.0	-3.0	-3.5
		SD	27.26	9.85	26.52
		Q1, Q3	-10, 0	-8, 11	-9, 0
		Min, Max	-22, 155	-8, 11	-22, 155

Table Template 8

Cycle	Day			per i.v.	per oral	transdermal	Total
1	1*	N (%)					
		Doses per patient	mean				
			median				
			SD				
			range				
		Total Dose (mg)	mean				
			median				
			SD				
			range				
		Total Dose (mg/kg)	mean				
			median				
			SD				
			range				
	2	N (%)					
		Doses per patient	mean				
			median				
			SD				
			range				
…	…						

For Days 1*, 2, 3*, 4, 5*, 6 and All (All=from infusion day 1 to day before first infusion in subsequent cycle) in each treatment cycle. Cf. Table Template 9 below.
* = naxitamab infusion day
N = number of patients who received. % = based on pts still in trial.
Doses per patient= the number of opioid administrations per patient
Total doses converted to morphine i.v. equivalent

Table Template 9

Cycle	Day	AE**	NCI CTC AE Grade 1	2	3	4	All
1	1*	Pain	N (%) E	N (%) E	N (%) E	N (%) E	N (%) E
	2	Pain	N (%) E	N (%) E	N (%) E	N (%) E	N (%) E
	3*	Pain	N (%) E	N (%) E	N (%) E	N (%) E	N (%) E
	4	Pain	N (%) E	N (%) E	N (%) E	N (%) E	N (%) E
	5*	Pain	N (%) E	N (%) E	N (%) E	N (%) E	N (%) E
	6	Pain	N (%) E	N (%) E	N (%) E	N (%) E	N (%) E
	All	Pain	N (%) E	N (%) E	N (%) E	N (%) E	N (%) E
2	1*	Pain	N (%) E	N (%) E	N (%) E	N (%) E	N (%) E
	2	Pain	N (%) E	N (%) E	N (%) E	N (%) E	N (%) E
	3*	Pain	N (%) E	N (%) E	N (%) E	N (%) E	N (%) E
	4	Pain	N (%) E	N (%) E	N (%) E	N (%) E	N (%) E
	5*	Pain	N (%) E	N (%) E	N (%) E	N (%) E	N (%) E
	6	Pain	N (%) E	N (%) E	N (%) E	N (%) E	N (%) E
	All	Pain	N (%) E	N (%) E	N (%) E	N (%) E	N (%) E

* = naxitamab infusion day

** AE Pain is here defined as the collection the following terms: PTs of pain, abdominal pain, pain in extremity, bone pain, neck pain, back pain, non-cardiac chest pain, flank pain, and musculoskeletal pain.

This should be stated in a footnote to the table.

All=from infusion day 1 to day before first infusion in subsequent cycle in each treatment cycle.

Figure Template 1

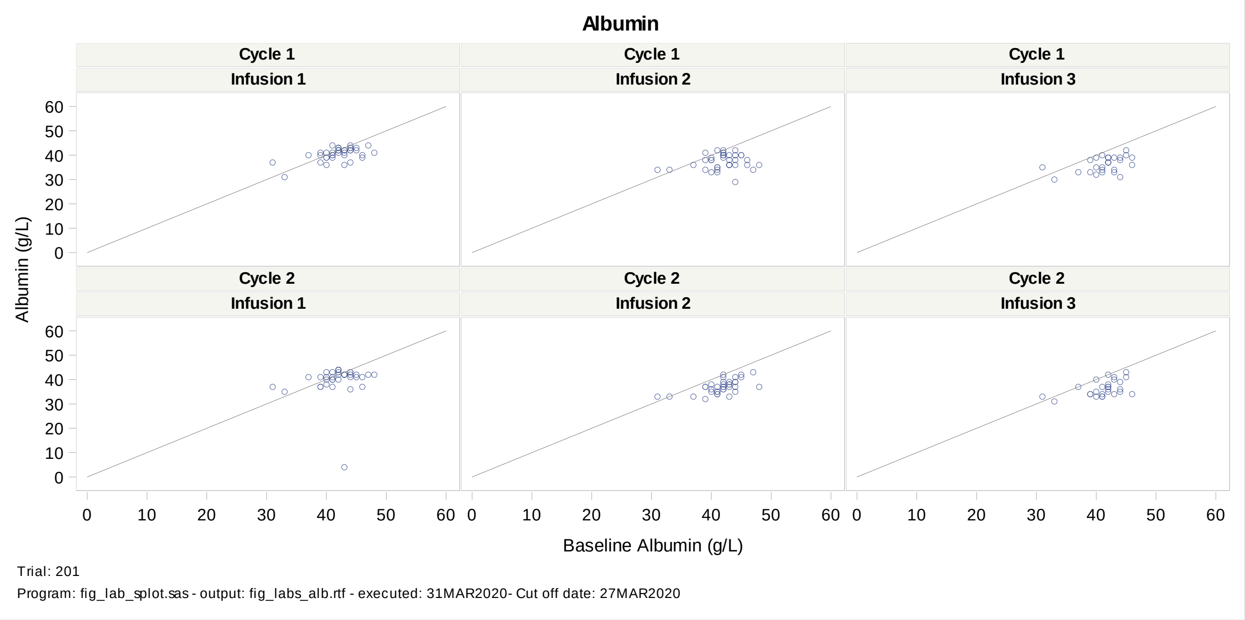

Figure Template 2

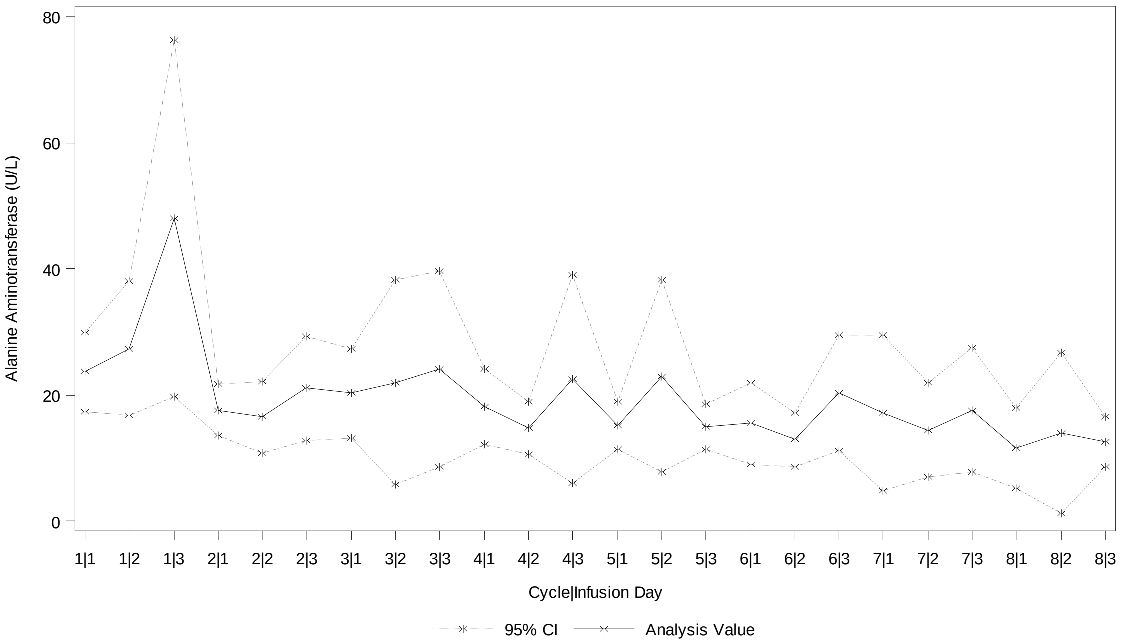

Recommendation: replace the x|y in the horizontal axis tick mark labels with CyxInfy (short for Cycle x Infusion y).

Figure Template 3

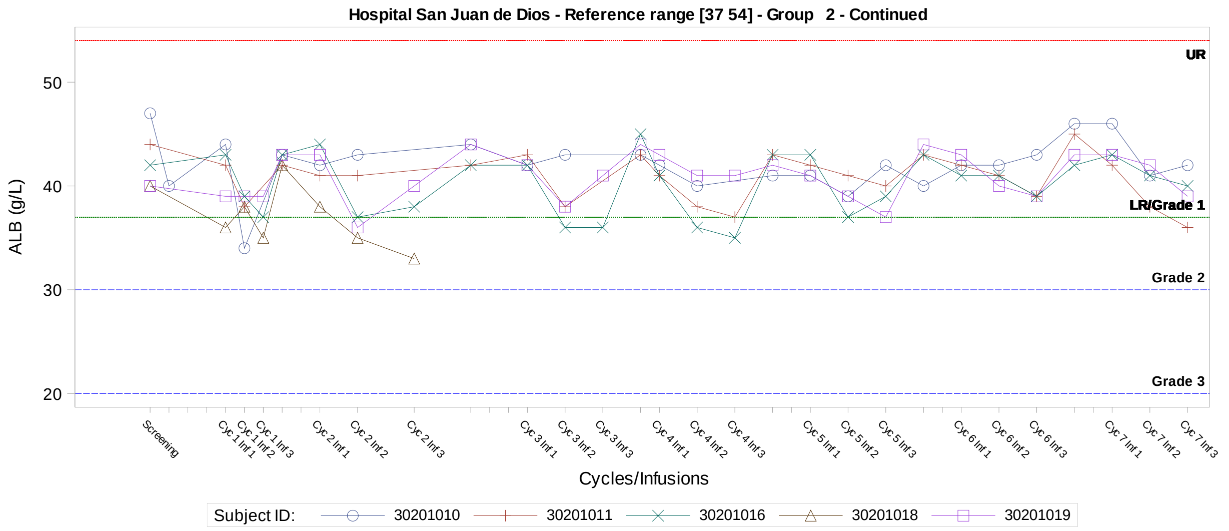

CTCAE Term: Hypoalbuminemia

Trial: 201

Program: fig_lab.sas - output: fig_lab_alb.rtf - executed: 14APR2020- Cut off date: 14APR2020

Include only 1 patient per plot to allow for timing and individual reference ranges.

Figure Template 4

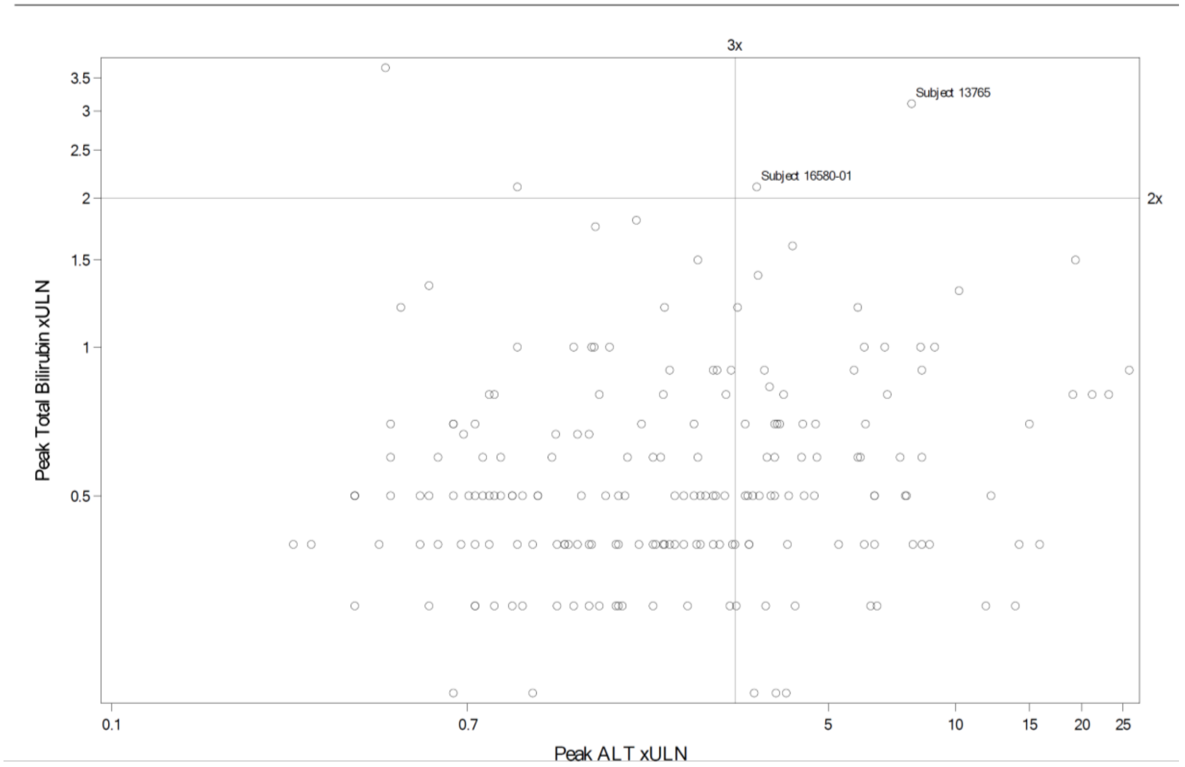

xx

Figure Template 5

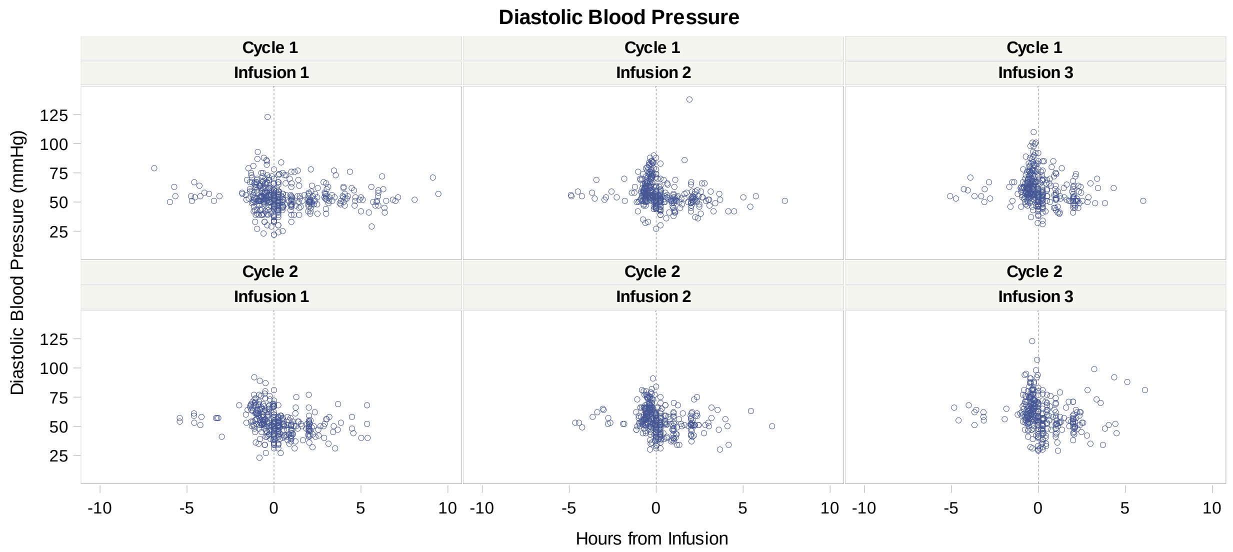

Trial: 201

Program: fig_vs.sas - output: fig_dia.rtf - executed: 31MAR2020- Cut off date: 27MAR2020

Figure Template 6

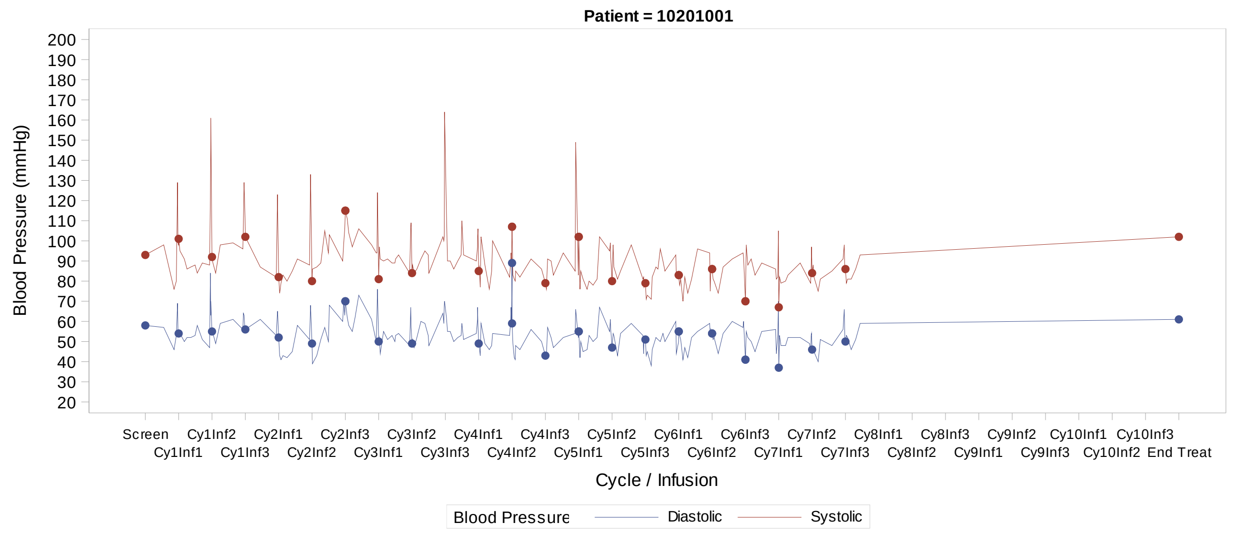

Filled dots mark Screening, Infusion End Time and End of Treatment.

Trial: 201

Program: fig_bp.sas - output: fig_bp.rtf - executed: 31MAR2020- Cut off date: 27MAR2020

Figure Template 7

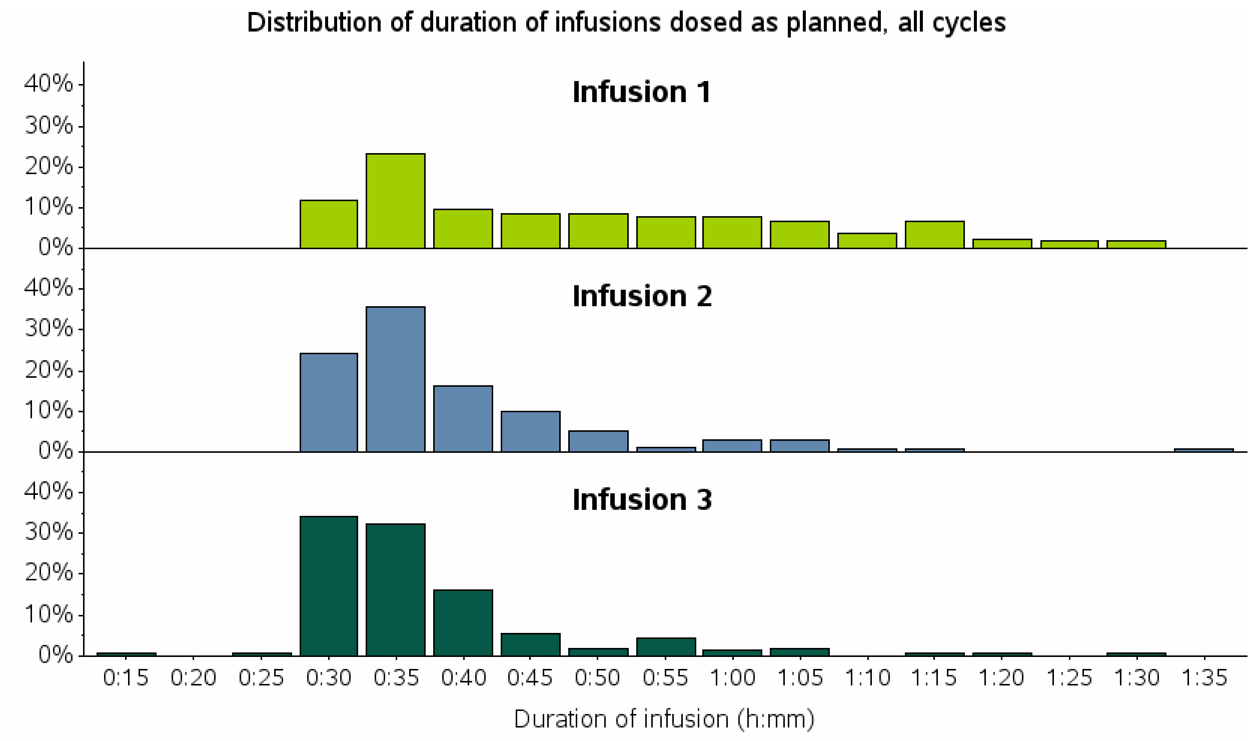

Figure Template 8

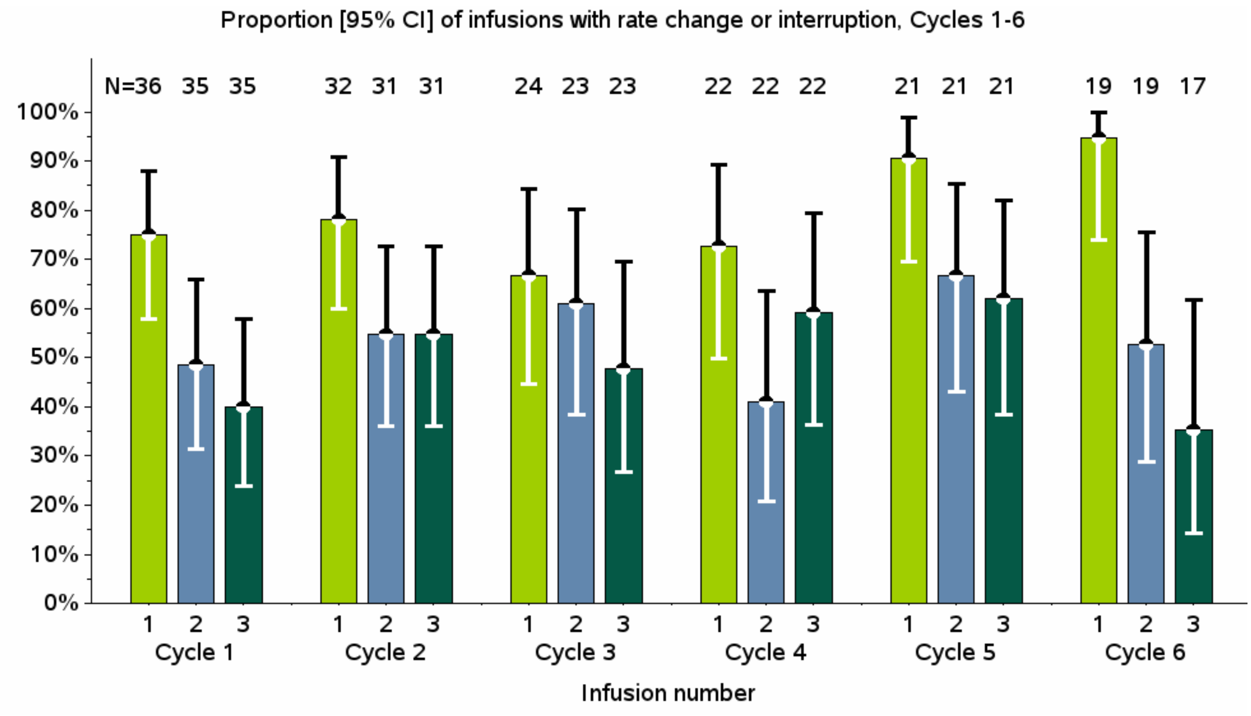

Major changes to the Trial 201 protocol

Amendment Number	Protocol version (Effective date)	Major Changes
1.0	5.0[a]<br>(12-Apr-2018)	1. Trial phase changed from Phase 3 to Phase 2<br>2. Exclusion criterion reworded; changed from *'chemotherapy or immunotherapy within 3 weeks prior to the start of naxitamab'* to *'any systemic anti-cancer therapy (incl chemot*herapy or immunotherapy) within 3 weeks prior to the start of GM-CSF'<br>3. Shortening of the long-term FU from 5 years to 3 years<br>4. Inclusion criterion clarified—specifically, that primary and secondary refractory osteomedullary disease included disease in bone, BM or both<br>5. Updated the inclusion criteria for acceptable hematological status<br>6. Withdrawal criterion for anaphylaxis updated and withdrawal criterion added for posterior reversible encephalopathy syndrome [PRES]<br>7. Criteria for response assessment were updated to follow revised INRC as per Park et al 2017<br>8. Added two adverse events of special interest (AESIs) PRES and myelosuppression Grade 4, respectively
2.0	6.0<br>(30 Aug 2018)	1. To ensure consistent definition of primary refractory disease and incomplete response to salvage therapy, requirements added for prior chemotherapy<br>2. Added the secondary objective 'to evaluate the complete response to naxitamab and GM-CSF'<br>3. Added the secondary objective 'to evaluate the safety of naxitamab + GM-CSF in subjects with positive ADA at trial entry'<br>4. Clarified secondary endpoint on DoR; changed from 'from patient response to PD' to 'first objective response (CR or PR) to PD; and added censoring rule for initiation of new anti-cancer therapy<br>5. Added secondary endpoint "Complete response rate, during the naxitamab treatment period, centrally assessed according to the INRC"<br>6. Censoring rule clarified for the secondary endpoint on overall survival (OS), 'last date known to be alive' added<br>7. Added secondary endpoint 'IV opioid use for each cycle during the trial defined as total dosage of IV morphine (or equivalent opioid) administered 2 hours before infusion until 4 hours after end of infusion of naxitamab'<br>8. Added secondary endpoint 'In subjects with positive ADA at trial inclusion, safety will be evaluated by the incidence of AEs and SAEs graded according to CTCAE, version 4.0'<br>9. Added the exclusion criterion, 'prior treatment with naxitamab'<br>10. Added the exclusion criterion, 'Karnofsky/Lansky score <50%'

Amendment Number	Protocol version (Effective date)	Major Changes
		11. Added withdrawal criterion on atypical hemolytic uremic syndrome (HUS)<br>12. Definition of Cohort 1 and 2 introduced: Cohort 1: Patients screened negative for HAHA, with disease not confined to BM, and with no pre-planned radiotherapy; Cohort 2: Patients with pre-planned radiotherapy, or screened positive for HAHA, or with BM affection only<br>13. Updated schedule of BM aspirates and biopsies to include 1 additional response assessment
3.0 and 4.0	7.0<br>(18 Oct 2018)	1. Added exclusion criteria, 'history of anaphylactic reactions CTCAE Grade 4 related to prior GD2 antibody therapy'<br>2. Updated naxitamab vial concentration from 2 to 4 mg/mL
5.0	8.0<br>(07 Feb 2019)	1. Added the secondary endpoint, 'number and percentage of infusions done in an outpatient setting'<br>2. Clarified exclusion criteria for evaluable NB outside of bone and BM—i.e., criteria for MIBG or FDG uptake to establish soft tissue disease<br>3. Corticosteroid introduced as naxitamab premedication
6.0	9.0<br>(20 Sep 2019)	1. Recruitment period increased from 24 to 60 months<br>2. Increase in sample size from 37 to 85 subjects in Cohort 1 to accommodate regulatory request for more data on Progression Free Survival (PFS)<br>3. Cohort 1 definition clarified: subjects who are ADA-negative *at baseline* and with no pre-planned radiotherapy *for metastatic lesions*; As per regulatory request: Subjects with disease in BM only included in Cohort 1<br>4. Addition of exclusion criteria:<br>a. Subjects with prior CNS NB disease within 6 months of the first GM-CSF dose<br>b. Prior treatment with omburtamab (mu8H9) within 6 months of the first dose of GM-CSF<br>c. Subjects who had prior allo-SCT<br>5. AESI added 'hypertension that requires antihypertension therapy or persists at the 90th percentile or higher of BP for age, height, and sex'
7.0	10.0<br>(27 Mar 2020)	1. Updated inclusion criterion specifying that if disease is present only in the BM, the involvement must be >5% as per Park et al, 2017<br>2. The following exclusion criteria were added:<br>a. Subjects who received Hematopoietic Progenitor Cell (HPC) boost or "top-up" of allogenic stem cells (lymphocyte-depleted) within 2 months of the first dose GM-CSF<br>b. Any clinically meaningful abnormal finding in physical examination, vital signs, ECG, hematology, clinical chemistry, or urinalysis prior to inclusion into the trial, which in the opinion of the

Amendment Number	Protocol version (Effective date)	Major Changes
		investigator, may put the subject at risk because of his/her participation in the study
8.0	11.0<br>(03-Dec-2020)	1. QoL added as secondary objective and happiness and activity levels measured over time added as secondary endpoint<br>2. The frequency of long-term FU visits was changed from biyearly to quarterly<br>3. As per regulatory request to provide more data on PFS, response assessments during 1st year after end of treatment (EOT) were changed from twice yearly with the option to be performed at referral site to response assessments at 3, 6 and 9 months after EOT at trial site. This became applicable for subjects without documented investigator-assessed PD and with no initiation of new anti-cancer therapies.<br>4. Saline bolus (10 mL/kg) added as naxitamab premedication<br>5. AESI added: 'Adverse events leading to incomplete dosing during a cycle (meaning total planned dose was not given in a cycle)'<br>6. Per reviewer request, Cohort 2 specified to include subjects without evaluable disease in bone and/or BM at baseline as per independent review
9.0	12.0 (14-Jan-2021)	1. Clarification that AEs must be followed until EOT visit

a. The first subjects were enrolled under Protocol Version 2.0 (04Oct2017). Changes to the protocol version 2 in protocol amendment 1 were all integrated into protocol version 5.0 (12Apr2018). The protocol version 3.0 (13Feb2018) and version 4.0 (09Mar2018) were internal working documents of protocol versions, which were never submitted to regulatory authorities. To avoid misunderstandings with regards to the protocol versions, it was decided that changes with global protocol amendment no. 1 is reflected in protocol version 5.0 (12Apr2018).

Trial 201 protocol deviations

Twelve secondary endpoints were planned for Trial 201, with five reported in this interim analysis manuscript. The remaining seven secondary endpoints are not included in this manuscript, either due to immature data or because they are planned for future publications.

**Secondary endpoints not reported in the interim analysis for Trial 201**
Assessment of the pharmacokinetic profile of naxitamab
Assessment of ADA formation
I.V. opioid use during cycle 1 defined as total usage of i.v. morphine (or equivalent opioid) administered 2 hours before infusion until 4 hours after end of infusion of naxitamab
I.V. opioid use for each cycle during the trial defined as total dose of i.v. morphine (or equivalent opioid) administered 2 hours before infusion until 4 hours after end of infusion of naxitamab
Number of hospitalization days related to naxitamab infusion during cycle 1, defined as number of overnight stays. Hospitalizations required solely for protocol-specified assessments (e.g. PK sampling) or non-medical circumstances are excluded
In patients with positive ADA at trial inclusion, safety will be evaluated by the incidence of AEs and SAEs graded according to CTCAE, version 4.0
Happiness and activity levels measured over time assessed by caretaker.

Abbreviations: ADA, anti-drug antibody; CTCAE, Common Terminology Criteria for Adverse Events; i.v., intravenous; PK, pharmacokinetic; SAEs, serious adverse events.

Reference

1. Park JR, et al. Revisions to the International Neuroblastoma Response Criteria: A consensus statement from the National Cancer Institute clinical trials planning meeting. *J Clin Oncol*. **35**, 2580–2587 (2017)
