# Supplementary material for: The anti-GD2 monoclonal antibody naxitamab plus GM-CSF for relapsed or refractory high-risk neuroblastoma: a phase 2 clinical trial
Source: Nat Commun. 2025 Feb 14;16:1636. doi: 10.1038/s41467-025-56619-x (PMC11828896; doi:10.1038/s41467-025-56619-x)
Supplement: Supplementary file 2 — Reporting Summary [file 41467_2025_56619_MOESM2_ESM.pdf]

nature portfolio

Corresponding author(s): Jaume Mora

Last updated by author(s): 2024/12/19

Reporting Summary

Nature Portfolio wishes to improve the reproducibility of the work that we publish. This form provides structure for consistency and transparency in reporting. For further information on Nature Portfolio policies, see our Editorial Policies and the Editorial Policy Checklist.

Statistics

For all statistical analyses, confirm that the following items are present in the figure legend, table legend, main text, or Methods section.

n/a	Confirmed	
☐	☒	The exact sample size (n) for each experimental group/condition, given as a discrete number and unit of measurement
☐	☒	A statement on whether measurements were taken from distinct samples or whether the same sample was measured repeatedly
☐	☒	The statistical test(s) used AND whether they are one- or two-sided<br>*Only common tests should be described solely by name; describe more complex techniques in the Methods section.*
☒	☐	A description of all covariates tested
☐	☒	A description of any assumptions or corrections, such as tests of normality and adjustment for multiple comparisons
☐	☒	A full description of the statistical parameters including central tendency (e.g. means) or other basic estimates (e.g. regression coefficient) AND variation (e.g. standard deviation) or associated estimates of uncertainty (e.g. confidence intervals)
☐	☒	For null hypothesis testing, the test statistic (e.g. F, t, r) with confidence intervals, effect sizes, degrees of freedom and P value noted<br>*Give P values as exact values whenever suitable.*
☒	☐	For Bayesian analysis, information on the choice of priors and Markov chain Monte Carlo settings
☒	☐	For hierarchical and complex designs, identification of the appropriate level for tests and full reporting of outcomes
☐	☒	Estimates of effect sizes (e.g. Cohen's d, Pearson's r), indicating how they were calculated

Our web collection on statistics for biologists contains articles on many of the points above.

Software and code

Policy information about availability of computer code

Data collection	electronic case report forms compliant with 21 CFR Part 11 regulation
Data analysis	SAS® Version 9.4

For manuscripts utilizing custom algorithms or software that are central to the research but not yet described in published literature, software must be made available to editors and reviewers. We strongly encourage code deposition in a community repository (e.g. GitHub). See the Nature Portfolio guidelines for submitting code & software for further information.

Data

Policy information about availability of data

All manuscripts must include a data availability statement. This statement should provide the following information, where applicable:

- Accession codes, unique identifiers, or web links for publicly available datasets
- A description of any restrictions on data availability
- For clinical datasets or third party data, please ensure that the statement adheres to our policy

Y-mAbs is committed to sharing clinical data with qualified external researchers for independent scientific research. Source data are provided with this paper. The redacted protocol and statistical analysis plan are available as online supplementary materials. The underlying data for this study cannot otherwise be made publicly available due to ethical and legal concerns for patient privacy. Researchers may send requests for the aggregate data from this interim analysis between 3 and 24 months after online publication to datasharing@ymabs.com. Access will be provided following review and approval of a research proposal and execution of a data sharing agreement, consistent with local, state, and federal laws and regulations and the rights of study participants.

Research involving human participants, their data, or biological material

Policy information about studies with human participants or human data. See also policy information about sex, gender (identity/presentation), and sexual orientation and race, ethnicity and racism.

Reporting on sex and gender	Sex was determined based on self-report or parental report. Disaggregated results by sex have been reported where available.
Reporting on race, ethnicity, or other socially relevant groupings	Ethnicity of the population was reported in Table 1.
Population characteristics	Provided in Table 1.
Recruitment	The first patient in the pre-planned interim data set was enrolled on April 11, 2018, and the last patient on December 15, 2021. Patient recruitment and data collection began on April 23, 2018. The data cutoff date for this interim analysis was December 31, 2021.
Ethics oversight	Individual sites obtained appropriate Institutional Review Board approval (Supplementary Table 51). Written informed consent was obtained from legal guardian(s) and/or patient in accordance with local regulations. Patients were not compensated, except for reimbursement of travel expenses as agreed with participating sites.

Note that full information on the approval of the study protocol must also be provided in the manuscript.

Field-specific reporting

Please select the one below that is the best fit for your research. If you are not sure, read the appropriate sections before making your selection.

☒ Life sciences ☐ Behavioural & social sciences ☐ Ecological, evolutionary & environmental sciences

For a reference copy of the document with all sections, see nature.com/documents/nr-reporting-summary-flat.pdf

Life sciences study design

All studies must disclose on these points even when the disclosure is negative.

Sample size	52
Data exclusions	Patients with insufficient/no baseline disease, received radiotherapy at metastatic site, ADA positive, 1st response assessed after cutoff date
Replication	As this is an ongoing Phase 2 trial designed to assess preliminary efficacy and safety of a novel treatment in a specific patient population, replication was not applicable.
Randomization	not randomized
Blinding	not blinded

Reporting for specific materials, systems and methods

We require information from authors about some types of materials, experimental systems and methods used in many studies. Here, indicate whether each material, system or method listed is relevant to your study. If you are not sure if a list item applies to your research, read the appropriate section before selecting a response.

Materials & experimental systems

n/a	Involved in the study
☐	☒ Antibodies
☒	☐ Eukaryotic cell lines
☒	☐ Palaeontology and archaeology
☒	☐ Animals and other organisms
☐	☒ Clinical data
☒	☐ Dual use research of concern
☒	☐ Plants

Methods

n/a	Involved in the study
☒	☐ ChIP-seq
☒	☐ Flow cytometry
☐	☒ MRI-based neuroimaging

Antibodies

Antibodies used	synaptophysin; naxitamab (humanized 3F8)
Validation	as recommended in Burchill et al, 2017; investigational antibody as described in DANYELZA® (naxitamab-gqgk) injection, Prescribing Information 2024

Clinical data

Policy information about clinical studies
All manuscripts should comply with the ICMJEguidelines for publication of clinical research and a completedCONSORT checklist must be included with all submissions.

Clinical trial registration	NCT03363373
Study protocol	A Pivotal Phase 2 Trial of Antibody Naxitamab (hu3F8) and Granulocyte- Macrophage Colony Stimulating Factor (GM-CSF) in High-Risk Neuroblastoma Patients with Primary Refractory Disease or Incomplete Response to Salvage Treatment in Bone and/or Bone Marrow
Data collection	electronic case report form compliant with 21 CFR Part 11 regulation
Outcomes	Primary: ORR during the naxitamab treatment period, defined as centrally assessed CR or PR according to the International Neuroblastoma Response Criteria (INRC) at independent review. Secondary endpoints included: DOR, PFS, OS

Magnetic resonance imaging

Experimental design

Design type	Used for diagnostic imaging only
Design specifications	*Specify the number of blocks, trials or experimental units per session and/or subject, and specify the length of each trial or block (if trials are blocked) and interval between trials.*
Behavioral performance measures	*State number and/or type of variables recorded (e.g. correct button press, response time) and what statistics were used to establish that the subjects were performing the task as expected (e.g. mean, range, and/or standard deviation across subjects).*

Acquisition

Imaging type(s)	*Specify: functional, structural, diffusion, perfusion.*
Field strength	*Specify in Tesla*
Sequence & imaging parameters	*Specify the pulse sequence type (gradient echo, spin echo, etc.), imaging type (EPI, spiral, etc.), field of view, matrix size, slice thickness, orientation and TE/TR/flip angle.*
Area of acquisition	*State whether a whole brain scan was used OR define the area of acquisition, describing how the region was determined.*

Diffusion MRI ☐ Used ☐ Not used

Preprocessing

Preprocessing software	*Provide detail on software version and revision number and on specific parameters (model/functions, brain extraction, segmentation, smoothing kernel size, etc.).*

Normalization	*If data were normalized/standardized, describe the approach(es): specify linear or non-linear and define image types used for transformation OR indicate that data were not normalized and explain rationale for lack of normalization.*
Normalization template	*Describe the template used for normalization/transformation, specifying subject space or group standardized space (e.g. original Talairach, MNI305, ICBM152) OR indicate that the data were not normalized.*
Noise and artifact removal	*Describe your procedure(s) for artifact and structured noise removal, specifying motion parameters, tissue signals and physiological signals (heart rate, respiration).*
Volume censoring	*Define your software and/or method and criteria for volume censoring, and state the extent of such censoring.*

Statistical modeling & inference

Model type and settings	*Specify type (mass univariate, multivariate, RSA, predictive, etc.) and describe essential details of the model at the first and second levels (e.g. fixed, random or mixed effects; drift or auto-correlation).*
Effect(s) tested	*Define precise effect in terms of the task or stimulus conditions instead of psychological concepts and indicate whether ANOVA or factorial designs were used.*

Specify type of analysis: ☐ Whole brain ☐ ROI-based ☐ Both

Statistic type for inference<br>(See Eklund et al. 2016)	*Specify voxel-wise or cluster-wise and report all relevant parameters for cluster-wise methods.*
Correction	*Describe the type of correction and how it is obtained for multiple comparisons (e.g. FWE, FDR, permutation or Monte Carlo).*

Models & analysis

n/a	Involved in the study
☐	☐ Functional and/or effective connectivity
☐	☐ Graph analysis
☐	☐ Multivariate modeling or predictive analysis

Functional and/or effective connectivity	*Report the measures of dependence used and the model details (e.g. Pearson correlation, partial correlation, mutual information).*
Graph analysis	*Report the dependent variable and connectivity measure, specifying weighted graph or binarized graph, subject- or group-level, and the global and/or node summaries used (e.g. clustering coefficient, efficiency, etc.).*
Multivariate modeling and predictive analysis	*Specify independent variables, features extraction and dimension reduction, model, training and evaluation metrics.*
